# Estimates of Japanese Encephalitis mortality and morbidity: A systematic review and modeling analysis

**DOI:** 10.1371/journal.pntd.0010361

**Published:** 2022-05-25

**Authors:** Yuwei Cheng, Nhat Tran Minh, Quan Tran Minh, Shreya Khandelwal, Hannah E. Clapham

**Affiliations:** 1 Saw Swee Hock School of Public Health, National University of Singapore and National University Health System, Singapore, Singapore; 2 Oxford University Clinical Research Unit, Ho Chi Minh City, Vietnam; 3 Biological Sciences Department, University of Notre Dame, Notre Dame, Indiana, United States of America; Australian Red Cross Lifelood, AUSTRALIA

## Abstract

**Background:**

Japanese Encephalitis (JE) is known for its high case fatality ratio (CFR) and long-term neurological sequelae. Over the years, efforts in JE treatment and control might change the JE fatality risk. However, previous estimates were from 10 years ago, using data from cases in the 10 years before this. Estimating JE disease severity is challenging because data come from countries with different JE surveillance systems, diagnostic methods, and study designs. Without precise and timely JE disease severity estimates, there is continued uncertainty about the JE disease burden and the effect of JE vaccination.

**Methodology:**

We performed a systematic review to collate age-stratified JE fatality and morbidity data. We used a stepwise model selection with BIC as the selection criteria to identify JE CFR drivers. We used stacked regression, to predict country-specific JE CFR from 1961 to 2030. JE morbidity estimates were grouped from similar study designs to estimate the proportion of JE survivors with long-term neurological sequelae.

**Principal findings:**

We included 82 and 50 peer-reviewed journal articles published as of March 06 2021 for JE fatality and morbidity with 22 articles in both analyses. Results suggested overall JE CFR estimates of 26% (95% CI 22, 30) in 1961–1979, 20% (95% CI 17, 24) in 1980–1999, 14% (95% CI 11, 17) in 2000–2018, and 14% (95% CI 11, 17) in 2019–2030. Holding other variables constant, we found that JE fatality risk decreased over time (OR: 0.965; 95% CI: 0.947–0.983). Younger JE cases had a slightly higher JE fatality risk (OR: 1.012; 95% CI: 1.003–1.021). The odds of JE fatality in countries with JE vaccination is 0.802 (90% CI: 0.653–0.994; 95% CI: 0.62–1.033) times lower than the odds in countries without JE vaccination. Ten percentage increase in the percentage of rural population to the total population was associated with 15.35% (95% CI: 7.71, 22.57) decrease in JE fatality odds. Ten percentage increase in population growth rate is associated with 3.71% (90% CI: 0.23, 7.18; 95% CI: -0.4, 8.15) increase in JE fatality odds. Adjusting for the effect of year, rural population percent, age of JE cases, and population growth rate, we estimated that there was a higher odds of JE fatality in India compared to China. (OR: 5.46, 95% CI: 3.61–8.31). Using the prediction model we found that, in 2000–2018, Brunei, Pakistan, and Timor-Leste were predicted to have the highest JE CFR of 20%. Bangladesh, Guam, Pakistan, Philippines, and Vietnam had projected JE CFR over 20% for after 2018, whereas the projected JE CFRs were below 10% in China, Indonesia, Cambodia, Myanmar, Malaysia, and Thailand. For disability, we estimated that 36% (min-max 0–85) JE patients recovered fully at hospital discharge. One year after hospital discharge, 46% (min-max 0%-97%) JE survivors were estimated to live normally but 49% (min-max 3% - 86%)till had neurological sequelae.

**Conclusion:**

JE CFR estimates were lower than 20% after 2000. Our study provides an updated estimation of CFR and proportion of JE cases with long-term neurological sequelae that could help to refine cost-benefit assessment for JE control and elimination programs.

## Author summary

Japanese Encephalitis (JE) is known for its high case fatality ratio (CFR) and long-term neurological sequelae. Although JE fatality and morbidity risk might change as the characteristics of the population change with the strengthened surveillance scales, expanded immunization coverage, and healthcare improvements, there have not been any updates in the estimates for JE mortality and morbidity estimates for 10 years. In this paper, we made updated estimates of the JE CFR and the proportion of JE survivors with long-term neurological sequelae by performing a systematic review and developing statistical and machine learning models. We estimated JE CFR decreased over time, with estimates of 26% (95% CI 22, 30) in 1961–1979, 20% (95% CI 17, 24) in 1980–1999, 14% (95% CI 11, 17) in 2000–2018, and 14% (95% CI 11, 17) in 2019–2030. countries without JE vaccination, younger JE cases, higher population growth rate, and lower rural population percentage were associated with higher JE CFR. We estimated that 36% (min-max 0–85) JE patients recovered fully at hospital discharge. One year after hospital discharge, 46% (min-max 0%-97%) JE survivors were estimated to live normally but 49% (min-max 3% - 86%) JE patients still had neurological sequelae. The insights gained will be important in evaluating and updating current JE disease burden among all endemic areas and effectively channeling resources to most needed areas.

## Introduction

Japanese Encephalitis Virus (JEV) belongs to the genus Flavivirus, family Flaviviridae, and consists of five phylogenetically separate genotypes (GI-GV)[1]. JEV is transmitted between Culex mosquitoes and amplifying vertebrate hosts, primarily pigs. Humans are dead-end hosts in the JEV transmission cycle [2]. Japanese Encephalitis (JE) caused by JEV is the leading cause of human encephalitis in Southeast Asia [3] and Western Pacific regions. 24 countries are considered to have JEV transmission risk, and an estimated 3 billion people live in areas at risk for JE infection [2]. The global mortality burden of JE was estimated to be 25 thousand deaths [4] in 2015, and 20 thousand in 2011[5]. JE associated DALYs lost were estimated to be 3161, 3022, 1412, and 1012 in Thailand, China, Vietnam, and Indonesia [6]. In these four countries, 58%-70% of DALYs lost were due to post-JE disability.

To evaluate JE disease burden, the estimates of case-fatality ratio (CFR) computed as the proportion of reported JE cases that die and long-term morbidity ratio computed as the proportion of JE patients that had life-long neurological sequelae were crucial. JE CFR estimates ranging from 20% to 30% and long-term morbidity rates ranging from 30% to 50% were used by most JE disease burden models [3, 4, 6]. The range of JE CFR estimates was derived in 2008 based on 13 studies [7–19] covering 8 JE endemic countries and areas including Bangladesh, India, Japan, Korea, Nepal, Taiwan, Thailand, and Vietnam. Likewise, 14 studies [7, 12, 17, 18, 20–29] encompassing China, India, Vietnam, and Thailand were selected to construct long-term JE morbidity rates.

Despite large variations observed in JE CFRs across time and space, currently there is no available country-specific CFR estimates for accurate mortality burden estimation. Reported JE CFRs were as low as 0% in Thailand in 1972[30], in Korea in 1982[31], in Japan in 1994[32], in India in 2004[33], and in Myanmar in 2013[34]. Reported JE CFRs were as high as 100% in Korea and India (estimates ranging from 1962–2006 [8, 31, 33, 35–37]).

JE fatality risk might change as the characteristics of JE susceptible populations evolve with the strengthened surveillance system [38, 39], expanded immunization coverage [38], increased age of infection [38–41], advanced case confirmation methods [42, 43], and improved healthcare. Even though there is no effective treatment to cure the disease [44], multiple types of JE vaccine including inactivated vaccine, live attenuated vaccine, and chimeric vaccine, targeting different JE strains and phenotypes, were developed for the purpose of better disease control [45]. As of 2016, 13 out of 24 JE endemic countries had implemented JE immunization program, nationally or sub-nationally. The proportion of JE endemic countries that conducted JE surveillance and routinely tested JE cases has increased from 21% (5/24) in 2012 to 92% (22/24) in 2016[38, 39].

Time-series analysis in China and India showed a decreasing pattern in JE CFR [46, 47]. However, there have not been any updates in the estimated values for JE CFR estimates. Although the estimate of long-term morbidity ratio for JE is available, it is not stratified by the severity level and derived from studies published two decades ago. In addition, it was not clear how the estimate was constructed [48]. Without precise and timely estimates of JE fatality and long-term morbidity, there is continued uncertainty about the JE disease burden and the effect of JE vaccination.

Therefore, in this paper we updated the JE mortality and morbidity estimates with data collated from a systematic review and built models to estimate the CFR for countries and time periods which we do not have data.

## Methodology

### Case fatality

We conducted a systematic review of JE case fatality; we used the search terms “fatality” or “mortality” or “lethal” or “death” in all fields with “Japanese encephalitis” in the title or abstract to find all available articles in PubMed. We screened all titles and abstracts. For those with age-stratified case-fatality data, we retrieved the full-texts, and the articles were read by two independent reviewers. If abstracts were not available, full-texts articles were assessed and examined by the two independent researchers (S1 Checklist). We included articles in English with data on CFR of JE infections in humans after 1960 when more than 20 JE cases and 1 JE death are reported. Thus, we removed articles that were studies on vaccine impact evaluation, cost-benefit analysis of the JE immunization program, and biological studies that focused on molecular research. Studies that were not written in English, studies that were not on humans, studies with no case-fatality data, and studies that had duplicated data resources from another included paper were also excluded. For each study we extracted the number of cases, deaths, and computed case-fatality rate as the ratio between deaths and confirmed cases for JE overall, by year, and by age where possible. This subgrouping constitutes a JE CFR record. Information on catchment areas for the study, case confirmation methods, regional vaccination programs, outbreaks, and the type of surveillance systems which collected and reported the JE cases were also collected. For country-level socioeconomic variables potentially correlated to JE CFR, we collected population density, GDP growth rate, GDP per capita, under-5 mortality rate, population growth rate, percentage of rural population in total population, and urban population size from the World Bank Open Data [49] (S1 Table).

As co-variate data was available for 20 of the endemic countries, we included 20 countries in our analysis (Bangladesh, Brunei, Cambodia, China, Guam, India, Indonesia, Japan, Laos PDR, Malaysia, Myanmar, Nepal, Pakistan, Papua New Guinea, Philippines, South Korea, Sri Lanka, Thailand, Timor-Leste and Vietnam).

To identify variables associated with variation in JE CFR (inference model), we made inference using generalized linear regression. This analysis was conducted for all 20 endemic countries. We included Year, Length of Study, Age Lower (Minimum age of the reported JE patients), Age Upper (Maximum age of the reported JE patients), JE cases, deaths, JE diagnostic method, Type of Surveillance System, Geographical location, Outbreak (1, if the study was conducted during an outbreak), Youth (if the study was conducted among individuals less than 18 years of age), Vaccination (whether the country has a vaccination program), GDP per capita and annual growth rate, Under 5 mortality rate, Population Growth, Population Density, Rural population (as a % of total population) a Urban Population (in millions).

We used the logit-transformed CFR as the response variable to fit the model using the above covariates. The best model with the lowest BIC was selected by stepwise model selection

### Model formula for the selected model:

$$\log\left(\frac{CFR}{1-CFR}\right)=\beta_{0}+\beta_{1}AgeLower+\beta_{2}Year+\beta_{3}\log\left(Population\;growth\right)+\beta_{4}\log(Rural\;Population\;(\%))+\beta_{5}Vaccination+\sum_{c=1}^{C}\beta_{Tc}1_{T_{i}=c}$$

(S1 File). We checked for normal assumption of residual distribution. We also checked for influential points.

After using the inference model to identify the JE CFR drivers, we then used six models to project the CFR for endemic places from 1961 to 2018: 1) LASSO, 2) Principal Component Regression (PCR), 3) Gradient Boosting Machine (GBM), 4) Neural Network (NN), 5) Multiple Linear Regression (MLR), and 6) a weighting-based algorithm separately to stack CFR predictions from the base learners (the base learners are models 1–4). The prediction error is most likely to reduce when the stacked base learners are uncorrelated and have high variability.

We used the variables identified from the inference model population density, GDP annual growth rate, under-5 mortality, GDP per capita, population growth rate, percentage of rural population in total population, urban population size, diagnostic method (fixed as WHO’s JE case definition: JE IgM antibody in CSF or serum as confirmed by MAC-ELISA on patients with acute encephalitis syndrome), type of surveillance system (fixed as national surveillance), and regional vaccination status as predictors. Using the prediction model, estimates were calculated the CFR for 1961–1979, 1980–1999 and for 2000–2018. Due to lack of availability of socioeconomic data required for the model we exclude 6 (Myanmar, Cambodia, Vietnam, Timor-Leste, Laos and Guam) out of 20 endemic countries for the years 1961–1979; 3 countries (Guam, Myanmar, and Timor-Leste) for the years 1980–1999 and Guam for 2000–2018. We calculated the binomial log likelihood of these predictions across all data points (S1 File).

We also use this model (model 6) to project the CFR for future years post 2018. For this projection model, we assumed that the JE immunization status remains unchanged in all the countries for which the model was used. We included all 20 endemic countries for the projection.

We calculated the prediction average for each country and year using this model. We bootstrapped the collated dataset 2000 times.

We conducted a sensitivity analysis for the inference models by excluding data from India or China to evaluate the robustness of our model to these countries from which there was a large amount of data (S7 Table). We also ran the prediction model using the subset of data that had the WHO case definition to assess how this might influence the results.

### Disability

For JE disability review, we used the search terms “weakness” or “discharge” or “recover” or “abnormal” or “impair” or “disorder” or “disability” in all fields with “Japanese encephalitis” or “Viral Encephalitis, Japanese” in the title or abstract to find all available articles in PubMed. We screened all titles, abstracts, and articles. We included only studies written in English that reported data on the proportion of JE survivors that develop disability after JE infection. Thus, we excluded studies with JE infection not on humans, studies on irrelevant pathogens, studies with focus on suggestions for traveler’s safety, studies on JE vaccine effectiveness evaluation, studies not in English, studies with no available disability data, and case reports that only examine clinical symptoms and disease development of only one JE patient (S2 Checklist). We extracted JE records that contained information on year of the study, the numbers of reported JE cases, JE fatality, cases with complete recovery, and cases with sequelae, age of the JE patients, outcome measurement time, and disability assessment method, where possible. We estimated the proportion of long-term neurological sequelae by age and depending on the definition of neurological sequelae used in the respective study. We also analyzed the proportion of JE patients who had complete recovery at or after hospital discharge by age, where possible. For papers included in both analyses (CFR and disability), we assessed the relationship between JE CFR and the proportion of neurological sequelae at hospital discharge using a linear regression.

All analyses were conducted by using the R statistical software [50], version 4.0.4.

## Results

### Mortality

#### The data collated

**Fig 1:**
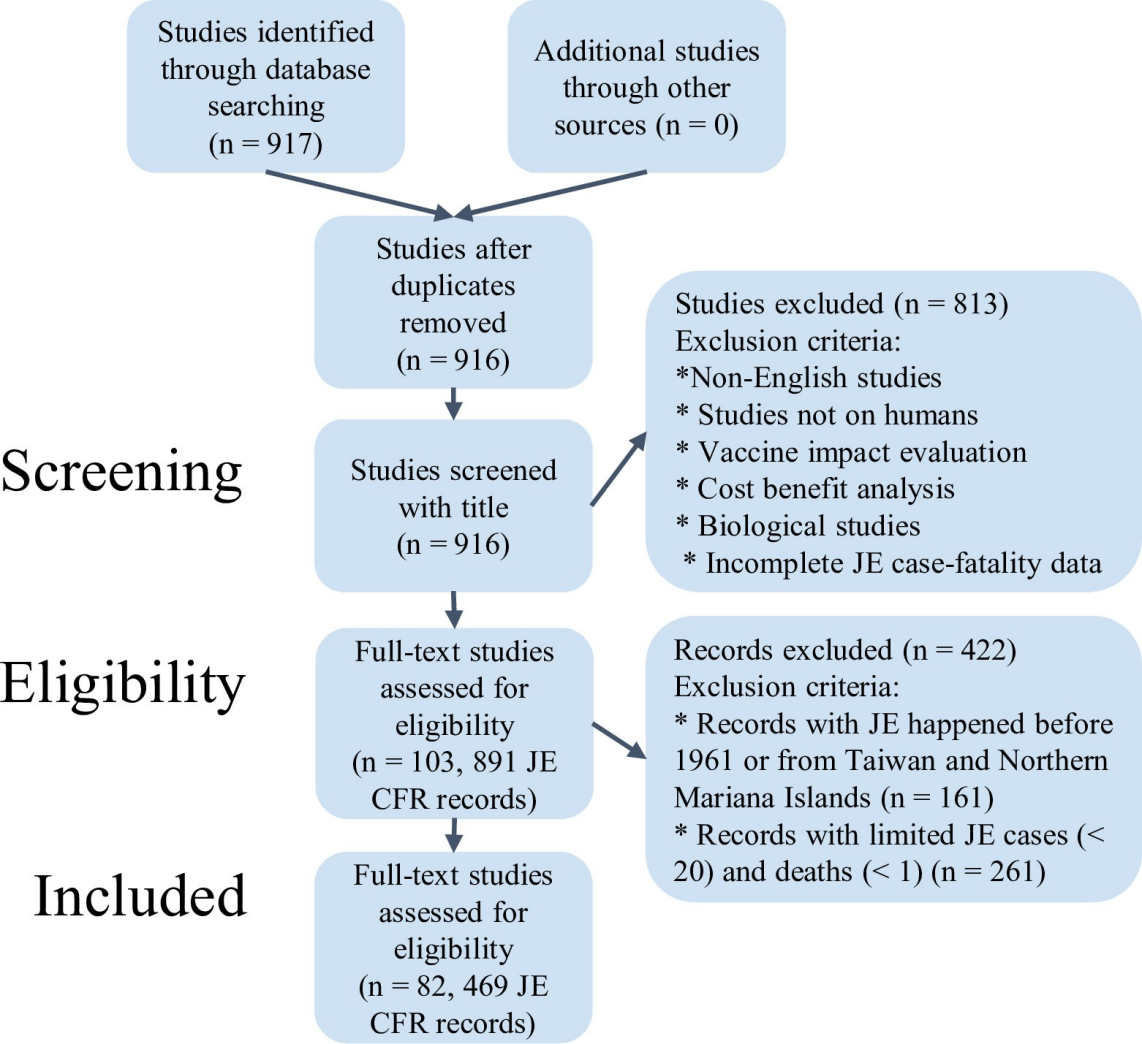
Flowchart describing the systematic review procedure searching for Japanese encephalitis (JE) case-fatality ratio (CFR) records using the PubMed database.

On March 06, 2021, we identified 917 studies in the initial literature review (Fig 1). After retrieving and analysing 103 full-text papers, 891 age-stratified JE CFR records were extracted. Due to the unavailability of associated country-level socioeconomic factors which were required for our model predictors, 161 records that from before 1961, or in Taiwan or Northern Mariana Islands were removed. 244 records were removed due to very few reported cases (less than 20) and 17 records with 0 JE fatality were excluded for the ease of taking logit transformation. Finally, 82 studies from which 469 distinct age and year stratified JE CFR records were collated and included in the analysis (S1 Included Characteristics, data in S2 File).

The total dataset collated using the literature review (Table 1) comprised of 406,818 JE cases and 49,225 JE deaths before the year 2000 with a mean unweighted CFR of 28%. After the year 2000, there were 371,853 cases and 36,889 deaths, with a lower mean CFR of 15%. In the studies which reported JE cases, most of them were reported by the national surveillance system. Sentinel hospital surveillance system was the second largest venue where cases were reported, though these ranged in size from one to several hospitals. The sample size of the reported patients in each study varied considerably, ranging from 20 to 68,427 with a median value of 180. The majority (91%) of analyzed JE cases happened in China or India (Table 1).

**Table 1:** Table of summary statistics of age-stratified Japanese encephalitis (JE) case-fatality observations obtained from the systematic review. Spatial and temporal distribution of the collected JE cases, deaths, and case-fatality ratios (CFR) were presented. India, Korea, and China were the top three countries with the most observed JE records. Other countries from which records were included are Bangladesh, Cambodia, Hong Kong, Indonesia, Japan, Malaysia, Myanmar, Nepal, Thailand, and Vietnam. The unweighted mean is the mean of the CFR calculated across all records in our dataset for a particular time period and country.

Factor	Total	Reported within 1961–1999				Reported within 2000–2016			
		India	China	Korea	Others	India	China	Korea	Others
**Age stratified CFR records (% of total)**	469	124 (26.4)	14 (3.6)	77 (16.4)	67 (14.2)	97 (20.7)	38 (8.1)	3 (0.6)	46 (9.8)
**Number of JE cases (% of total)**	778,672	77,504 (9.9)	277,037 (35.6)	10,491 (1.3)	41,787 (5.4)	123,569 (15.9)	234,083 (30.1)	78 (0.01)	14,123 (1.8)
**Number of JE deaths (% of total)**	86,114	23,390 (27.1)	18,219 (21.1)	2,874 (3.3)	4,742 (5.5)	26,035 (30.2)	9,515 (11)	20 (0.02)	1,319 (1.5)
**Unweighted mean of JE CFR (%)**	23.4	33.4	5.7	31.2	20.9	23.2	4.8	25.6	8.8

**Fig 2:**
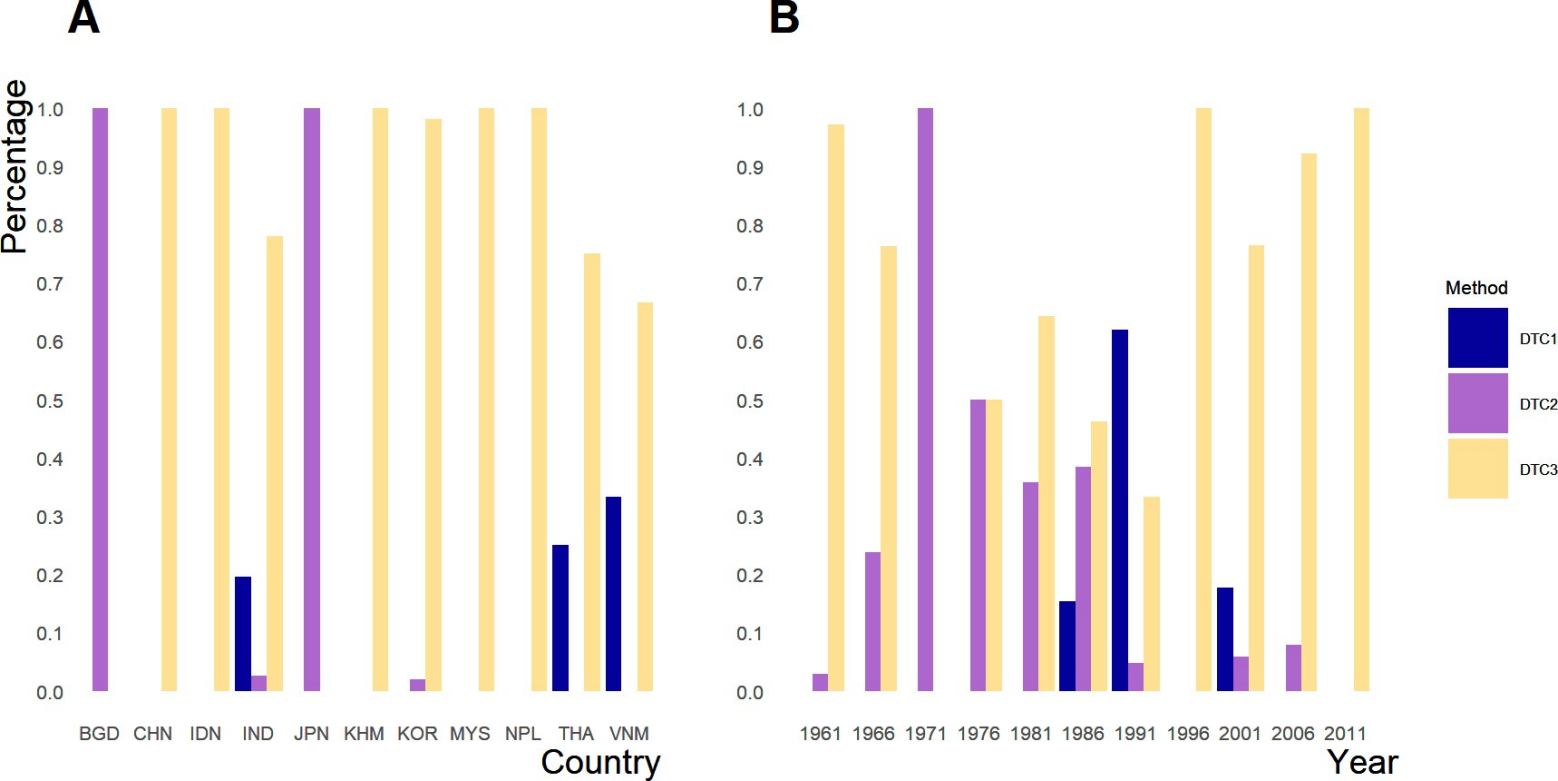
Distribution of Japanese encephalitis (JE) diagnostic methods used by collated JE case-fatality ratio (CFR) records with clear case definition by geographical locations (Panel A) and time (Panel B). DTC is the abbreviation of diagnostic testing category. The colors represent each method, with dark blue for cases confirmed by virus isolation, antigen or pathogen detection (DTC1), purple for cases confirmed by detection of neutralization antibody in cerebrospinal fluid (CSF) or serum (DTC2), and yellow for cases consistent with WHO JE case definition: JE Immunoglobulin M (IgM) antibody in CSF or serum as confirmed by MAC-ELISA on patients with acute encephalitis syndrome (DTC3). Only the diagnostic methods for laboratory confirmed JE cases collected from studies in our analysis were plotted.

There were 41% (192/469) observations in which laboratory confirmation method was reported (Fig 2A). The remaining observations were either diagnosed only based on clinical symptoms or had inadequate information to determine the case confirmation method. For the laboratory-confirmed cases, most observations (146/192) used the WHO case definition: JE IgM antibody in CSF or serum as confirmed by MAC-ELISA on patients with acute encephalitis syndrome. This method was the main diagnostic method in all countries except Bangladesh, where detecting neutralization antibody in CSF or serum was the leading JE case confirmation method (Fig 2A). JE case confirmation by the detection of neutralization antibody in cerebrospinal fluid (CSF) or serum was used less frequently over time (Fig 2B); 24% (46/192) observations confirmed JE cases by JE virus, antigen, or pathogen and neuralization antibody in cerebrospinal fluid (CSF) or serum.

#### Exploring variables associated with CFR

The inference model was used to identify the variables associated with the JE CFR. The best model has a BIC of 1132.67. The BIC of the full model is 1214.08 and the BIC of the null model is 1467.84. The residual diagnostic plot (S1 File), does not show any nonlinear trend and non-constant variance. The plot is also symmetric with respect to 0, thus we did not find any issues in the model fitting.

Holding other variables constant, we found that JE fatality risk decreased over time (OR: 0.965; 95% CI: 0.947–0.983) (Table 2). Younger JE cases were associated with higher JE fatality risk (OR: 1.012; 95% CI: 1.003–1.021). JE fatality risk odds in JE immunized countries is 0.802 (90% CI: 0.653–0.994; 95% CI: 0.62–1.033) times of odds in JE unimmunized countries (Table 2). Ten percentage increase in the percentage of rural population to the total population was associated with 15.35% (95% CI: 7.71, 22.57) decrease in JE fatality odds. Ten percentage increase in population growth rate is correlated with 3.71% (90% CI: 0.23, 7.18; 95% CI: -0.4, 8.15) increase in JE fatality odds (Table 2). Adjusting for the effect of year, rural population percent, age of JE cases, and population growth rate, the likelihood of JE fatality risk in India is higher than in China. (OR: 5.46, 95% CI: 3.61–8.31) (Table 2).

**Table 2:** Table of coefficients of predictors that were used in the inference model to fit Japanese encephalitis case-fatality ratio collated from systematic review. Mean, and 95% confidence interval (CI) were presented for both the model and bootstrap estimates. *** indicates that odds ratio of the factor is different from 1 with a significance level of 95%; ** indicates that odds ratio of the factor is different from 1 with a significance level of 90%; Ref stands for reference category.; Rural population % is the percentage of rural population in the country; Age Lower is the minimum age of a laboratory confirmed JE case.

Predictors		Mean	Mean (boot)	95% CI (theoretical)	95% CI (Bootstrap)	90% CI (Bootstrap)
**Country**	India***	1.69	1.70	(1.27, 2.11)	(1.28, 2.12)	(1.35, 2.05)
	Japan	-0.50	-0.51	(-1.42, 0.42)	(-1.45, 0.38)	(-1.29, 0.23)
	South Korea	0.16	0.16	(-0.38, 0.69)	(-0.38, 0.68)	(-0.31, 0.60)
	Nepal***	0.97	0.98	(0.47, 1.46)	(0.48, 1.46)	(0.56, 1.39)
	Other countries and areas (Bangladesh, Cambodia, Hong Kong, Indonesia, Myanmar, Malaysia)***	0.74	0.74	(0.14, 1.34)	(0.13, 1.29)	(0.24, 1.20)
	Thailand**	0.34	0.34	(-0.066, 0.74)	(-0.064, 0.73)	(0.002, 0.68)
	Vietnam	0.14	0.14	(-0.32, 0.60)	(-0.31, 0.60)	(-0.24, 0.52)
	China (Ref)					
Year***		-0.035	-0.036	(-0.55, -0.16)	(-0.054, -0.017)	(-0.051, -0.02)
Rural population % (log scale)***		-1.53	-1.54	(-2.27, -0.79)	(-2.256, -0.77)	(-2.16, -0.91)
Age Lower***		0.012	0.012	(0.0026, 0.022)	(0.003, 0.021)	(0.004, 0.02)
Population growth (%) (log scale)**		0.38	0.37	(-0.046, 0.80)	(-0.04, 0.82)	(0.023, 0.72)
Vaccination**	1	-0.22	-0.22	(-0.47, 0.033)	(-0.48, 0.032)	(-0.43, -0.06)
	0 (Ref)					

In sensitivity analysis, we ran the JE inference model without JE records from China and India one at a time and in both cases we observe that lowest Age of JE patients and the population growth (in %) were no longer significant at 5% (S2 Table).

#### Predicting for countries without data and into the future

We use the prediction model to estimate the CFR for countries without data and also to project the CFR for the future years after 2018. We identified that with population density, population growth, GDP per capita, GDP growth rate, under-5 mortality, rural population (%), urban population size, country indicators, vaccination, and diagnostic testing method as predictors, stacking with the weighting algorithm as meta learner (model 6) was the best model for CFR prediction and projection (Table 3). Even though LASSO regression (model 1) had the largest median log likelihood, the distribution of the log likelihood of LASSO (model 1) was heavily left tailed. The performance of LASSO (model 1) in the lower 2.5% quantile was 1000 times worse compared to its performance in the upper 2.5% quantile. On the contrary, the performance of model 6 in the lower 2.5% quantile was less than 10 times worse compared to its performance in the upper 2.5% quantile. The performance of stacking with weighting algorithm as meta learner (model 6) had a smaller variance thus, we chose stacking as the best performance model.

**Table 3:** Table of binomial log likelihood calculated from 6 models in 2000 bootstrapped datasets sampled from the dataset collated from systematic review. In each bootstrapped dataset we computed binomial log likelihood for each model. For each model summary statistics of the distribution of log likelihood was presented. Abbreviations: LASSO: Least Absolute Shrinkage and Selection Operator; PCR: Principal Component Regression; GBM: Gradient Boosting Machine; NN: Neural Network; MLR: Multiple Linear Regression.

Model	Log likelihood	
	Predictors	
	Median	95% CI
**1. LASSO**	-9034	(-7006221, -7260)
**2. PCR**	-98871	(-293247, -10374)
**3. GBM**	-24177	(-51047, -11261)
**4. NN**	-140811	(-2703911, -26363)
**5. Stacking (MLR)**	-24799	(-56287, -11407)
**6. Stacking (Weighting algorithm)**	-19274	(-73941, -8886)

**Fig 3:**
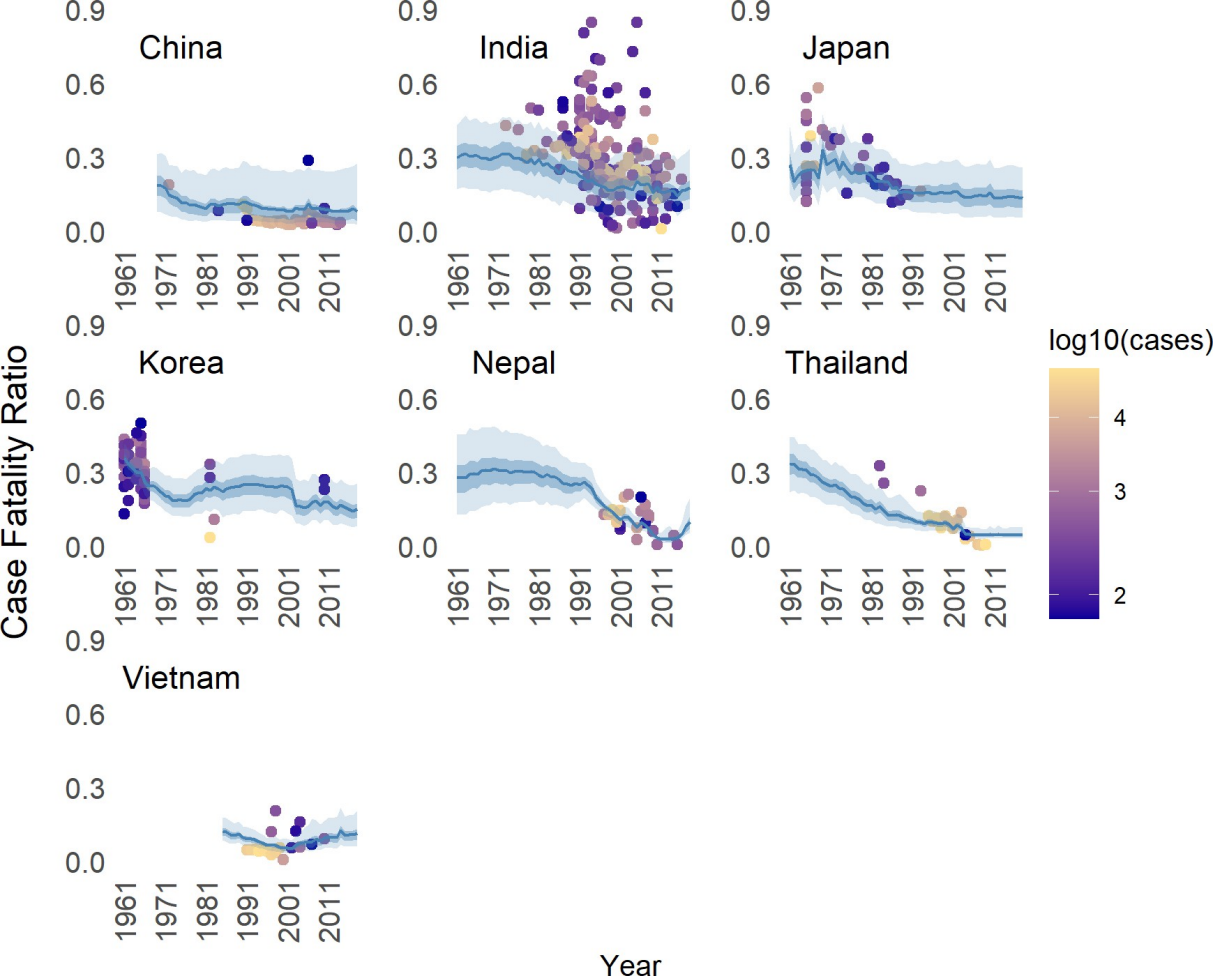
The Japanese encephalitis (JE) case-fatality ratio (CFR) records and the predicted JE CFRs for each JE endemic country with more than 10 JE CFR records over time using the best model (model 6). In all plotted JE endemic countries, the solid line and the lighter ribbon represent the mean predicted JE CFR with its 95% confidence interval. Darker ribbon shows the region of 1^st^ quartile and 3^rd^ quartile of predicted JE CFR. Dots represent the JE CFR records with color dependent on the value of log_10_ (the number of reported JE cases).

**Fig 4:**
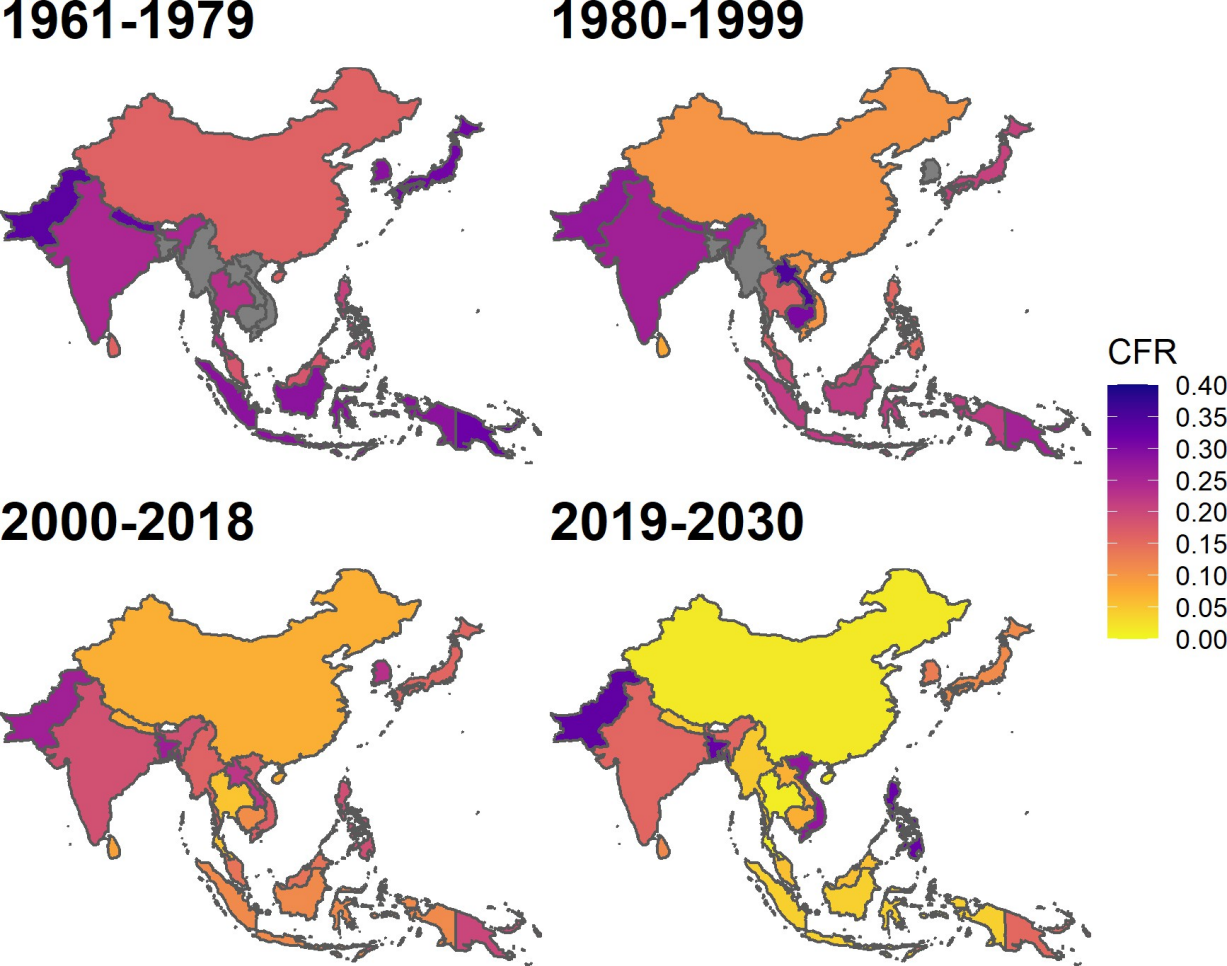
Spatial and temporal distribution of Japanese encephalitis (JE) National case-fatality ratio (CFR) estimates in JE (endemic countries) from 1961–2030. Countries without estimates were filled with grey (values for these estimates are given in Table 4). The underlying map was generated using open source data from https://www.naturalearthdata.com/.

Based on JE CFR estimates and records, and using model 6 we project CFR over time and space, as shown in Fig 3. Using the stacking model with weighting algorithm as meta learner (model 6) and without year as predictor the mean global predicted JE CFR was 26% (95% CI 22, 30) in 1961–1979, 20% (95% CI 17, 24) in 1980–1999, 14% (95% CI 11, 17) in 2000–2018, and the mean projected JE CFR of 14% (95% CI 11, 17) in 2019–2030 (Table 4). By country, Bangladesh, Lao PDR, and Pakistan had JE CFR above 30% before 2000 and for all countries we estimated a considerable decrease in CFR estimates after 2000 (Fig 4). China, Sri Lanka, Nepal, Thailand, and Vietnam had CFR lower than 10% in 2000–2018. We also estimated the CFR for countries with JEV transmission risk but had for which there were no studies reporting CFR. The estimated JE CFR in 2000–2018 for Brunei was 20%, Sri Lanka 7%, Pakistan 20%, Papua New Guinea 15%, and Timor-Leste 20%. Even though the projected JE CFR in 2019–2030 increased considerably from the previous two decades in Bangladesh and Vietnam (Table 4), the associated confidence intervals were fairly wide, and the projected estimates were not statistically different from the predicted estimate in 2000–2018.

**Table 4:** Table of estimated Japanese encephalitis (JE) case-fatality ratio (CFR) estimated by the stacking model (model 6) without including year as predictor for 20 out of 24 JE endemic countries from 1961 to 2030. Country-specific and year-stratified JE CFRs and their associated 95% CI were presented. Areas with N/A values for JE CFR predictions had at least one unavailable socioeconomic feature.

JE endemic countries	Case-fatality ratio (95% CI)			
	Predicted			Projected
	1961–1979	1980–1999	2000–2018	2019–2030
**Overall**	26 (21, 31)	20 (17, 24)	14 (11, 17)	14 (11, 17)
**Bangladesh**	35 (15, 54)	30 (13, 49)	18 (8, 35)	28 (10, 51)
**Brunei**	27 (10, 45)	25 (13, 41)	20 (10, 34)	16 (7, 30)
**China**	14 (5, 28)	10 (4, 26)	9 (3, 25)	5 (3, 12)
**Guam**	N/A	N/A	N/A	24 (15, 37)
**Indonesia**	28 (16, 41)	16 (8, 31)	11 (5, 23)	7 (4, 13)
**India**	28 (16, 41)	23 (8, 39)	17 (8, 39)	14 (7, 24)
**Japan**	25 (14, 40)	17 (8, 31)	14 (6, 27)	15 (8, 26)
**Cambodia**	N/A	29 (17, 42)	11 (4, 23)	7 (4, 16)
**South Korea**	24 (13, 37)	24 (14, 36)	18 (9, 31)	18 (10, 29)
**Lao PDR**	N/A	30 (16, 43)	18 (8, 31)	11 (4, 23)
**Sri Lanka**	14 (7, 21)	6 (4, 10)	7 (3, 17)	10 (4, 21)
**Myanmar**	N/A	N/A	14 (7, 27)	6 (3, 21)
**Malaysia**	21 (11, 35)	21 (12, 36)	18 (9, 32)	9 (4, 21)
**Nepal**	30 (16, 47)	24 (12, 38)	7 (2, 15)	10 (5, 21)
**Pakistan**	36 (23, 49)	34 (20, 49)	20 (11, 33)	23 (13, 37)
**Philippines**	29 (19, 40)	23 (13, 36)	15 (12, 33)	22 (12, 36)
**Papua New Guinea**	24 (13, 36)	21 (12, 33)	15 (8, 27)	17 (8, 29)
**Thailand**	26 (14, 40)	12 (7, 21)	5 (3, 11)	4 (3, 9)
**Timor-Leste**	N/A	N/A	20 (11, 32)	16 (9, 27)
**Vietnam**	N/A	9 (5, 15)	9 (4, 17)	22 (12, 33)

### Sensitivity analyses

To understand the impact of the JE case definitions, we ran the model on subset of the data with case definition (WHO case definition). We ran model 6 on JE CFR records with clear case definition. The estimated JE CFR in 1961–1979 was 28% (95% CI: 22, 33), JE CFR in 1980–1999 was 25% (95% CI: 19, 30), JE CFR in 2000–2018 was 18% (95% CI: 14, 23), and JE CFR in 2019–2030 was 14% (95% CI: 12, 17). The decreasing trend in JE CFR estimates is the same here and there are no statistical differences in all estimates compared to the estimates in the main analysis (S3 Table).

To understand if there was still variation in time not captured by our co-variates, we explored including year as a predictor into the prediction model. Model performance based on binomial likelihood after including year as predictor was worse (S4 Table). We did not use year as a predictor as it is not time, but other socioeconomic variables that we include in the model are changing in time that would be influencing the output. We therefore chose to use the model without year.

### Morbidity

**Fig 5:**
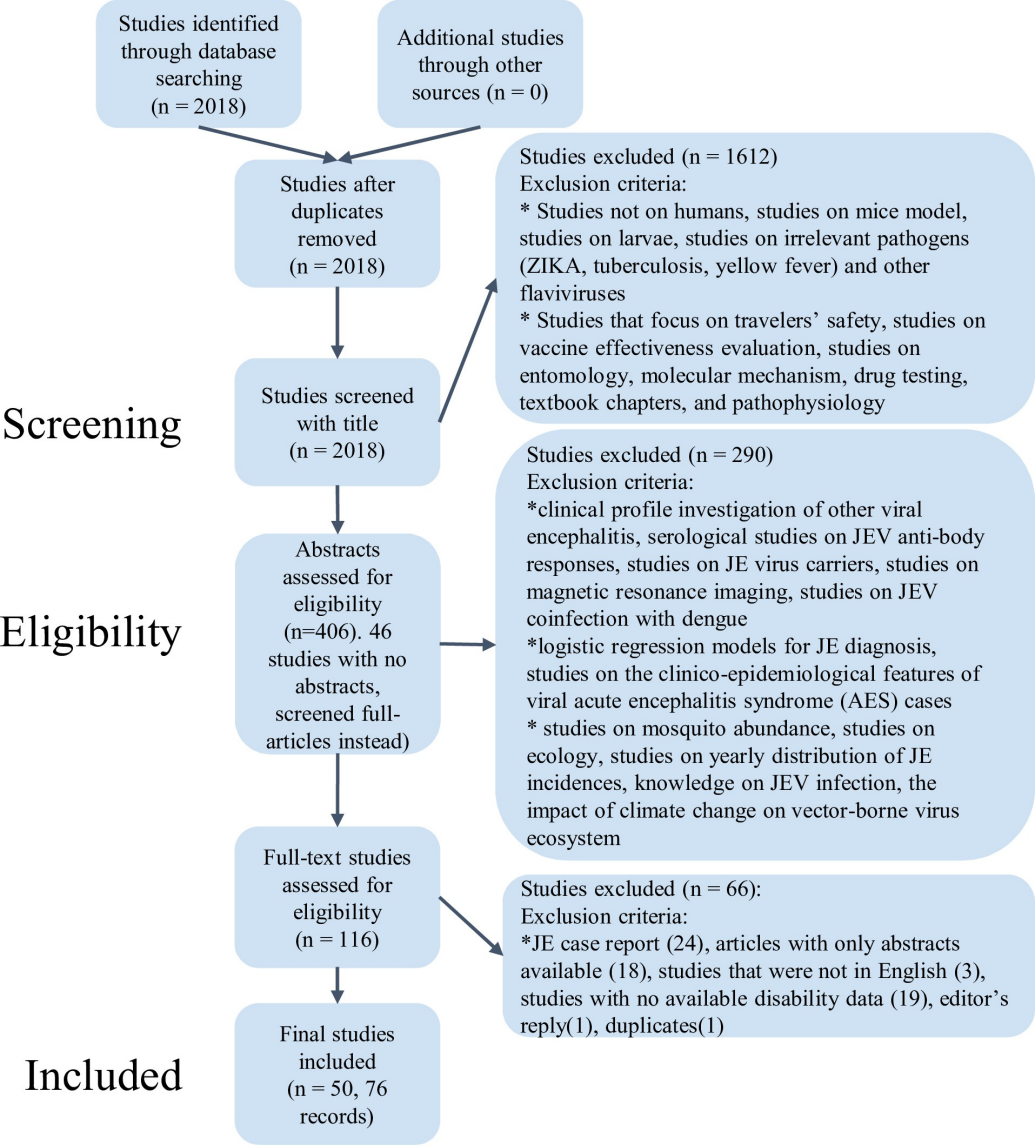
Flowchart describing the systematic review procedure searching for Japanese encephalitis (JE) neurological sequelae records using PubMed database.

We identified 2018 articles in the initial literature review (Fig 5). We excluded 1612 studies through title screening as they were not studies on humans, studies on other flaviviruses, or safety suggestions for travelers. We analyzed 360 abstracts and 46 full articles that did not have available abstracts post which 290 articles were removed as they did not contain data on JE disability. We then analyzed full-texts of the remaining 98 studies and 18 abstracts. We excluded 18 abstracts and 19 articles because the disability data are not available. We further excluded 24 JE case reports (these reports focus on clinical symptoms and disease prognosis for a single patient, which is not helpful in assessing the proportion of JE survivors that develop long-term disability). We removed 1 editor’s reply, 1 duplicate, and 3 studies that were not in English. Finally, we had a set of 50 articles that led to 76 JE disability records. 22 of these studies were also included in the CFR analysis.

The collated studies covered 14 JE endemic countries and areas including Bangladesh, Cambodia, China, India, Indonesia, Japan, South Korea, Lao PRD, Malaysia, Nepal, Philippine, Taiwan, Thailand, and Vietnam. Most studies were carried out in India (17/50), China (6/50), or Vietnam (6/50). Half of the studies (26/50) analyzed JE cases occurring after 2000. The identified studies had heterogenous follow up times; 22 studies only evaluated the patients’ outcome at hospital discharge and 28 studies evaluated the outcomes at least one month after hospital discharge. Among these 28 studies, 9 studies evaluated the patients’ outcome more than one year after hospital discharge. The study size varied from 4 patients to 144 patients, with 30 as the median study size.

Outcomes of JE patients were evaluated differently across the studies. All studies documented the proportion of JE cases that had neurological deficits. Within these studies, 17 studies documented the type of neurological deficits in detail but did not grade the sequelae severity [20, 24, 26, 28, 51–63]. 33 studies graded the severity of the neurological deficits but adopted a range of evaluation tools. 11 articles categorized the outcome as poor (bedridden), partial (needing help with daily activities), or complete recovery (able to perform daily activities independently)[64–74]. 8 articles used Liverpool Outcome Score (LOS) to assess basic motor and self-care skills and simple cognitive and behavioral functions [75–82]. Patients who were unable to live independently (score 2) were classified as severe. Patients with moderate (score 3) or minor sequelae (score 4) were able to make independent living. 5 articles also grouped patient outcome into severe/moderate/minor yet based on the degree of independent functioning and controllability of seizures or the author’s subjective assessment [17, 83–86]. 4 articles adopted Modified Rankin Scale to measure the degree of disability or dependence in the daily activities [87–90]. 3 articles used Glasgow Coma Scale [91–93] and the remaining 2 articles used the Whitely’s method [94, 95].

On average using all studies definition of “completely recovered” we found 36% (min-max 0–85) of JE patients fully recovered at hospital discharge (S6 Table). For outcome measured more than one year after discharge, 46% (min-max 0–97) of JE survivors had completely recovered. The proportion of children patients (< = 18 years old) with complete recovery at discharge was 44% (min-max 9–85), higher than the estimate for adults (23%, min-max 0–67). But there was a wide disparity in adult patients’ outcomes one year after discharge reported by different studies.

**Fig 6:**
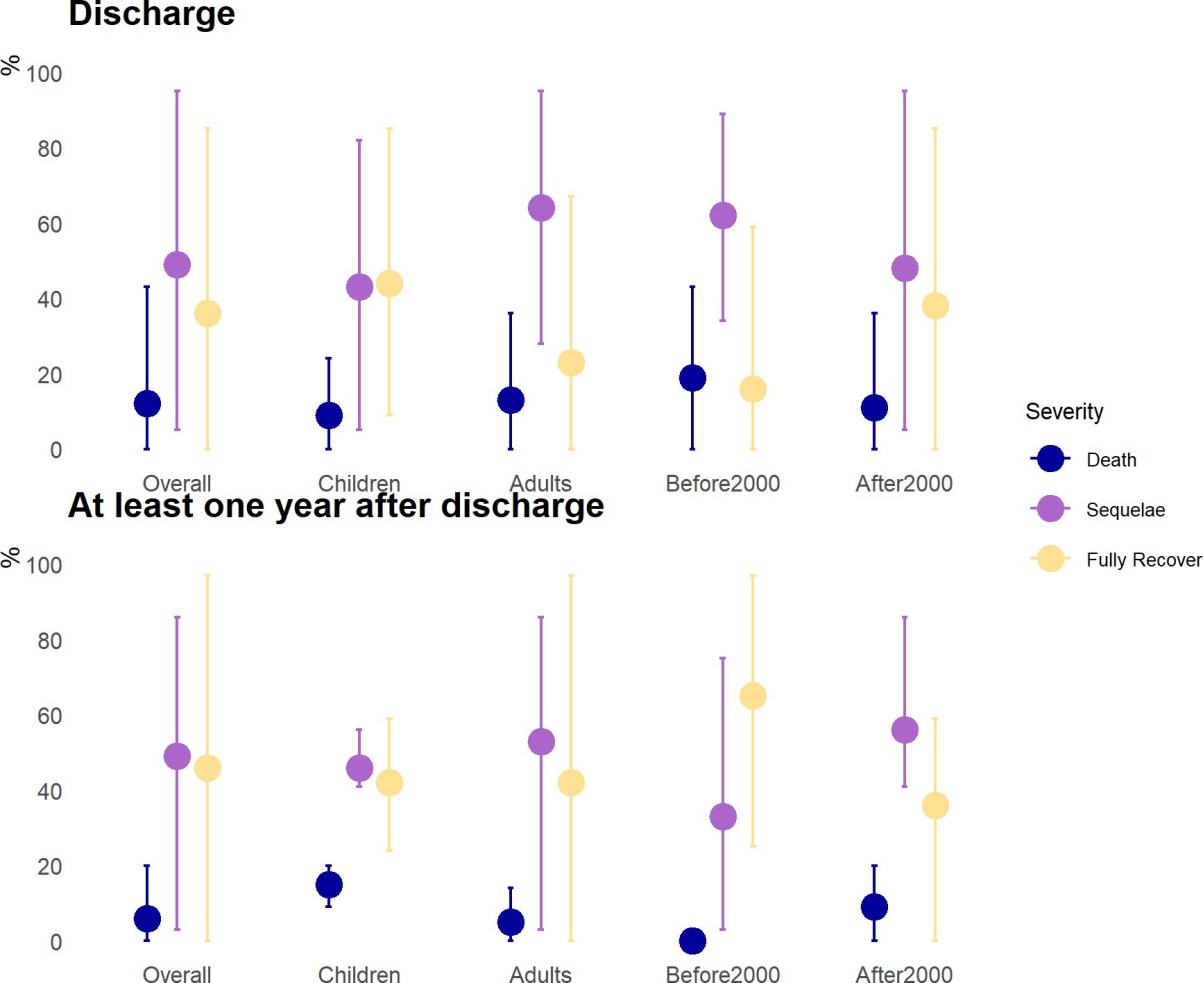
Japanese encephalitis (JE) disease outcomes by age and measurement time. Disease outcomes are stratified by measured at hospital discharge and at least one year after hospital discharge and four subgroups: JE cases before 2000, cases after 2000, adults (JE patients older than 18 years old), and children (JE patients 18 years old and younger). Note that JE death measured at hospital discharge occur at the acute stage of JE infection, while JE death measured at least one year after hospital discharge only contains death occurred during follow up.

**Fig 7:**
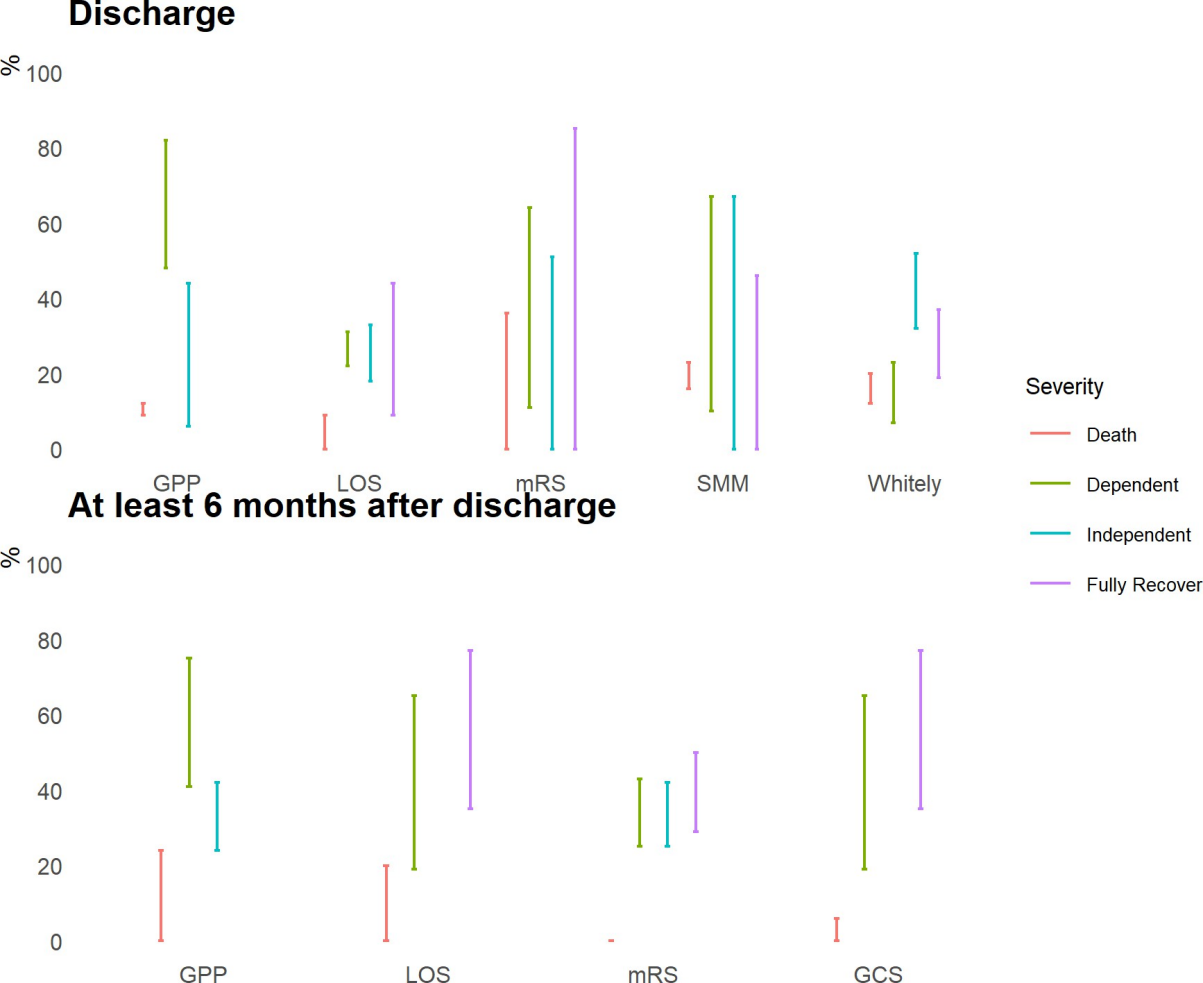
Range of Japanese encephalitis (JE) neurological deficits severity level by outcome measurement time and assessment tools. We define dependent as JE survivors who need assistance to perform daily activities and independent as JE patients who can live independently. JE patients with complete recovery show no symptoms of any neurological sequelae. Death measured at least 6 months after hospital discharge excludes death occurred during the acute stage of JE infection. Abbreviations: GPP (Good/Partial/Poor), Liverpool Outcome Score (LOS), Modified Rankin Scale (mRS), SMM (Severe/Moderate/Minor), Glasgow Coma Scale (GCS).

We further estimated that on average 49% (min-max 5–95) of JE patients suffered from some neurological sequalae at hospital discharge compared to a similar estimate, 49% (min-max 3% - 86%) when the outcome was measured at least one year after hospital discharge (Fig 6). From studies that graded neurological sequelae severity (33 studies), we further simplified the severity level into two categories, dependent and independent, depending on whether JE patients were able to perform daily activities by themselves. We estimated that 7%-82% JE patients were unable to live independently at discharge (Fig 6). However, the disease severity assessed by different evaluation tools varied significantly. 2 studies that utilized Whitely’s method showed 7%-23% of JE survivors had severe neurological deficits and required assistance to perform daily activities (Fig 7). For another 2 studies that used good/partial/poor as the evaluation metric suggested that 48% - 82% of JE patients were either bed-ridden or needing help with daily activities (Fig 7). When the outcome was measured at half a year after hospital discharge, the studies estimated that 3%-75% of patients were unable to live independently (Fig 7).

Within the included follow-up studies, 54% (15/28) studies had patients lost to follow up. Refusal to participation, no contact information, relocation, and leave against medical advice were the top four reasons for the lost to follow up. 7 studies [28, 54, 56, 77, 80, 94, 96] had lost to follow up greater than 20%. Within these articles, 5 studies [28, 54, 56, 77, 80] had follow-up period greater than 1 year, one study [54] alerted refusal to participation due to poor health condition may generate bias to the study’s result. Long travel distance and unwillingness to receive medical examination were the main refusal reasons for the rest of studies and might not affect the estimate (S6 Table).

For studies that were included both in the fatality review and disability review, we ran a linear regression to assess whether case-fatality rate during the acute stage was associated with the proportion of JE patients with neurological sequelae at hospital discharge. As the p value was 0.32 (>0.05), no evidence suggested the existence of linear correlation between the CFR and the proportion of cases with neurological sequelae. (S1 Fig)

## Discussion

In this paper, we updated JE disease severity estimates using data gathered from the systematic review. We estimated JE CFR in 1961–1979, 1980–1999, 2000–2018, and 2019–2030 of 26%, 20%, 14%, and 14% respectively. We estimated 49% (min-max 3%-86%) JE survivors had long-term neurological sequelae and different recovery patterns between children and adults were observed.

Our CFR estimates before 2000 were consistent with Fischer et al. ’s (2008) estimate. Fischer et al. [48] narrowed down the range of JE CFR to 20%-30% by reviewing 13 studies from 8 JE endemic areas published between 1949–2005 [89]. By comprehensively reviewing 82 studies published as of 2021 March 06 from 12 of the JE endemic areas, our study went beyond providing a range of CFR by developing a prediction and projection model to contribute to understand changes and variation in CFR, the variables that are correlated with CFR, and up-to-date estimates.

Our analysis estimated that the reduced JE CFR estimate was correlated with the expanded coverage of JE immunization (Table 2). JE immunization coverage expanded rapidly after 2000. 67% (10/15) countries and areas introduced the immunization programs nationally or sub-nationally after 2000[42], including China and India where most analyzed JE patients came from. The correlation of JE vaccination with reduced JE CFR would be consistent with vaccination which not only reduced infection, but also moderated the disease severity [45]. Mouse-brained-derived inactivated JE vaccine was showed to be effective in reducing fatality of confirmed cases in 1-14-year-old children by 19.3%[97]. However, it is also possible that the relationship we observe with vaccination is also due to changes in surveillance which were brought with the JE vaccination program [38] or other changes in healthcare that occur concurrently with vaccination, though we did attempt to account for these in the analysis, we could not completely.

In our analysis, we found that population demographics was associated with JE fatality risk and patient’s access to healthcare might be the underlying driver for this correlation. Our result showed that country with higher population growth, denser population, higher proportion of urban population had higher JE fatality risk. In countries that the growth of local healthcare capacity falls behind the population growth rate, patient’s waiting time before getting the treatment might be prolonged, thus worsening the final outcome. Likewise, a populous district or a metropolis is likely to have low individual medical resources, consequently having a negative impact on the fatality rate.

There was minimal information available on the age of cases. We did see a significant, though small, difference in the estimated CFR by age (higher for the studies that included older ages). However, there is information in the way the data from these studies is currently presented. This is an important area of future study as children aged 15 years old and below was the primary targeted population for JE immunization [98], the main age group of the confirmed JE cases in many countries shifted from young children to adults [18, 99, 100]. In countries with childhood JE immunization program, this is something to be aware of, as JE mortality burden in adults might increase with this shifting pattern in the age of infection.

We analyzed country level variation in CFR along with the changes in time. After accounting for the impact of vaccination status, demographic structures, surveillance scales, employed JE diagnostic methods, and other available socioeconomic features, effect of country and time still remained statistically significant (Table 2). Therefore, there are unexplained variations after addressing the current factors and indicated the presence of unobserved features, for instance, improved critical JE case management over time, differences in genotype, differences in healthcare seeking, and heterogenous case-reporting rate which were also crucial in understanding JE CFR variations across time and space.

It was unclear why the projected values from the data in some countries (for example Vietnam, see Fig 3) an increasing trend was observed, which is unusual given the widespread vaccination there. Further analysis would be useful to understand why this is occurring.

Consistent with Fischer et al. (2008)’ estimate that 30%-50% JE survivor had long-term sequelae, our result showed 49% of JE survivors had at least one type of neurological deficits at least one year after at hospital discharge, indicating a permanent damage caused by JE infection. Post-JE disability is the major component of the disease burden and is important to be part of the calculation when assessing whether to include JE vaccination in vaccination programs. However, there was a range of estimates of proportion of cases that experience JE neurological deficits from different studies. This may be due to the inherent differences in the severity of the cases, hospital’s capacity to manage such cases, or the different ranges and focus of the assessment tools used. As well as the disabilities recorded, it was interesting to note that results from two studies presented 20% and 24% JE patients died after hospital discharge, which may mean there is an underestimate of the CFR for JE, however, there was no direct evidence that death was attributed to JE infection.

Our result found that at hospital discharge, a higher proportion (44% min-max: 9–85) of children fully recovered from JE infection compared to adult patients (23% min-max 0–67) (S6 Table). However, one year after hospital discharge, the proportions of children and adult patients who had a complete recovery converged. Patients with different ages may have different disease recovery speeds.

By providing granular risk estimates for JE mortality and morbidity for specific time periods and countries, our study serves as a stepping stone towards improving JE mortality and morbidity estimation, especially for the GAVI burden of disease assessments, including their tracking process toward JE control, elimination, and mortality and morbidity reduction. This is also useful for countries when they are making decisions about the introduction or continued use of JE vaccination. From a broader perspective, our work proposes a practical framework to estimate country-and-time specific CFR for a disease by using publicly available data. For a disease with large variations in observed CFRs but with only fixed estimates available, our method is more likely to generate precise estimations for disease burdens, which facilitates effective resource channeling and disease control.

In addition to those discussed above relating to specific variables, there were some overall limitations of this analysis. Firstly, our result was constrained by the varying quality of different articles included. Only a few studies screened contained cases and deaths stratified by age thus restricting the analysis by age. The lack of availability of age-stratified data might lead to a biased dataset for model fitting. Several JE cases and deaths collated were aggregated across multiple years. Thus, we were unable to uncover any between year variation in JE CFR and the middle point year was used to index the overall CFR. The accuracy of the projected estimates could be limited by the heterogenous quality of the projected socioeconomic features as well.

Secondly, our model was only able to address across-country variations in JE CFR. Vaccination coverage, healthcare quality and economic development in different regions of a country may vary significantly, resulting within-country variations. Although many studies we screened included catchment areas detailed at province or district level, only country-level socioeconomic features were accessible. Thus, CFR that we gathered were assumed to be nationally representative.

Although 13 countries and areas were included in the modelling, 91% of analyzed JE patients came from China and India. The identified global pattern between CFR and socioeconomic features could be dominated by the results in these two countries. We also excluded records from our analysis that reported 0 JE deaths which may cause an upward bias in our estimates, even though our estimates are lower than the currently available estimate of 20%.

Thirdly, our dataset included records from studies that used multiple methods of JE diagnosis and the heterogeneity across the studies might lead to a biased dataset used in the model construction. We also excluded records from our analysis that reported 0 JE deaths due to inconsistency in the residuals when these were included in the modelling process (Fig D in S1 File). Hence, our estimates may overestimate the CFR and can be considered as an upper bound which is still lower than the current available estimates of JE CFR.

## Conclusion

Our analysis enabled us to draw several conclusions. The estimated CFR after 2000 was 14% and it is lower than 20% previously estimated. On average 49% of JE survivors suffered long-term neurological sequelae. The presence of JE immunization program, under-5 mortality, and the age of infection were important predictors to understand spatial-temporal variations in JE CFRs.

## Supporting information

S1 Checklist Case fatality PRISMA checklist. (DOCX)

S2 Checklist Disability PRISMA checklist. (DOCX)

S1 Fig Scatter plot of proportion of fatality and sequelae at hospital discharge. Dot size was scaled according to the number of patients in the observed records.
(TIF)

S1 Table Table of detailed description of estimators, predictors, and projectors that were used in the Japanese Encephalitis (JE) case-fatality ratio modelling. (DOCX)

S2 Table Sensitivity analysis: Table of odds ratio of predictors that were used in the regression to fit Japanese encephalitis case-fatality ratio with complete information on age for the inference model without using data from China or India. Mean, Mean (Bootstrap), and 95% confidence interval (CI) of the odds ratio were presented. *** indicates that odds ratio of the factor is different from 1 with a significance level of 95%. Ref stands for reference category.
(DOCX)

S3 Table Table of predicted and projected JE case-fatality ratio (CFR) estimated by the stacking model fitted on JE records with clear case definition for 20 out of 24 JE endemic countries from 1961 to 2030. Country-specific and year-stratified JE CFRs and their associated 95% CI were presented.
(DOCX)

S4 Table Table of binomial log likelihood calculated from 6 models with year as predictor in 2000 bootstrapped datasets sampled from the dataset collated from systematic review. In each bootstrapped dataset we computed binomial log likelihood for each model. For each model summary statistics of the distribution of log likelihood was presented. Abbreviations: LASSO: Least Absolute Shrinkage and Selection Operator; PCR: Principal Component Regression; GBM: Gradient Boosting Machine; NN: Neural Network; MLR: Multiple Linear Regression.
(DOCX)

S5 Table Table of predicted and projected JE case-fatality ratio (CFR) estimated by LASSO fitted on JE records with clear case definition for 20 out of 24 JE endemic countries from 1961 to 2030. Country-specific and year-stratified JE CFRs and their associated 95% CI were presented.
(DOCX)

S6 Table Table of dropout analysis. (DOCX)

S7 Table Sensitivity analysis: Tables of predicted and projected CFRs for all countries from 1961–1979, 1980–1999, 2000–2018 using model 6 when fitted without using data from China and India one at a time. Mean CFR, Lower and Upper bounds of the 95% C.I. are presented.
(DOCX)

S1 File Detailed methodology description. (DOCX)

S2 File Data. (XLSX)

S1 Included Characteristics Table of detailed description of all the records extracted from the studies included in the CFR literature review. (DOCX)
