# Supplementary figures and images for: Estimates of Japanese Encephalitis mortality and morbidity: A systematic review and modeling analysis
Source: PLoS Negl Trop Dis. 2022 May 25;16(5):e0010361. doi: 10.1371/journal.pntd.0010361 (PMC9173604; doi:10.1371/journal.pntd.0010361)

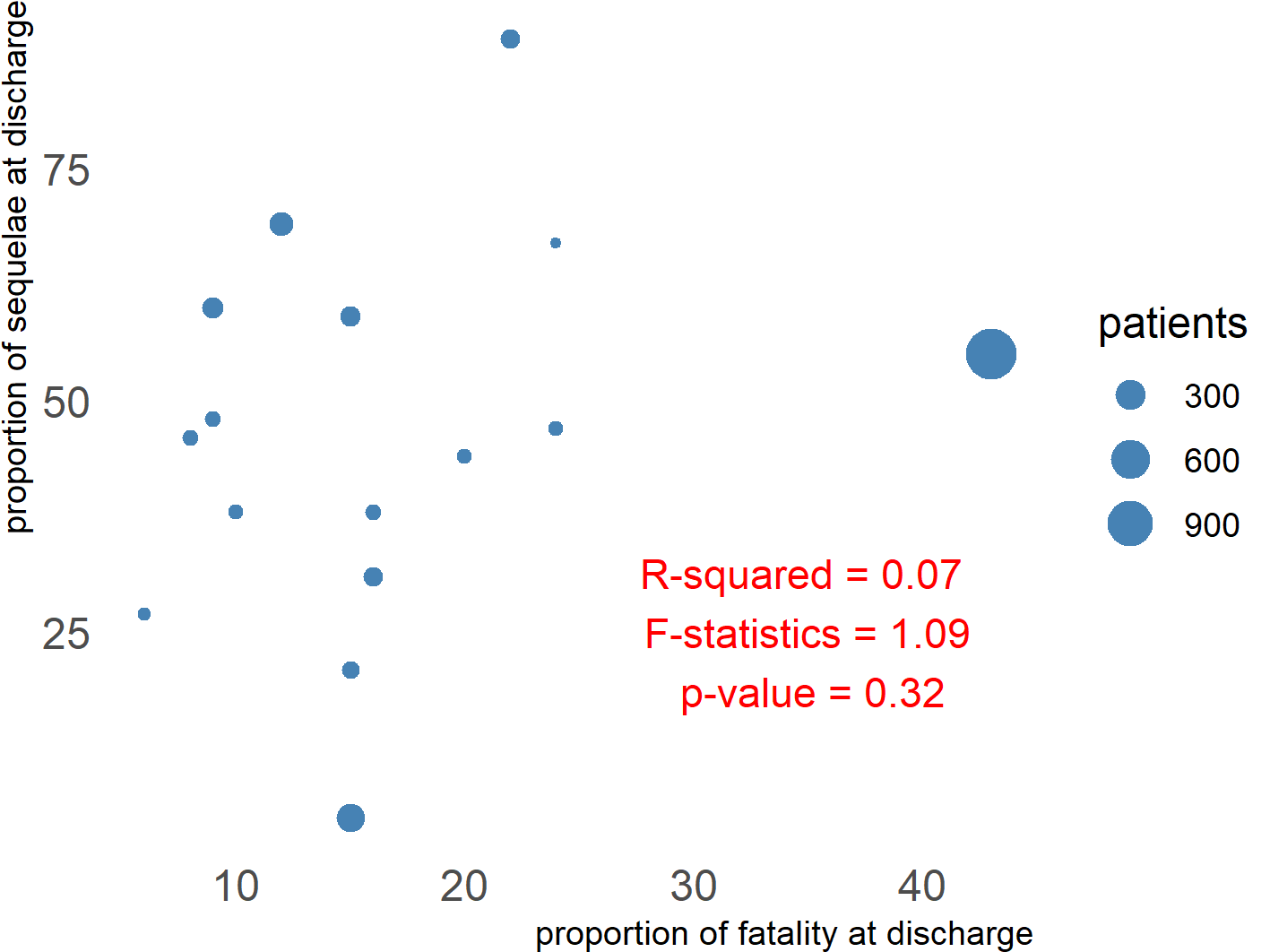

Supplement: S1 Fig — Dot size was scaled according to the number of patients in the observed records. (TIF) [file pntd.0010361.s003.tif]
