# Supplementary material for: Estimates of Japanese Encephalitis mortality and morbidity: A systematic review and modeling analysis
Source: PLoS Negl Trop Dis. 2022 May 25;16(5):e0010361. doi: 10.1371/journal.pntd.0010361 (PMC9173604; doi:10.1371/journal.pntd.0010361)
Supplement: S1 Table — (DOCX) [file pntd.0010361.s004.docx]

**S1 Table. Table of detailed description of estimators, predictors, and projectors that were used in the Japanese Encephalitis (JE) case-fatality ratio modelling**

| **Variable** | **Description** | **Summary Statistics** | **Data Type** | **Data Source** | **Usage** |
| --- | --- | --- | --- | --- | --- |
| **Year** | Time when JE cases were reported. We used the midpoint year when JE cases were aggregated across multiple years | Min-Max: 1961-2016 (Median: 1997) | Integer | Extracted from systematic review | Estimator |
| **Length of study** | If collated JE case data are aggregated by year, the value of this variable will be one. Otherwise, the value of this variable will be the number of aggregated years. | Min-Max: 1-39  (Median: 1) |  |  | Estimator |
| **Age lower** | Minimum age of the reported JE patients. If the age information is not disclosed, we assume that all JE patients are between 0-99 years old. | Min-Max: 0-70  (Median: 0) |  |  | N/A |
| **Age upper** | Maximum age of the reported JE patients | Min-Max: 1-99  (Median: 99) |  |  | N/A |
| **Cases** | The number of reported JE cases | Min-Max: 20-68427  (Median: 180) |  |  | Estimator weights |
| **Deaths** | The number of reported JE deaths | Min-Max: 1-7460  (Median: 42) |  |  | N/A |
| **CFR** | Case-fatality ratio computed by deaths over cases | Min-Max: 0.005-0.85  (Median: 0.22) | Numeric |  | Response variable |
| **JE Diagnostic method** | DTC1: virus isolation, antigen and pathogen detection  DTC2: detection of neutralization antibody in cerebrospinal fluid (CSF) or serum  DTC3: detection of anti-JEV Immunoglobulin M (IgM) in CSF or serum into one group. (Reference level)  DTC4: JE cases confirmed by clinical symptoms together or laboratory confirmation method was not disclosed | DTC1 (N = 18)  DTC2 (N = 28)  DTC3 (N = 146)  DTC4 (N = 277) | Categorical |  | Estimator, predictor |
| **Type of surveillance system that collected or reported JE cases** | SSC1: national surveillance (Reference level)  SSC2: surveillance by many hospitals  SSC3: surveillance by a single hospital  SSC4: surveillance by medical colleges  SSC5: Not disclosed | SSC1 (N=154)  SSC2 (N=19)  SSC3 (N=28)  SSC4 (N=23)  SSC5 (N=245) |  |  | Estimator, predictor |
| **Geographical location of the study** | Bangladesh, Cambodia, China (Reference level), India, Indonesia, Hong Kong, Japan, Korea, Malaysia, Myanmar, Nepal, Thailand, Vietnam, | Bangladesh (N=1), Cambodia (N=3), China (N=55), Hong Kong (N=1), India (N=221), Indonesia (N=3), Japan (N=41), Korea (N=80), Malaysia (N=1), Myanmar (N=1), Nepal (N=26), Thailand (N=18), Vietnam (N=18) |  |  | Estimator, predictor |
| **Outbreak** | Whether the JE cases were reported during a JE outbreak | 12 records were collected during a JE outbreak |  |  | Estimator |
| **Youth** | Whether all JE cases are below year 18 | 49 records with JE cases aged 18 years old and below | Indicator |  | Estimator |
| **Vaccination** | Whether the country has national or subnational JE immunization program | 83 records were collected at places that had JE immunization program |  | (Bharucha et al., 2020) | Estimator, predictor, and projector |
| **GDP per capita** | GDP per capita is gross domestic product divided by midyear population | Min-Max: 94-35766  Median: 442 | Numeric | World Bank Data | Estimator, predictor, and projector |
| **GDP annual growth rate** | Annual percentage growth rate of GDP at market prices based on constant local currency. | Min-Max: -8-15  Median: 7 |  |  |  |
| **Under-5 mortality rate** | Under-five mortality rate is the probability per 1,000 that a newborn baby will die before reaching age five, if subject to age-specific mortality rates of the specified year | Min-Max: 4-203  Median:78 |  |  |  |
| **Population growth** | Annual population growth rate for year t is the exponential rate of growth of midyear population from year t-1 to t, expressed as a percentage | Min-Max: -0.04-2.97  Median: 1.69 |  |  |  |
| **Population density** | Population density is midyear population divided by land area in square kilometers. | Min-Max: 46-1052  Median: 300 |  |  |  |
| **Rural population (% of total population)** | Rural population refers to people living in rural areas as defined by national statistical offices. | Min-Max: 18-88  Median: 70 |  |  | Estimator, and predictor |
| **Urban population (in million)** | The number of people living in urban areas as defined by national statistical offices | Min-Max: 2.7-740.2  Median: 236.3 |  |  |  |
