# Supplementary material for: Estimates of Japanese Encephalitis mortality and morbidity: A systematic review and modeling analysis
Source: PLoS Negl Trop Dis. 2022 May 25;16(5):e0010361. doi: 10.1371/journal.pntd.0010361 (PMC9173604; doi:10.1371/journal.pntd.0010361)
Supplement: S2 Table — Mean, Mean (Bootstrap), and 95% confidence interval (CI) of the odds ratio were presented. *** indicates that odds ratio of the factor is different from 1 with a significance level of 95%. Ref stands for reference category. (DOCX) [file pntd.0010361.s005.docx]

**S2 Table A. Sensitivity analysis: Table of odds ratio of predictors that were used in the regression to fit Japanese encephalitis case-fatality ratio with complete information on age for the inference model without using data from China.** Mean, Mean (Bootstrap), and 95% confidence interval (CI) of the odds ratio were presented. *** indicates that odds ratio of the factor is different from 1 with a significance level of 95%. Ref stands for reference category.

| **Predictors** | | Mean | Mean  (boot) | **95% CI**  **(Bootstrap)** | **90% CI**  **(Bootstrap)** |
| --- | --- | --- | --- | --- | --- |
| **Country** | Japan*** | -3.67 | -3.654 | (-5.27, -2.03) | (-5.003,-2.334) |
|  | South Korea*** | -1.99 | -1.98 | (-2.851, -1.165) | (-5.003, -2.334) |
|  | Nepal*** | -0.69 | -0.703 | (-1.43, -0.07) | (-1.279, -0.166) |
|  | Other countries and areas (Bangladesh, Cambodia, Hong Kong, Indonesia, Myanmar, Malaysia)* | -0.88 | -0.88 | (-1.541, -0.298) | (-1.409, -0.407) |
|  | Thailand*** | -1.33 | -1.33 | (-2.236, -0.496) | (-2.095, -0.660) |
|  | Vietnam*** | -1.04 | -1.04 | (-1.873, -0.291) | (-1.716, -0.394) |
| Year*** |  | -0.058 | -0.058 | (-0.090, -0.025) | (-0.084, -0.030) |
| Rural population % (log scale)*** |  | -2.03 | -2.012 | (-2.985, -0.942) | -2.837, -1.145) |
| Age Lower |  | 0.008 | 0.008 | (-0.005, 0.019) | (-0.003, 0.018) |
| Population growth (%) (log scale) | 1 | -0.033 | -0.036 | (-0.801, 0.661) | (-0.650 , 0.535) |
| Vaccination* | 0 (Ref) | -0.39 | -0.39 | (-0.846, 0.055) | (-0.776, -0.010) |

**S2 Table B. Sensitivity analysis: Table of odds ratio of predictors that were used in the regression to fit Japanese encephalitis case-fatality ratio with complete information on age for the inference model without using data from India.** Mean, Mean (Bootstrap), and 95% confidence interval (CI) of the odds ratio were presented. *** indicates that odds ratio of the factor is different from 1 with a significance level of 95%. Ref stands for reference category.

| **Predictors** | | Mean | Mean  (boot) | **95% CI**  **(Bootstrap)** | **90% CI**  **(Bootstrap)** |
| --- | --- | --- | --- | --- | --- |
| **Country** | Japan*** | -2.75 | -2.76 | (-4.509, -1.089) | (-4.182, -1.312) |
|  | South Korea*** | -0.72 | -0.723 | (-1.824 , 0.388) | (-1.638, 0.207) |
|  | Nepal* | 0.83 | 0.829 | (-0.072 , 1.751) | (0.074, 1.614) |
|  | Other countries and areas (Bangladesh, Cambodia, Hong Kong, Indonesia, Myanmar, Malaysia)* | 0.51 | 0.518 | (-0.366, 1.463) | (-0.240, 1.297) |
|  | Thailand | 0.12 | 0.118 | (-0.842, 0.929) | (-0.651, 0.810) |
|  | Vietnam | 0.34 | 0.346 | (-0.599, 1.330) | (-0.442, 1.142) |
| Year |  | -0.064 | -0.065 | (-0.103, -0.026) | (-0.096, -0.033) |
| Rural population % (log scale)*** |  | -2.31 | -2.316 | (-3.412, -1.152) | (-3.247, -1.348) |
| Age Lower*** |  | 0.004 | 0.004 | (-0.011, 0.020) | (-0.009, 0.017) |
| Population growth (%) (log scale) | 1 | -0.104 | -0.110 | (-0.808 , 0.576) | (-0.679, 0.443) |
| Vaccination*** | 0 (Ref) | -0.705 | -0.714 | (-1.378, -0.068) | (-1.263, -0.187) |
