# Supplementary material for: Estimates of Japanese Encephalitis mortality and morbidity: A systematic review and modeling analysis
Source: PLoS Negl Trop Dis. 2022 May 25;16(5):e0010361. doi: 10.1371/journal.pntd.0010361 (PMC9173604; doi:10.1371/journal.pntd.0010361)
Supplement: S3 Table — Country-specific and year-stratified JE CFRs and their associated 95% CI were presented. (DOCX) [file pntd.0010361.s006.docx]

**S3. Table of predicted and projected JE case-fatality ratio (CFR) estimated by the stacking model without including year as predictor fitted on JE records with clear case definition for 20 out of 24 JE endemic countries from 1961 to 2030.** Country-specific and year-stratified JE CFRs and their associated 95% CI were presented.

| JE endemic countries | Case-fatality ratio (95% CI) | | | |
| --- | --- | --- | --- | --- |
|  | Predicted | | | Projected |
|  | 1961-1979 | 1980-1999 | 2000-2018 | 2019-2030 |
| Overall | 28 (22, 33) | 25 (20, 30) | 18 (14, 23) | 14 (12, 17) |
| Bangladesh | 30 (13, 45) | 29 (15, 44) | 25 (13, 40) | 24 (13, 44) |
| Brunei | 27 (11, 43) | 25 (12, 42) | 18 (8, 34) | 15 (8, 25) |
| China | 20 (5, 37) | 20 (6, 37) | 15 (4, 32) | 7 (3, 16) |
| Guam | N/A | N/A | N/A | 20 (14, 29) |
| Indonesia | 29 (18, 41) | 26 (13, 42) | 22 (8, 37) | 8 (5, 16) |
| India | 28 (10, 43) | 28 (10, 44) | 21 (9, 36) | 16 (11, 24) |
| Japan | 32 (16, 47) | 23 (10, 38) | 17 (7, 33) | 11 (5, 19) |
| Cambodia | N/A | 24 (13, 38) | 11 (3, 21) | 8 (3, 18) |
| Korea | 32 (22, 45) | 33 (21, 46) | 21 (10, 38) | 12 (6, 21) |
| Lao PDR | N/A | 27 (15, 39) | 19 (9, 28) | 9 (4, 18) |
| Sri Lanka | 20 (13, 28) | 12 (6, 18) | 8 (3, 18) | 10 (5, 19) |
| Myanmar | N/A | N/A | 17 (9, 28) | 7 (4, 19) |
| Malaysia | 26 (15, 41) | 26 (14, 44) | 19 (9, 37) | 9 (4, 19) |
| Nepal | 23 (10, 37) | 21 (12, 33) | 8 (2, 16) | 9 (4, 20) |
| Pakistan | 36 (21, 47) | 34 (18, 47) | 32 (17, 47) | 28 (19, 38) |
| Philippines | 35 (22, 46) | 33 (20, 46) | 27 (15, 40) | 24 (17, 32) |
| Papua New Guinea | 22 (13, 32) | 22 (13, 32) | 18 (9, 28) | 18 (10, 29) |
| Thailand | 29 (18, 39) | 19 (11, 30) | 11 (5, 22) | 7 (4, 16) |
| Timor-Leste | N/A | N/A | 23 (14, 37) | 20 (12, 29) |
| Vietnam | N/A | 18 (11, 28) | 16 (9, 27) | 19 (13, 27) |
