# Supplementary material for: Estimates of Japanese Encephalitis mortality and morbidity: A systematic review and modeling analysis
Source: PLoS Negl Trop Dis. 2022 May 25;16(5):e0010361. doi: 10.1371/journal.pntd.0010361 (PMC9173604; doi:10.1371/journal.pntd.0010361)
Supplement: S4 Table — In each bootstrapped dataset we computed binomial log likelihood for each model. For each model summary statistics of the distribution of log likelihood was presented. Abbreviations: LASSO: Least Absolute Shrinkage and Selection Operator; PCR: Principal Component Regression; GBM: Gradient Boosting Machine; NN: Neural Network; MLR: Multiple Linear Regression. (DOCX) [file pntd.0010361.s007.docx]

**S4 Table. Table of binomial log likelihood calculated from 6 models with year as predictor in 2000 bootstrapped datasets sampled from the dataset collated from systematic review.** In each bootstrapped dataset we computed binomial log likelihood for each model. For each model summary statistics of the distribution of log likelihood was presented. Abbreviations: LASSO: Least Absolute Shrinkage and Selection Operator; PCR: Principal Component Regression; GBM: Gradient Boosting Machine; NN: Neural Network; MLR: Multiple Linear Regression.

| **Model** | With year | |
| --- | --- | --- |
|  | Median | 95% CI |
| **LASSO** | -10921 | (-10259803, -7260) |
| **PCR** | -66136 | (-273867, -9378) |
| **GBM** | -23159 | (-50092, -10971) |
| **NN** | -128928 | (-2109318, -24439) |
| **Stacking (MLR)** | -23988 | (-55489, -11238) |
| **Stacking (Weighting algorithm)** | -19617 | (-73664, -8853) |
