# Supplementary material for: Estimates of Japanese Encephalitis mortality and morbidity: A systematic review and modeling analysis
Source: PLoS Negl Trop Dis. 2022 May 25;16(5):e0010361. doi: 10.1371/journal.pntd.0010361 (PMC9173604; doi:10.1371/journal.pntd.0010361)
Supplement: S5 Table — Country-specific and year-stratified JE CFRs and their associated 95% CI were presented. (DOCX) [file pntd.0010361.s008.docx]

**S5. Table of predicted and projected JE case-fatality ratio (CFR) estimated by LASSO fitted on JE records with clear case definition for 20 out of 24 JE endemic countries from 1961 to 2030.** Country-specific and year-stratified JE CFRs and their associated 95% CI were presented.

| JE endemic countries | Case-fatality ratio (95% CI) | | | |
| --- | --- | --- | --- | --- |
|  | Predicted | | | Projected |
|  | 1961-1979 | 1980-1999 | 2000-2018 | 2019-2030 |
| Overall | 30 (19, 44) | 25 (16, 41) | 16 (9, 29) | 12 (6, 24) |
| Bangladesh | 33 (0, 99) | 27 (0, 89) | 8 (0, 24) | 2 (0, 9) |
| Brunei | 71 (17, 100) | 53 (4, 100) | 31 (0, 100) | 32 (3, 93) |
| China | 24 (0, 94) | 20 (0, 100) | 24 (0, 100) | 3 (2, 5) |
| Guam | N/A | N/A | N/A | 8 (4, 15) |
| Indonesia | 26 (0, 63) | 18 (0, 60) | 18 (0, 97) | 14 (3, 44) |
| India | 28 (10, 43) | 28 (10, 44) | 21 (9, 36) | 8 (1, 25) |
| Japan | 41 (8, 94) | 23 (1, 80) | 19 (0, 75) | 14 (3, 44) |
| Cambodia | N/A | 32 (5, 74) | 10 (2, 24) | 8 (1, 25) |
| Korea | 22 (8, 42) | 11 (2, 39) | 6 (0, 20) | 2 (0, 12) |
| Lao PDR | N/A | 25 (6, 47) | 16 (2, 69) | 27 (6, 72) |
| Sri Lanka | 13 (2, 26) | 5 (0, 11) | 3 (0, 8) | 1 (0, 4) |
| Myanmar | N/A | N/A | 17 (2, 74) | 37 (4, 94) |
| Malaysia | 27 (0, 98) | 30 (5, 100) | 21 (1, 99) | 12 (0, 61) |
| Nepal | 16 (0, 52) | 23 (2, 46) | 7 (0, 18) | 14 (5, 33) |
| Pakistan | 28 (1, 62) | 29 (10, 78) | 21 (2, 51) | 5 (0, 15) |
| Philippines | 45 (11, 93) | 35 (8, 84) | 15 (1, 43) | 3 (0, 9) |
| Papua New Guinea | 18 (1, 47) | 30 (4, 83) | 25 (2, 86) | 41 (7, 94) |
| Thailand | 38 (14, 78) | 13 (4, 40) | 5 (2, 11) | 2 (0, 5) |
| Timor-Leste | N/A | N/A | 22 (3, 75) | 24 (6, 60) |
| Vietnam | N/A | 9 (2, 20) | 3 (0, 6) | 1 (0, 3) |
