# Supplementary material for: Estimates of Japanese Encephalitis mortality and morbidity: A systematic review and modeling analysis
Source: PLoS Negl Trop Dis. 2022 May 25;16(5):e0010361. doi: 10.1371/journal.pntd.0010361 (PMC9173604; doi:10.1371/journal.pntd.0010361)
Supplement: S6 Table — (DOCX) [file pntd.0010361.s009.docx]

**S6 Table. Table of dropout analysis**

| **Reference** | **Follow-up** | **Age** | **Country** | **Year** | **Death (%)** | **Sequelae**  **(%)** | **Complete Recovery (%)** | **#. of studied JE patients** | **#.of dropout or lost of follow-up (proportion)** |
| --- | --- | --- | --- | --- | --- | --- | --- | --- | --- |
| [1] | Discharge | 3-12 | India | 1998-1998 | 24 | 67 | 9 | 33 | 6 |
|  | After one year |  |  | 1998-1998 | 0 | 41 | 59 | 22 | 15% |
| [2] | Discharge | 2-15 | China | 2005-2006 | 0 | 56 | 44 | 32 | 22 refused, 6 without contact information (34%) |
|  |  | 16-54 |  |  | 0 | 74 | 26 | 23 |  |
|  | 1-2 year | 2-15 |  |  | 0 | 41 | 59 | 32 |  |
|  |  | 16-65 |  |  | 0 | 48 | 52 | 23 |  |
| [3] | Gained consciousness. | 1-15 | Vietnam | 1992-1992 | 0 | 96 | 4 | 50 | 0 |
|  | Half month after discharge |  |  |  | 0 | 68 | 32 | 50 |  |
|  | 1.5 month after discharge |  |  |  | 0 | 58 | 42 | 50 |  |
| [4] | 1-2 months | 0-55 | Bangladesh | 2003-2005 | 13 | 80 | 7 | 15 | 0 |
| [5] | 3-5 years | 0-99 | India | 1994-1996 | 0 | 33 | 60 | 15 | 0 |
| [6] | 5 years after discharge | 15-78 | India | 1978-1989 | 0 | 3 | 97 | 544 | 144/688 (21%) |
| [7] | 6-27 years | 0-99 | China | 1973-1994 | 0 | 22 | 78 | 78 | 61(lost to follow up), 14 refused  Total 160 (intended to follow up) (52%) |

2: The main reasons for refusal included that some cases were living in distant mountain areas and were unable to come to the CDC, were too

weak to go outside, or were unwilling to accept medical examination

5: Letters were sent to 109 surviving patients requesting follow-up. 15 patients were reviewed in 1999 after an interval of 3 to 5 years since their initial infection. Two neurologists examined 12 of these 15 patients; the remaining three patients informed us through letters that they were in good health

6: 144/688 (21%) (Table 4) and Fig. 2. Because of socioeconomic and travel problems, and as many of them had regained normal livelihood, they had possibly decided not to attend.

7: we were able to follow only 48% of the discharged patients. Patients lost from the follow up because they had changed their addresses during the recent 20 years.

Liverpool Outcome Score

| Reference | Follow-up | Age | Country | Year | Death (%) | Sequelae (%) | Severe (%) | Moderate or Minor (%) | Complete Recovery (%) | #. of studied JE patients | #.of dropout/lost to follow up |
| --- | --- | --- | --- | --- | --- | --- | --- | --- | --- | --- | --- |
| [8] | Discharge | 1-11 | Nepal | 2009-2009 | 9 | 82 | 64 | 18 | 9 | 11 | 2 (15%) |
|  | 3-6 months |  |  |  | 0 | 67 | 33 | 35 | 33 | 10 |  |
| [9] | Discharge | 0-12 | Malaysia | 1997-2005 | 9 | 60 | 31 | 29 | 41 | 108 | 22/108 (20%) |
|  | 1-115 months |  |  |  | 2 | 56 | 13 | 43 | 42 | 86 |  |
| [10] | 3 months – 2 years | 2-73 | Vietnam | 2006-2008 | 0 | 58 | 8 | 50 | 42 | 26 | 14% |
|  | 6 months – 2 years | 1-15 | Cambodia | 2006-2007 | 0 | 95 | 11 | 84 | 5 | 38 | 5 (can not be contacted)  12% |
| [11] | 4– 26 months | 1-14 | Indonesia | 2005-2006 | 0 | 67 | 33 | 35 | 33 | 49 | 7 12.5% |
| [12] | 5-12 months | 0-14 | Nepal | 2011-2011 | 14 | 59 | 18 | 41 | 27 | 22 | 23 cannot be contacted, 1 refused  (35%) |
| [13] | 6-13 months | 0-18 | China | 2010-2010 | 0 | 40 | 8 | 32 | 60 | 79 | 21/108 (19%) |
| [14] | 38-88 months | 5-11 | Lao PDR | 2003-2013 | 20 | 56 | 4 | 51 | 24 | 45 | 33/123  (27%) |
|  |  | 18-33 |  |  | 11 | 46 | 3 | 43 | 44 | 35 |  |

9: However, those patients who were lost to follow-up were similar to those patients who were followed-up

11: five families had relocated, one village could not be visited because of security issues, and one parent refused consent.

13: Were the children too severe to make the trip or fully recovered to ignore the call

14: can find patient’s village, no contact details

Modified Rankin Scale

| Reference | Follow-up | Age | Country | Year | Death (%) | Sequelae (%) | Severe (%) | Moderate or minor (%) | Complete Recovery (%) | #. of studied JE patients | #. Of dropout or lost to follow-up |
| --- | --- | --- | --- | --- | --- | --- | --- | --- | --- | --- | --- |
| [15] | Discharge | 18-39 | China | 2011-2018 | 5 | 95 | 44 | 51 | 0 | 39 | 0 |
|  |  | 50-71 |  |  | 36 | 64 | 64 | 0 | 0 | 11 |  |
|  | 3-36 months | 18-39 |  |  | 0 | 84 | 11 | 73 | 16 | 37 |  |
|  |  | 50-71 |  |  | 14 | 86 | 57 | 29 | 0 | 7 |  |
| [16] | 1 month | 2-10 | China | 2013-2013 | 20 | 80 | 73 | 7 | 0 | 15 | 0 |
|  | 3 months |  |  |  | 0 | 82 | 50 | 42 | 8 | 12 |  |
|  | 6 months |  |  |  | 0 | 66 | 33 | 33 | 33 | 12 |  |
|  | 12 months |  |  |  | 0 | 50 | 25 | 25 | 50 | 12 |  |
| [17] | 6 months | 18-99 | China | 2017-2018 | 0 | 71 | 43 | 29 | 29 | 7 | 0 |

Good/partial/poor

| Reference | Follow-up | Age | Country | Year | Death (%) | Sequelae (%) Partial and poor | Complete Recovery (%) | #. of studied JE patients | #. of Dropout or lost to follow-up |
| --- | --- | --- | --- | --- | --- | --- | --- | --- | --- |
| [18] | Discharge | 2-54 | NA | 1994-1995 | 12 | 82 | 6 | 17 | 0 |
|  | 3 months |  |  |  | 0 | 100 (79 poor, 21 partial) | 0 | 14 |  |
| [19] | 3 months | 7-70 | India | 1998-1998 | 21 | 21 | 58 | 14 | 0 |
| [20] | 3 months | 2-54 | India | NA | 0 | 63 | 37 | 19 | 3 (14%) |
| [21] | 3 months | 7-19 | India | 1992-1997 | 0 | 100 | 0 | 5 | 0 |
|  | 1 year |  |  |  | 0 | 75 | 25 | 4 |  |
| [22] | 3 months | 2-65 | India | 1992-1999 | 0 | 52 | 48 | 54 | 3 |
| [23] | 3 months | 2-64 | India^^[[1]](#footnote-1)^^ | 1992-1998 | 6 | 21 | 63 | 16 | 0 |
|  |  |  |  |  | 0 | 79 | 21 |  |  |
| [24] | 6 months | 0-15 | India and Nepal | 1993-2003 | 0 | 73 | 27 | 30 | 0 |
|  |  | 15-59 |  |  | 16 | 41 | 41 | 37 |  |
| [25] | 6 months | 0-12 | India^^[[2]](#footnote-2)^^ | 1992-1996 | 0 | 73 | 27 | 11 | 0 |
|  | 6 months | 13-54 |  |  | 24 | 53 | 24 | 17 |  |
| [26] | 12 months | 1-78 | India | 2004-2014 | 1 | 49 | 50 | 73 | 5 6% |

| Reference | Follow-up | Age | Country | Year | Death (%) | Sequelae (%)  (Severe/Moderate or Minor) | Complete Recovery (%) | #. of studied JE patients | #. of Dropout or lost to follow-up |
| --- | --- | --- | --- | --- | --- | --- | --- | --- | --- |
| Whitely | | | | | | | | |  |
| [27] | Discharge | 0-14 | India | 2009-2011 | 20 | 44(12/32)^^[[3]](#footnote-3)^^ | 37 | 41 | 12 (27%) |
|  |  | 15-99 |  |  | 15 | 59(7/52) | 26 | 97 | 31 (30%) |
| Severe/Moderate/Minor | | | | | | | | |  |
| [28] | 1-2 months | 8-32 | India | NA | 0 | 50(17/33) | 50 | 6 | 0 |

27: Adults: 104 patients, 7 leave against medical advice, 24 lost to follow-up, Children, 3 leave against medical advices, 9 lost to follow-up

References

[1] Baruah HC, Biswas D, Patgiri D, et al. Clinical outcome and neurological sequelae in serologically confirmed cases of Japanese encephalitis patients in Assam, India. *Indian Pediatr*.

[2] Yin Z, Wang X, Li L, et al. Neurological sequelae of hospitalized Japanese encephalitis cases in Gansu province, China. *Am J Trop Med Hyg* 2015; 92: 1125–1129.

[3] Huy B V., Tu HC, Luan T V., et al. Early mental and neurological sequelae after Japanese B encephalitis. *Southeast Asian J Trop Med Public Health* 1994; 25: 549–553.

[4] Hossain MJ, Gurley ES, Montgomery S, et al. Hospital-based surveillance for Japanese encephalitis at four sites in Bangladesh, 2003-2005. *Am J Trop Med Hyg* 2010; 82: 344–349.

[5] Murgod UA, Muthane UB, Ravi V, et al. Persistent movement disorders following Japanese encephalitis. *Neurology* 2001; 57: 2313–2315.

[6] Sarkari NBS, Thacker AK, Barthwal SP, et al. Japanese encephalitis (JE) part II: 14 Years’ follow-up of survivors. *J Neurol* 2012; 259: 58–69.

[7] Ding D, Hong Z, Zhao SJ, et al. Long-term disability from acute childhood Japanese encephalitis in Shanghai, China. *Am J Trop Med Hyg* 2007; 77: 528–533.

[8] Rayamajhi A, Nightingale S, Bhatta NK, et al. A preliminary randomized double blind placebo-controlled trial of intravenous immunoglobulin for Japanese encephalitis in Nepal. *PLoS One*; 10. Epub ahead of print 2015. DOI: 10.1371/journal.pone.0122608.

[9] Mong HO, Lewthwaite P, Boon FL, et al. The epidemiology, clinical features, and long-term prognosis of Japanese encephalitis in central Sarawak, Malaysia, 1997-2005. *Clin Infect Dis* 2008; 47: 458–468.

[10] Hills SL, Van cuong N, Touch S, et al. Disability from Japanese encephalitis in Cambodia and Viet Nam. *J Trop Pediatr*. Epub ahead of print 2011. DOI: 10.1093/tropej/fmp133.

[11] Maha MS, Moniaga VA, Hills SL, et al. Outcome and extent of disability following Japanese encephalitis in Indonesian children. *Int J Infect Dis*. Epub ahead of print 2009. DOI: 10.1016/j.ijid.2009.01.009.

[12] Griffiths MJ, Lemon J V., Rayamajhi A, et al. The Functional, Social and Economic Impact of Acute Encephalitis Syndrome in Nepal - a Longitudinal Follow-Up Study. *PLoS Negl Trop Dis* 2013; 7: 17–19.

[13] Ma J, Jiang L. Outcome of children with japanese encephalitis and predictors of outcome in southwestern China. *Trans R Soc Trop Med Hyg* 2013; 107: 660–665.

[14] Mayxay M, Douangdala P, Vilayhong C, et al. Outcome of Japanese encephalitis virus (JEV) infection in pediatric and adult patients at Mahosot Hospital, Vientiane, Lao PDR. *Am J Trop Med Hyg* 2021; 104: 567–575.

[15] Xiong W, Lu L, Xiao Y, et al. Mortality and disability due to japanese encephalitis in elderly adults: Evidence from an adult tertiary care center in west China. *Front Neurol* 2019; 10: 1–6.

[16] LI JW, GAO XY, WU Y, et al. A Centralized Report on Pediatric Japanese Encephalitis Cases from Beijing Children’s Hospital, 2013. *Biomedical and Environmental Sciences*. Epub ahead of print 2016. DOI: 10.3967/bes2016.121.

[17] WHO. Population-based age-stratified seroepidemiological investigation protocol for coronavirus 2019 (COVID-19) infection. 2020; 20.

[18] Misra UK, Kalita J. Movement disorders in japanese encephalitis. *J Neurol* 1997; 244: 299–303.

[19] Misra UK, Kalita J, Goel D, et al. Clinical, radiological and neurophysiological spectrum of JEV encephalitis and other non-specific encephalitis during post-monsoon period in India. *Neurol India* 2003; 51: 55–59.

[20] Kalita J, Misra UK. EEG in Japanese encephalitis: A clinico-radiological correlation. *Electroencephalogr Clin Neurophysiol* 1998; 106: 238–243.

[21] Kalita J, Misra UK. Markedly severe dystonia in Japanese encephalitis. *Mov Disord* 2000; 15: 1168–1172.

[22] Kalita J, Misra UK. Neurophysiological changes in Japanese encephalitis. *Neurol India* 2002; 50: 262–266.

[23] Misra UK, Kalita J. Prognosis of Japanese encephalitis patients with dystonia compared to those with parkinsonian features only. *Postgrad Med J* 2002; 78: 238–241.

[24] Kalita J, Misra UK, Pandey S, et al. A Comparison of Clinical and Radiological Findings in Adults and Children with Japanese Encephalitis. *Arch Neurol* 2003; 60: 1760–1764.

[25] Misra UK, Kalita J, Srivastava M. Prognosis of Japanese encephalitis: A multivariate analysis. *J Neurol Sci* 1998; 161: 143–147.

[26] Kalita J, Misra UK, Mani VE, et al. Can we differentiate between herpes simplex encephalitis and Japanese encephalitis? *J Neurol Sci* 2016; 366: 110–115.

[27] Basumatary LJ, Raja D, Bhuyan D, et al. Clinical and radiological spectrum of Japanese encephalitis. *J Neurol Sci* 2013; 325: 15–21.

[28] Pradhan S, Gupta RK, Singh MB, et al. Biphasic illness pattern due to early relapse in Japanese-B virus encephalitis. *J Neurol Sci* 2001; 183: 13–18.

1. Inferred. Country was not mentioned in the original paper. The patients with parkinsonian features only were classified into group I and those with additional dystonia or dyskinesia into group II [↑](#footnote-ref-1)
2. Inferred [↑](#footnote-ref-2)
3. Severe/moderate and minor [↑](#footnote-ref-3)
