# Supplementary material for: Estimates of Japanese Encephalitis mortality and morbidity: A systematic review and modeling analysis
Source: PLoS Negl Trop Dis. 2022 May 25;16(5):e0010361. doi: 10.1371/journal.pntd.0010361 (PMC9173604; doi:10.1371/journal.pntd.0010361)
Supplement: S7 Table — Mean CFR, Lower and Upper bounds of the 95% C.I. are presented. (DOCX) [file pntd.0010361.s010.docx]

**Table 7A: Sensitivity analysis: Table of predicted CFRs for all countries from 1961-1979, 1980-1999, 2000-2018 using model 6 when fitted without using data from China.**

| Country | Year | Mean | Lower | Upper |
| --- | --- | --- | --- | --- |
| Bangladesh | 1961-1979 | 0.37439333 | 0.1627155 | 0.57639276 |
| Bangladesh | 1980-1999 | 0.32527154 | 0.13839608 | 0.52112511 |
| Bangladesh | 2020-2018 | 0.24705989 | 0.10278349 | 0.41854535 |
| Brunei Darussalam | 1961-1979 | 0.25706732 | 0.11597026 | 0.42973131 |
| Brunei Darussalam | 1980-1999 | 0.19875871 | 0.08891941 | 0.35549666 |
| Brunei Darussalam | 2020-2018 | 0.16276661 | 0.08135526 | 0.29306231 |
| China | 1961-1979 | 0.21037221 | 0.03919315 | 0.47569376 |
| China | 1980-1999 | 0.183444 | 0.03821178 | 0.45684556 |
| China | 2020-2018 | 0.16066732 | 0.02256963 | 0.70171716 |
| Indonesia | 1961-1979 | 0.32601358 | 0.15197099 | 0.48901395 |
| Indonesia | 1980-1999 | 0.23642741 | 0.07059179 | 0.44366282 |
| Indonesia | 2020-2018 | 0.17290571 | 0.04124725 | 0.39220924 |
| India | 1961-1979 | 0.29894713 | 0.05979768 | 0.52788997 |
| India | 1980-1999 | 0.26976359 | 0.04953613 | 0.51552538 |
| India | 2020-2018 | 0.18664934 | 0.07108064 | 0.36778397 |
| Japan | 1961-1979 | 0.27757214 | 0.07240815 | 0.50445608 |
| Japan | 1980-1999 | 0.21017311 | 0.06123421 | 0.41740323 |
| Japan | 2020-2018 | 0.15317127 | 0.02851329 | 0.3770146 |
| Cambodia | 1961-1979 | NaN | NA | NA |
| Cambodia | 1980-1999 | 0.30077646 | 0.19077794 | 0.42906664 |
| Cambodia | 2020-2018 | 0.11117787 | 0.03251888 | 0.25692595 |
| Korea | 1961-1979 | 0.29699275 | 0.19036138 | 0.42454204 |
| Korea | 1980-1999 | 0.28496891 | 0.09465413 | 0.44694524 |
| Korea | 2020-2018 | 0.22650158 | 0.07413991 | 0.38815081 |
| Lao PDR | 1961-1979 | NaN | NA | NA |
| Lao PDR | 1980-1999 | 0.35666 | 0.20590117 | 0.50060706 |
| Lao PDR | 2020-2018 | 0.20205092 | 0.06696553 | 0.32962833 |
| Sri Lanka | 1961-1979 | 0.17318339 | 0.09701984 | 0.26002196 |
| Sri Lanka | 1980-1999 | 0.08860669 | 0.0457633 | 0.16261271 |
| Sri Lanka | 2020-2018 | 0.06949714 | 0.02819843 | 0.17305051 |
| Myanmar | 1961-1979 | NaN | NA | NA |
| Myanmar | 1980-1999 | NaN | NA | NA |
| Myanmar | 2020-2018 | 0.24862233 | 0.1100679 | 0.42585677 |
| Malaysia | 1961-1979 | 0.22813226 | 0.12660234 | 0.36705143 |
| Malaysia | 1980-1999 | 0.20782655 | 0.11934797 | 0.3410683 |
| Malaysia | 2020-2018 | 0.18386155 | 0.08766682 | 0.3243164 |
| Nepal | 1961-1979 | 0.31375637 | 0.15846035 | 0.46368029 |
| Nepal | 1980-1999 | 0.27866505 | 0.1392479 | 0.43385251 |
| Nepal | 2020-2018 | 0.08060235 | 0.02265772 | 0.1667639 |
| Pakistan | 1961-1979 | 0.3828813 | 0.23004694 | 0.50930978 |
| Pakistan | 1980-1999 | 0.32473842 | 0.13873368 | 0.5029378 |
| Pakistan | 2020-2018 | 0.24876197 | 0.08892539 | 0.44538855 |
| Philippines | 1961-1979 | 0.31249729 | 0.19607423 | 0.43057989 |
| Philippines | 1980-1999 | 0.23632859 | 0.0891578 | 0.38382548 |
| Philippines | 2020-2018 | 0.21099275 | 0.07684634 | 0.36610209 |
| Papua New Guinea | 1961-1979 | 0.31934946 | 0.18806577 | 0.46170478 |
| Papua New Guinea | 1980-1999 | 0.22618428 | 0.12979099 | 0.34595798 |
| Papua New Guinea | 2020-2018 | 0.16889383 | 0.08980536 | 0.27537816 |
| Thailand | 1961-1979 | 0.29790273 | 0.16310235 | 0.45257693 |
| Thailand | 1980-1999 | 0.17419566 | 0.10143841 | 0.27737265 |
| Thailand | 2020-2018 | 0.12044479 | 0.04649628 | 0.26756645 |
| Timor-Leste | 1961-1979 | NaN | NA | NA |
| Timor-Leste | 1980-1999 | NaN | NA | NA |
| Timor-Leste | 2020-2018 | 0.2107357 | 0.11603364 | 0.33681015 |
| Vietnam | 1961-1979 | NaN | NA | NA |
| Vietnam | 1980-1999 | 0.12289587 | 0.07470629 | 0.19342366 |
| Vietnam | 2020-2018 | 0.14371081 | 0.064494 | 0.28349966 |

**Table 7B: Sensitivity analysis: Table of predicted CFRs for all countries from 1961-1979, 1980-1999, 2000-2018 using model 6 when fitted without using data from India.**

| Country | Year | Mean | Lower | Upper |
| --- | --- | --- | --- | --- |
| Bangladesh | 1961-1979 | 0.37439333 | 0.1627155 | 0.57639276 |
| Bangladesh | 1980-1999 | 0.32527154 | 0.13839608 | 0.52112511 |
| Bangladesh | 2020-2018 | 0.24705989 | 0.10278349 | 0.41854535 |
| Brunei Darussalam | 1961-1979 | 0.25706732 | 0.11597026 | 0.42973131 |
| Brunei Darussalam | 1980-1999 | 0.19875871 | 0.08891941 | 0.35549666 |
| Brunei Darussalam | 2020-2018 | 0.16276661 | 0.08135526 | 0.29306231 |
| China | 1961-1979 | 0.21037221 | 0.03919315 | 0.47569376 |
| China | 1980-1999 | 0.183444 | 0.03821178 | 0.45684556 |
| China | 2020-2018 | 0.16066732 | 0.02256963 | 0.70171716 |
| Indonesia | 1961-1979 | 0.32601358 | 0.15197099 | 0.48901395 |
| Indonesia | 1980-1999 | 0.23642741 | 0.07059179 | 0.44366282 |
| Indonesia | 2020-2018 | 0.17290571 | 0.04124725 | 0.39220924 |
| India | 1961-1979 | 0.29894713 | 0.05979768 | 0.52788997 |
| India | 1980-1999 | 0.26976359 | 0.04953613 | 0.51552538 |
| India | 2020-2018 | 0.18664934 | 0.07108064 | 0.36778397 |
| Japan | 1961-1979 | 0.27757214 | 0.07240815 | 0.50445608 |
| Japan | 1980-1999 | 0.21017311 | 0.06123421 | 0.41740323 |
| Japan | 2020-2018 | 0.15317127 | 0.02851329 | 0.3770146 |
| Cambodia | 1961-1979 | NaN | NA | NA |
| Cambodia | 1980-1999 | 0.30077646 | 0.19077794 | 0.42906664 |
| Cambodia | 2020-2018 | 0.11117787 | 0.03251888 | 0.25692595 |
| Korea | 1961-1979 | 0.29699275 | 0.19036138 | 0.42454204 |
| Korea | 1980-1999 | 0.28496891 | 0.09465413 | 0.44694524 |
| Korea | 2020-2018 | 0.22650158 | 0.07413991 | 0.38815081 |
| Lao PDR | 1961-1979 | NaN | NA | NA |
| Lao PDR | 1980-1999 | 0.35666 | 0.20590117 | 0.50060706 |
| Lao PDR | 2020-2018 | 0.20205092 | 0.06696553 | 0.32962833 |
| Sri Lanka | 1961-1979 | 0.17318339 | 0.09701984 | 0.26002196 |
| Sri Lanka | 1980-1999 | 0.08860669 | 0.0457633 | 0.16261271 |
| Sri Lanka | 2020-2018 | 0.06949714 | 0.02819843 | 0.17305051 |
| Myanmar | 1961-1979 | NaN | NA | NA |
| Myanmar | 1980-1999 | NaN | NA | NA |
| Myanmar | 2020-2018 | 0.24862233 | 0.1100679 | 0.42585677 |
| Malaysia | 1961-1979 | 0.22813226 | 0.12660234 | 0.36705143 |
| Malaysia | 1980-1999 | 0.20782655 | 0.11934797 | 0.3410683 |
| Malaysia | 2020-2018 | 0.18386155 | 0.08766682 | 0.3243164 |
| Nepal | 1961-1979 | 0.31375637 | 0.15846035 | 0.46368029 |
| Nepal | 1980-1999 | 0.27866505 | 0.1392479 | 0.43385251 |
| Nepal | 2020-2018 | 0.08060235 | 0.02265772 | 0.1667639 |
| Pakistan | 1961-1979 | 0.3828813 | 0.23004694 | 0.50930978 |
| Pakistan | 1980-1999 | 0.32473842 | 0.13873368 | 0.5029378 |
| Pakistan | 2020-2018 | 0.24876197 | 0.08892539 | 0.44538855 |
| Philippines | 1961-1979 | 0.31249729 | 0.19607423 | 0.43057989 |
| Philippines | 1980-1999 | 0.23632859 | 0.0891578 | 0.38382548 |
| Philippines | 2020-2018 | 0.21099275 | 0.07684634 | 0.36610209 |
| Papua New Guinea | 1961-1979 | 0.31934946 | 0.18806577 | 0.46170478 |
| Papua New Guinea | 1980-1999 | 0.22618428 | 0.12979099 | 0.34595798 |
| Papua New Guinea | 2020-2018 | 0.16889383 | 0.08980536 | 0.27537816 |
| Thailand | 1961-1979 | 0.29790273 | 0.16310235 | 0.45257693 |
| Thailand | 1980-1999 | 0.17419566 | 0.10143841 | 0.27737265 |
| Thailand | 2020-2018 | 0.12044479 | 0.04649628 | 0.26756645 |
| Timor-Leste | 1961-1979 | NaN | NA | NA |
| Timor-Leste | 1980-1999 | NaN | NA | NA |
| Timor-Leste | 2020-2018 | 0.2107357 | 0.11603364 | 0.33681015 |
| Vietnam | 1961-1979 | NaN | NA | NA |
| Vietnam | 1980-1999 | 0.12289587 | 0.07470629 | 0.19342366 |
| Vietnam | 2020-2018 | 0.14371081 | 0.064494 | 0.28349966 |

**Table 7C: Sensitivity analysis: Table of projected CFRs for all countries from 2018 onwards projected without using data from China.**

| Country | Year | Mean | Upper | Lower |
| --- | --- | --- | --- | --- |
| Bangladesh | 2020-2018 | 0.25160394 | 0.11307019 | 0.44415684 |
| Brunei Darussalam | 2020-2018 | 0.13423311 | 0.06150005 | 0.23567782 |
| China | 2020-2018 | 0.05517637 | 0.02632577 | 0.17406621 |
| Guam | 2020-2018 | 0.2177509 | 0.14389258 | 0.33190383 |
| Indonesia | 2020-2018 | 0.0551448 | 0.02926344 | 0.13589792 |
| India | 2020-2018 | 0.13184296 | 0.06335666 | 0.23119896 |
| Japan | 2020-2018 | 0.11718992 | 0.05961851 | 0.21289049 |
| Cambodia | 2020-2018 | 0.0628606 | 0.02798034 | 0.16350739 |
| Korea | 2020-2018 | 0.16048842 | 0.08718565 | 0.27438812 |
| Lao PDR | 2020-2018 | 0.06693995 | 0.03067592 | 0.16351038 |
| Sri Lanka | 2020-2018 | 0.08500341 | 0.03618302 | 0.19302018 |
| Myanmar | 2020-2018 | 0.08148305 | 0.02724 | 0.17914392 |
| Malaysia | 2020-2018 | 0.06879907 | 0.02826118 | 0.17210268 |
| Nepal | 2020-2018 | 0.07998908 | 0.03512652 | 0.18641883 |
| Pakistan | 2020-2018 | 0.22907484 | 0.1392172 | 0.34213446 |
| Philippines | 2020-2018 | 0.20799923 | 0.12037781 | 0.30849637 |
| Papua New Guinea | 2020-2018 | 0.14712282 | 0.08359505 | 0.2515378 |
| Thailand | 2020-2018 | 0.04639093 | 0.02218002 | 0.16197749 |
| Timor-Leste | 2020-2018 | 0.14840868 | 0.09052626 | 0.23711443 |
| Vietnam | 2020-2018 | 0.19199771 | 0.10646094 | 0.30172393 |

**Table 7D: Sensitivity analysis: Table of projected CFRs for all countries from 2018 onwards projected without using data from India.**

| Country | Year | Mean | Upper | Lower |
| --- | --- | --- | --- | --- |
| Bangladesh | 2020-2018 | 0.25160394 | 0.11307019 | 0.44415684 |
| Brunei Darussalam | 2020-2018 | 0.13423311 | 0.06150005 | 0.23567782 |
| China | 2020-2018 | 0.05517637 | 0.02632577 | 0.17406621 |
| Guam | 2020-2018 | 0.2177509 | 0.14389258 | 0.33190383 |
| Indonesia | 2020-2018 | 0.0551448 | 0.02926344 | 0.13589792 |
| India | 2020-2018 | 0.13184296 | 0.06335666 | 0.23119896 |
| Japan | 2020-2018 | 0.11718992 | 0.05961851 | 0.21289049 |
| Cambodia | 2020-2018 | 0.0628606 | 0.02798034 | 0.16350739 |
| Korea | 2020-2018 | 0.16048842 | 0.08718565 | 0.27438812 |
| Lao PDR | 2020-2018 | 0.06693995 | 0.03067592 | 0.16351038 |
| Sri Lanka | 2020-2018 | 0.08500341 | 0.03618302 | 0.19302018 |
| Myanmar | 2020-2018 | 0.08148305 | 0.02724 | 0.17914392 |
| Malaysia | 2020-2018 | 0.06879907 | 0.02826118 | 0.17210268 |
| Nepal | 2020-2018 | 0.07998908 | 0.03512652 | 0.18641883 |
| Pakistan | 2020-2018 | 0.22907484 | 0.1392172 | 0.34213446 |
| Philippines | 2020-2018 | 0.20799923 | 0.12037781 | 0.30849637 |
| Papua New Guinea | 2020-2018 | 0.14712282 | 0.08359505 | 0.2515378 |
| Thailand | 2020-2018 | 0.04639093 | 0.02218002 | 0.16197749 |
| Timor-Leste | 2020-2018 | 0.14840868 | 0.09052626 | 0.23711443 |
| Vietnam | 2020-2018 | 0.19199771 | 0.10646094 | 0.30172393 |
