# Supplementary material for: Estimates of Japanese Encephalitis mortality and morbidity: A systematic review and modeling analysis
Source: PLoS Negl Trop Dis. 2022 May 25;16(5):e0010361. doi: 10.1371/journal.pntd.0010361 (PMC9173604; doi:10.1371/journal.pntd.0010361)

# Supplementary Information: Estimates of Japanese Encephalitis mortality and morbidity: a systematic review and modelling analysis

# Data collection

## Estimation dataset

We conducted a systematic review to collect Japanese encephalitis (JE) case-fatality data. We extracted year, length of study, age, number of JE cases and deaths and we used them to compute case-fatality ratio (CFR). We also gathered JE diagnostic method, type of surveillance system that collected and reported JE cases, the geographical location of the study, and information on whether the data were collected during an outbreak. We collected JE national or regional vaccination information from World Health Organization surveillance data(1). In total we had 469 age and time stratified JE case-fatality records from 82 different studies.

We collated socioeconomic feature from the World Bank Open Data(2). Country-level variables: population density(3), population growth(4), GDP growth rate(5), GDP per capita(6), under-5 mortality(7), rural population (% of total population)(8), and urban population(9) were collected because these variables were available from 1961 and 2018 and had lower than 10% missing values. In total we had 7 socioeconomic features. With features collated from the systematic review, we had the estimation dataset X_nxp_ with n=469 and p = 16 and response variable JE case-fatality ratio denoted as y with length of 469.

## Prediction dataset

We collated the 7 socioeconomic features from 1961 to 2018 from the World Bank Data for 19 JE endemic areas. We exclude Guam because the variable under-5 mortality was not available for this country. As GDP annual growth rate was not available for North Korea, we did not do JE prediction for this country as well. We excluded Taiwan and Northern Marina Islands due to the unavailability of socioeconomic features. Australia, Russia, and Singapore were excluded from the analysis because we believed that no JE infection occurred in these countries. We fixed the JE diagnostic method as WHO case definition: JE IgM antibody in CSF or serum as confirmed by MAC-ELISA on patients with acute encephalitis syndrome. We fixed the type of surveillance scale as national surveillance.

## Projection dataset

The projected values for population density from 2019 to 2030 by year and population growth rate and under-5 mortality from 2019-2030 by 5 years were available at the World Population Prospect(10). Projected GDP annual growth rate by year was collected from Congressional Budget Office(11). The projected GDP per capital was assumed to grow 1% per year(12). Projection estimates were available for Guam.

# Statistical inference

To select variables that were critical to explain JE CFR, we used stepwise model selection with BIC as criteria. The response variable was the log odds of JE CFR collated from systematic review. In the full model, we included year, length of study, age lower, age upper, JE diagnostic method, surveillance type, geographical location of the study, outbreak, youth, vaccination, GDP per capita, GDP annual growth rate, under-5 mortality rate, population growth, population density, rural population (% of total population), and urban population (in million) as predictors (Detailed explanation can be found in S1 Table). We took log transformation for GDP per capita, population density, under-5 mortality, rural population (% of total population) to transfer their heavily tailed distribution into a more normal one. We benefited from this transformation as we were less likely to see many influential points when covariates had more normal-look distributions. Before conducting model selection, we conducted model diagnostic for the full-model.

**Fig A: Model Diagnostic for the Full Model**


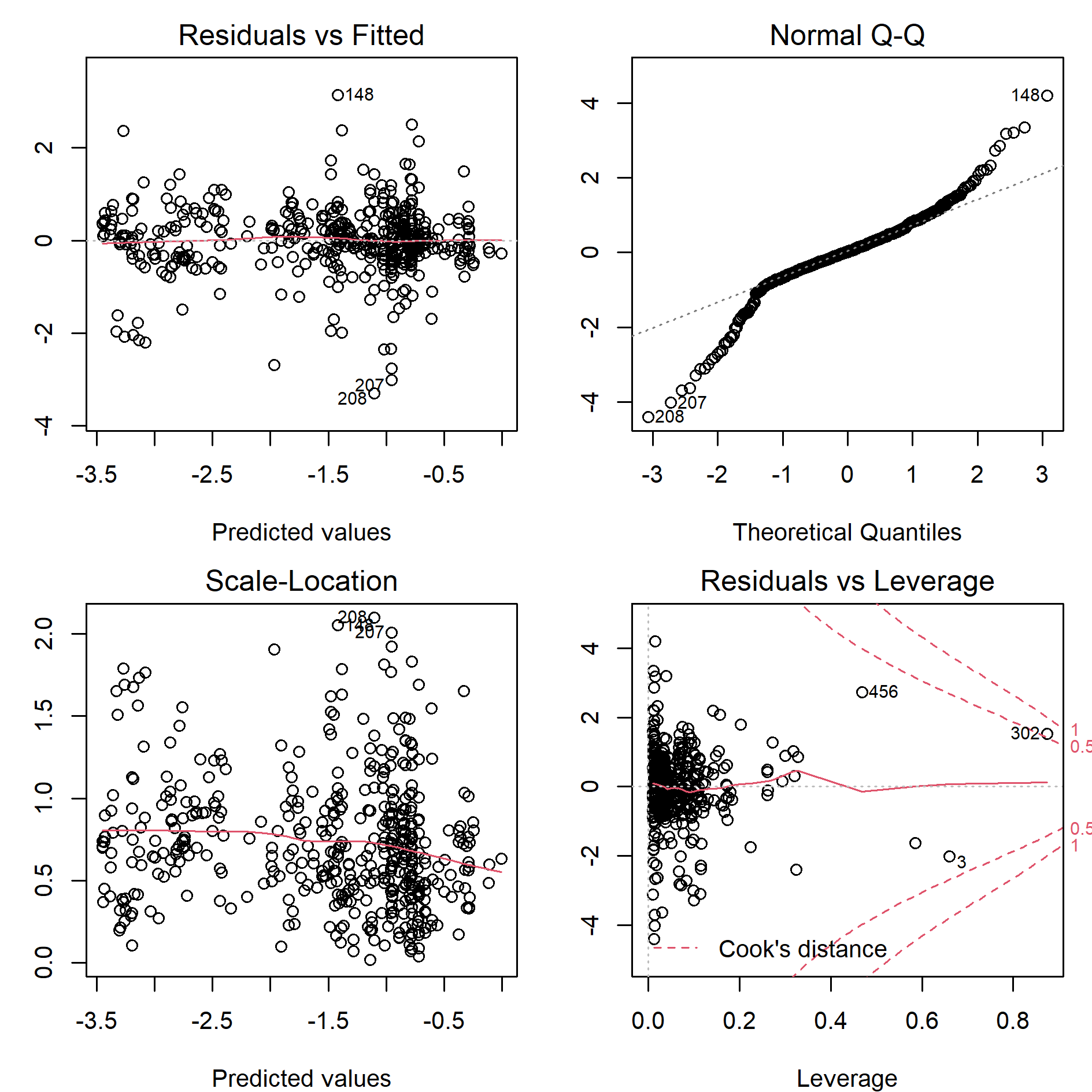


From the residual vs fitted value plot in Fig A we can see that there is no obvious violation of linear assumption. Scale-Location plot in Fig A suggested that constant variance assumption is satisfied. Based on the model diagnostic plot, we were confident that the full model was able to describe the linear association between the response and covariates. Next, we reduced model dimensionality by using stepwise model selection with BIC as the selection criteria. The best model selected based on BIC identified age lower, year, population growth, rural population (% in total population), vaccination status, and geographical location of JE cases as JE CFR drivers.

**Model formula**

$$\log\left( \frac{CFR}{1-CFR} \right)=\beta_{0}+ \beta_{1} AgeLower+ \beta_{2}Year+ \beta_{3}\log\left( Population growth \right)+ \beta_{4}\log\left( Rural Population \left( \% \right) \right)+ \beta_{5} Vaccination+\sum_{c=1}^{C} \beta_{Tc}1_{T_{i}=c}$$

**Fig B: Model Diagnostic for the Best Model selected by BIC**


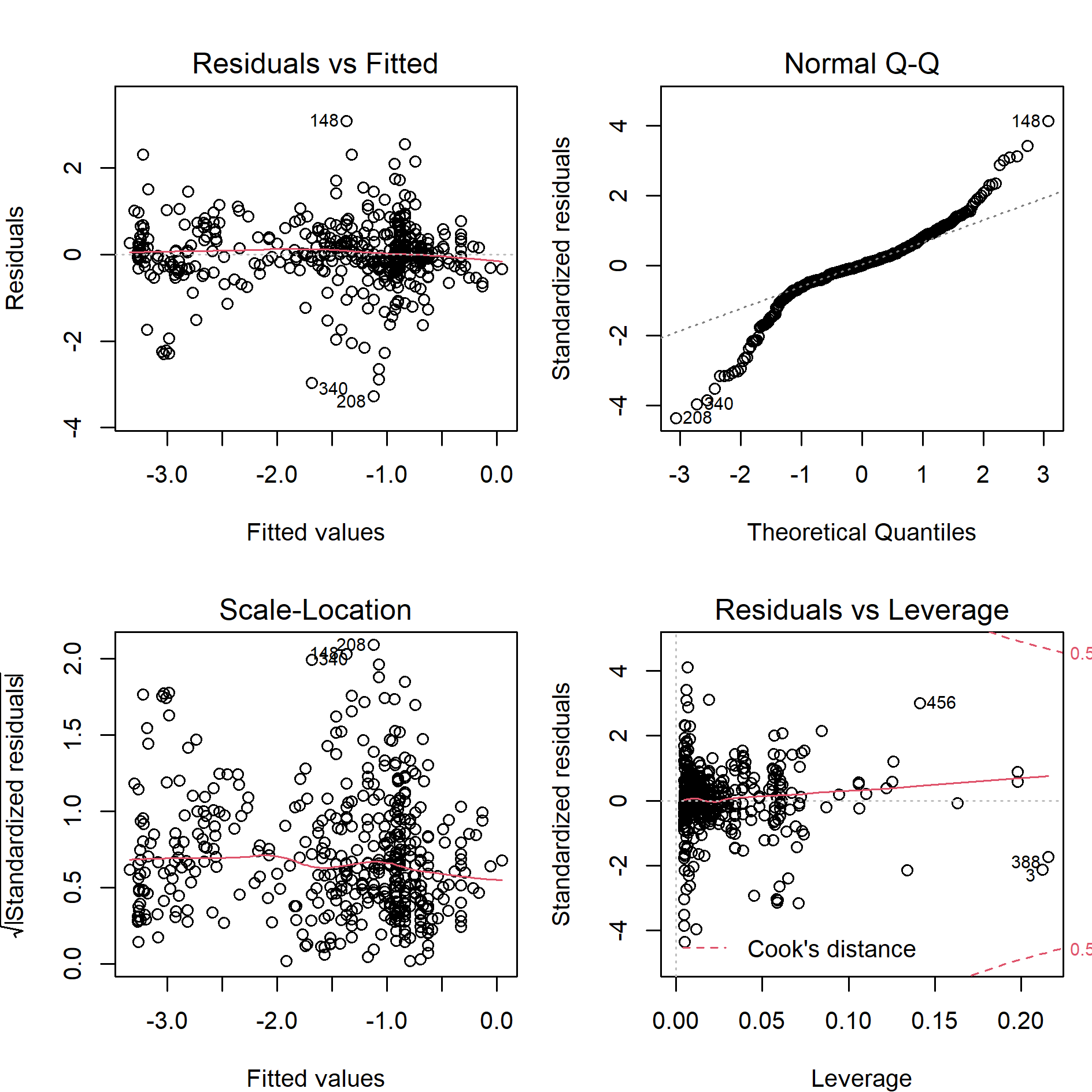


From Fig B, we found that linear association and constant variance assumption still held for the best model. But normal QQ plot (see Fig B) suggests that residuals from the best model might not normally distributed. Thus, we conducted bootstrapping residuals 2000 times to construct confidence intervals for coefficient estimates (**as shown in the *Table 3* in the manuscript**).

To check the model robustness, we ran the model without the influential observation (observation 438). Comparing Fig C with *Table 3* in the manuscript we found that there was no considerable change in terms of sign and magnitude for the coefficient estimates. Thus, we were confident that result from our model was robust against the influential observations.

**Fig C: Coefficient output of Model without the Influential Observation (438)**


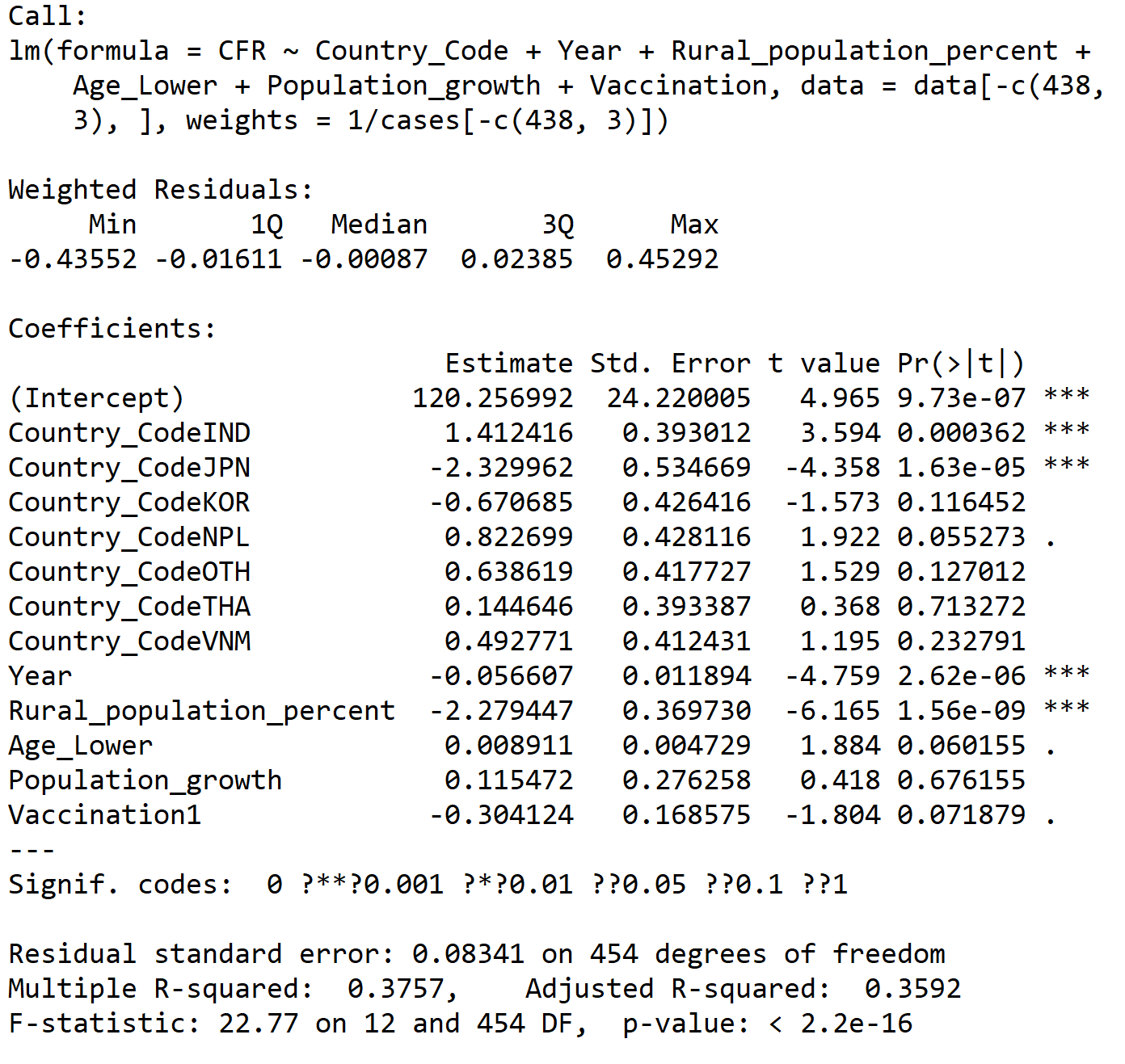


Our JE inference model treated each JE record equally even each record had different reported JE cases. The distribution of reported JE cases was heavily right-tailed and all records with more than 5000 reported JE cases came from China and India. When fitting, we wanted records from countries except China and India to get more attention. We don’t want result from China and India dominate the whole analysis. As a result, in the inference model, we did not use the JE cases to weight each record.

## Analysis for JE records with 0 deaths

According to the description in the estimation dataset, we removed JE record with 0 death. The following paragraph discussed our motivation of removing these proportion of data. And we discussed the potential consequences of this action.

In the collated dataset, we had 98 records with 0 JE death coming from 6 countries, 38 records from India, 32 records from Korea, 22 records from Japan, 4 records from Nepal, 1 record from Myanmar, and 1 record from Thailand. Initially, we included these records into the analysis. At the beginning, we regressed log odds of CFR on all covariates. However, as shown in Fig D, residuals clearly formed 3 clusters. Besides, it is clearly that nonconstant variance assumption failed (see Scale-Location plot in Fig D).

**Fig D: Model Diagnostic for the Full Model Fitted on Full JE Records**


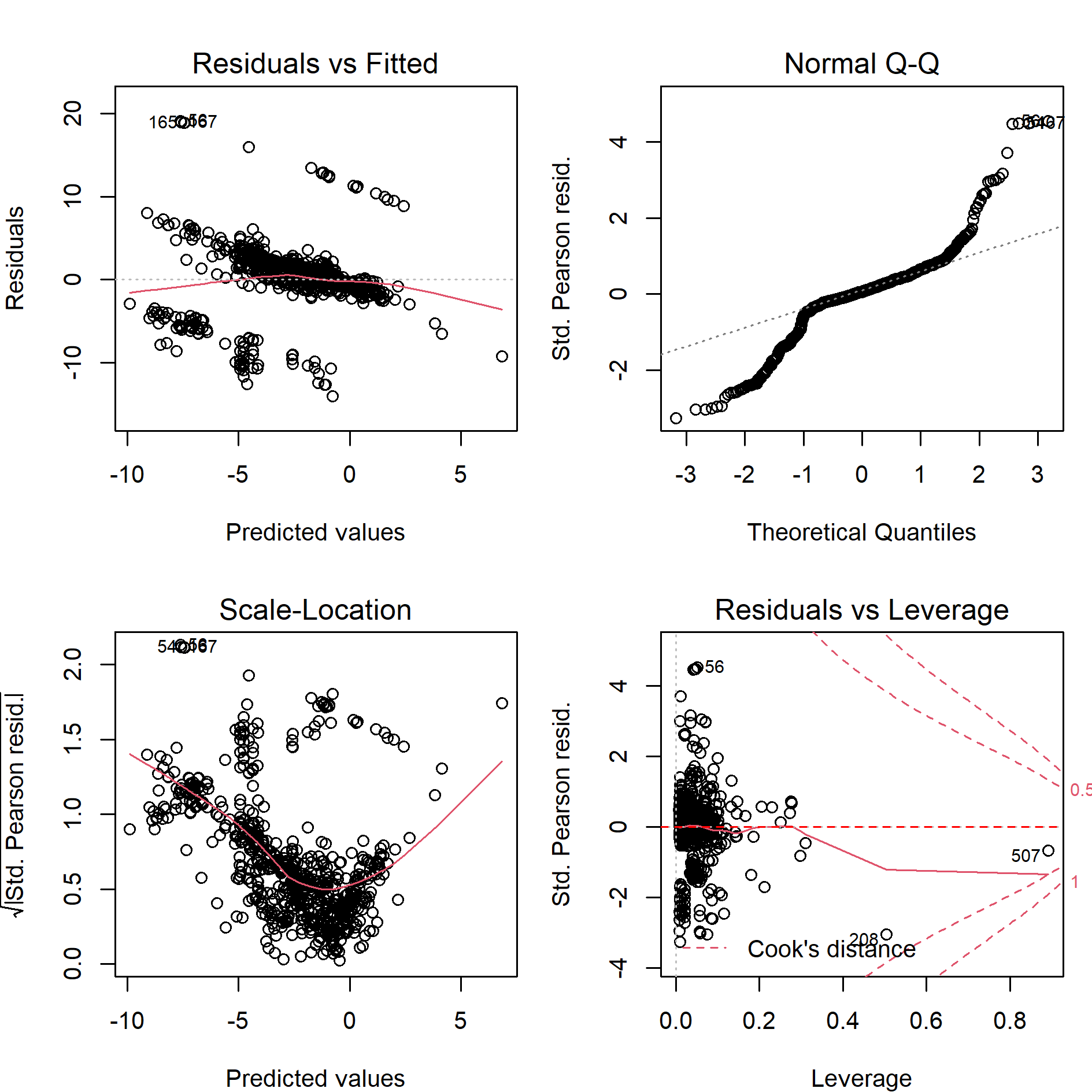


To investigate which factors caused cluster formation, for each covariate, we plotted it against log odds of CFR. However, as shown in Fig E, we did not observe any formed clusters by covariates. Moreover, there were no indication of any nonlinear relationship between the response variable and covariates. It suggests that, residuals form clusters with respect to the value of the response. Underlying mechanism that explains JE record with 0 deaths might be fundamentally differently from the general trend, which constrained the generalizability of modelling result. Thus, we decided to exclude records with 0 death into analysis. As a consequence, JE CFR estimated by our model naturally formed an upper bound for the true value. Even this “upper bound” of JE CFR is lower than the currently available estimates. This strengthens the necessity of updating the current JE CFR estimates.

**Fig E: Scatter plot of Covariates in the Full Model against Log Odds of CFR**


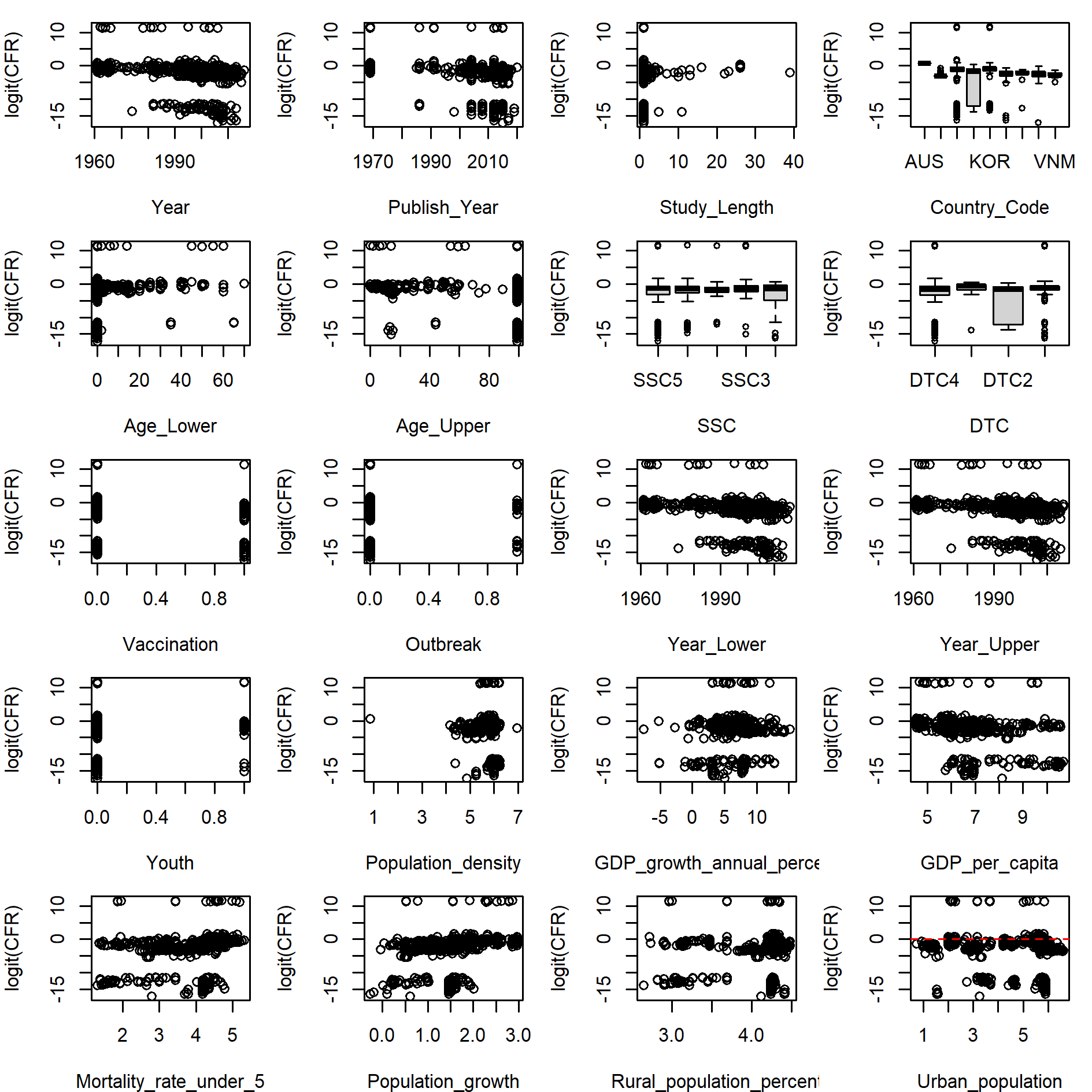


# Prediction model

For each bootstrapped dataset, we fitted a LASSO regression(14), principal component regression(15), gradient boosting machine(16), neural network(17), and stacking(18). For stacked regression, we used the aforementioned four models as base leaner and we attempted two meta-learners, one is multiple linear regression, the other is a weighting-based algorithm. The weighting algorithm is defined as follows: if JE CFR predictions from all base leaners were greater than 0.6, the prediction of meta-learner was the minimum value of the base learns’ prediction results; if all base-learns’ predictions were lower than 0.02, the prediction of meta-learner was the maximum value of the base learners’ prediction result. Otherwise, the prediction result of meta-learner was the simple average of base leaners’ prediction results that were between 0.02 and 0.6. We chose 0.02 and 0.6 as cut points because these two values correspond to the lower 2.5% and upper 2.5% quantile of the JE CFR records. The biggest gains are highly likely to be produced when stacking base learners that have high variability, and uncorrelated, predicted values. Then we used the fitted models to make JE CFR predictions on the prediction datasets. Thus, for each JE endemic country in every year between 1961 and 2018, we had 2000 predicted values for each model. For each model fitted on each bootstrapped dataset, we computed its log binomial likelihood across all JE CFR records (Equation 1). $\hat{y}_{c,t, m,b}$ was the predicted JE CFR for country c in year t by model m fitted on the bootstrapped dataset b. In total we had 2000 log binomial likelihood for each model. We chose the model with large median log binomial likelihood and log binomial likelihood distribution with small variance as the best prediction model. We repeated the above procedure with and without year as the predictor for sensitivity analysis.

$$\log\left( \prod_{\left\{ c,t \right\}\in X_{n\times p}} Bin\left( n= {Case}_{c,t}, x={Death}_{c,t},p= \hat{y}_{c,t, m,b} \right) \right) (1)$$

## Log binomial likelihood distribution

| Model | Distribution of binomial log likelihood | | | |
| --- | --- | --- | --- | --- |
|  | Without year | | With year | |
|  | Median | 95% CI | Median | 95% CI |
| LASSO | -9034 | (-7006221, -7260) | -10921 | (-10259803, -7260) |
| PCR | -98871 | (-293247, -10374) | -66136 | (-273867, -9378) |
| GBM | -24177 | (-51047, -11261) | -23159 | (-50092, -10971) |
| NN | -140811 | (-2703911, -26363) | -128928 | (-2109318, -24439) |
| Stacking (MLR) | -24799 | (-56287, -11407) | -23988 | (-55489, -11238) |
| Stacking (Weighting algorithm) | -19274 | (-73941, -8886) | -19617 | (-73664, -8853) |

The result showed that LASSO regression (yellow colored cell) had the largest median of log binomial distribution. However, it was associated with large variance. The green colored cells ranked the second and third largest median log binomial distribution. Because the log binomial distribution of the stacking with weighting algorithm without year as predictors had a smaller variance, it was chosen as the best prediction model.

# Projection Model

The projection model was consistent with the prediction model. We used stacking with weight algorithm as meta-learner and without year as predictor as the projection model. Projectors are vaccination status fixed as 2018’s value, population density, population growth rate, under-5 mortality, GDP per capita, GDP growth rate, and dummy indicators to denote whether we predict values for India.

**Model architecture**


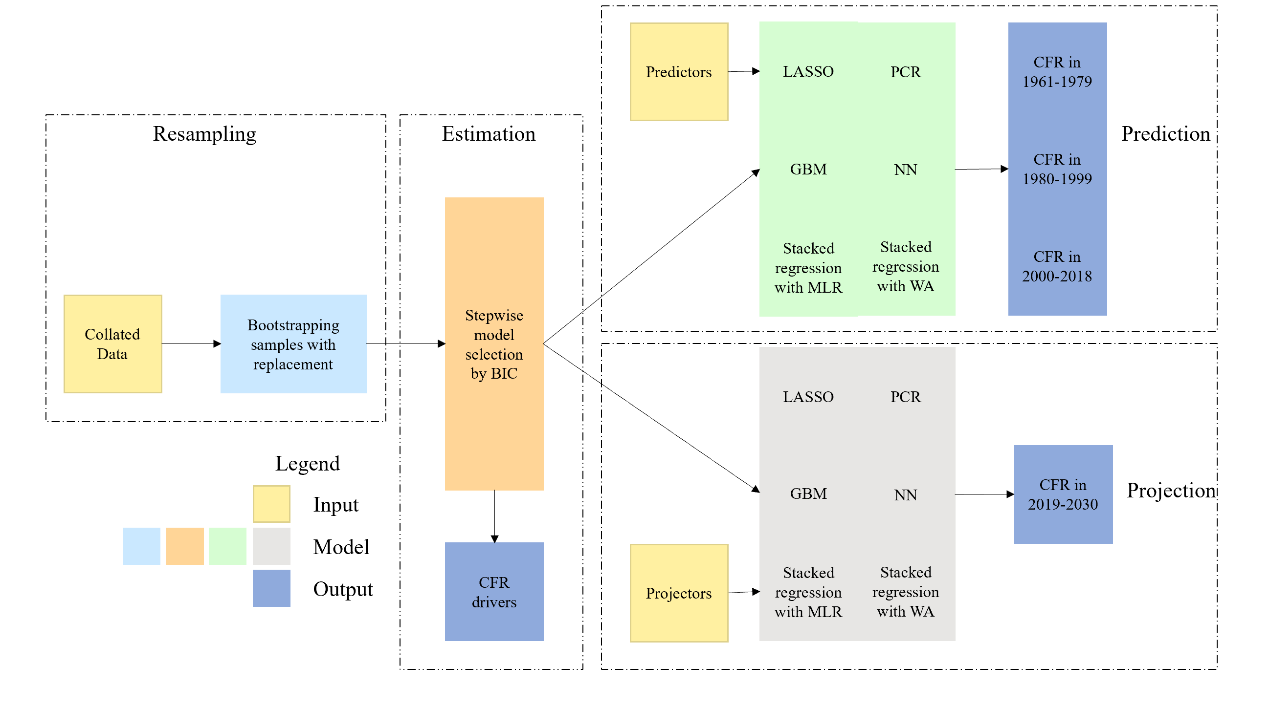

Supplement: S1 File — (DOCX) [file pntd.0010361.s011.docx]
