# Supplementary material for: Estimates of Japanese Encephalitis mortality and morbidity: A systematic review and modeling analysis
Source: PLoS Negl Trop Dis. 2022 May 25;16(5):e0010361. doi: 10.1371/journal.pntd.0010361 (PMC9173604; doi:10.1371/journal.pntd.0010361)
Supplement: S1 Included Characteristics — (DOCX) [file pntd.0010361.s013.docx]

S1_Included_Characteristics: Table of detailed description of all the records extracted from the studies included in the CFR literature review

| Reference | Year | Publish  Year | Study  Length | Country | Age  Lower | Age  Upper | CFR | SSC | DTC | Vaccination | Cases | Deaths | Outbreak | Year  Lower | Year  Upper |
| --- | --- | --- | --- | --- | --- | --- | --- | --- | --- | --- | --- | --- | --- | --- | --- |
| (1) | 1997 | 2000 | 1 | Nepal | 16 | 99 | 0.13174 | SSC3 | DTC4 | 0 | 1632 | 215 | 0 | 1997 | 1997 |
| (2) | 2013 | 2017 | 3 | India | 0 | 99 | 0.149425 | SSC2 | DTC3 | 1 | 696 | 104 | 0 | 2012 | 2014 |
| (3) | 2002 | 2008 | 7 | Malaysia | 0 | 99 | 0.084746 | SSC2 | DTC3 | 0 | 118 | 10 | 0 | 1999 | 2005 |
| (4) | 1961 | 1969 | 1 | South Korea | 1 | 1 | 0.242424 | SSC5 | DTC4 | 0 | 33 | 8 | 0 | 1961 | 1961 |
|  | 1964 | 1969 | 1 | South Korea | 1 | 1 | 0.34 | SSC5 | DTC3 | 0 | 50 | 17 | 0 | 1964 | 1964 |
|  | 1966 | 1969 | 1 | South Korea | 1 | 1 | 0.225 | SSC5 | DTC3 | 0 | 80 | 18 | 0 | 1966 | 1966 |
|  | 1961 | 1969 | 1 | South Korea | 2 | 2 | 0.372881 | SSC5 | DTC4 | 0 | 59 | 22 | 0 | 1961 | 1961 |
|  | 1962 | 1969 | 1 | South Korea | 2 | 2 | 0.323529 | SSC5 | DTC4 | 0 | 34 | 11 | 0 | 1962 | 1962 |
|  | 1964 | 1969 | 1 | South Korea | 2 | 2 | 0.308411 | SSC5 | DTC3 | 0 | 107 | 33 | 0 | 1964 | 1964 |
|  | 1965 | 1969 | 1 | South Korea | 2 | 2 | 0.333333 | SSC5 | DTC3 | 0 | 27 | 9 | 0 | 1965 | 1965 |
|  | 1966 | 1969 | 1 | South Korea | 2 | 2 | 0.253731 | SSC5 | DTC3 | 0 | 134 | 34 | 0 | 1966 | 1966 |
|  | 1961 | 1969 | 1 | South Korea | 3 | 3 | 0.345455 | SSC5 | DTC4 | 0 | 110 | 38 | 0 | 1961 | 1961 |
|  | 1962 | 1969 | 1 | South Korea | 3 | 3 | 0.333333 | SSC5 | DTC4 | 0 | 84 | 28 | 0 | 1962 | 1962 |
|  | 1964 | 1969 | 1 | South Korea | 3 | 3 | 0.328704 | SSC5 | DTC3 | 0 | 216 | 71 | 0 | 1964 | 1964 |
|  | 1965 | 1969 | 1 | South Korea | 3 | 3 | 0.394737 | SSC5 | DTC3 | 0 | 38 | 15 | 0 | 1965 | 1965 |
|  | 1966 | 1969 | 1 | South Korea | 3 | 3 | 0.231047 | SSC5 | DTC3 | 0 | 277 | 64 | 0 | 1966 | 1966 |
|  | 1961 | 1969 | 1 | South Korea | 4 | 4 | 0.325843 | SSC5 | DTC3 | 0 | 89 | 29 | 0 | 1961 | 1961 |
|  | 1962 | 1969 | 1 | South Korea | 4 | 4 | 0.314815 | SSC5 | DTC3 | 0 | 108 | 34 | 0 | 1962 | 1962 |
|  | 1964 | 1969 | 1 | South Korea | 4 | 4 | 0.299169 | SSC5 | DTC3 | 0 | 361 | 108 | 0 | 1964 | 1964 |
|  | 1965 | 1969 | 1 | South Korea | 4 | 4 | 0.409091 | SSC5 | DTC3 | 0 | 88 | 36 | 0 | 1965 | 1965 |
|  | 1966 | 1969 | 1 | South Korea | 4 | 4 | 0.257081 | SSC5 | DTC3 | 0 | 459 | 118 | 0 | 1966 | 1966 |
|  | 1961 | 1969 | 1 | South Korea | 5 | 5 | 0.433628 | SSC5 | DTC4 | 0 | 113 | 49 | 0 | 1961 | 1961 |
|  | 1962 | 1969 | 1 | South Korea | 5 | 5 | 0.32 | SSC5 | DTC4 | 0 | 125 | 40 | 0 | 1962 | 1962 |
|  | 1964 | 1969 | 1 | South Korea | 5 | 5 | 0.314655 | SSC5 | DTC3 | 0 | 464 | 146 | 0 | 1964 | 1964 |
|  | 1965 | 1969 | 1 | South Korea | 5 | 5 | 0.36036 | SSC5 | DTC3 | 0 | 111 | 40 | 0 | 1965 | 1965 |
|  | 1966 | 1969 | 1 | South Korea | 5 | 5 | 0.270955 | SSC5 | DTC3 | 0 | 513 | 139 | 0 | 1966 | 1966 |
|  | 1961 | 1969 | 1 | South Korea | 6 | 6 | 0.347107 | SSC5 | DTC4 | 0 | 121 | 42 | 0 | 1961 | 1961 |
|  | 1962 | 1969 | 1 | South Korea | 6 | 6 | 0.347107 | SSC5 | DTC4 | 0 | 121 | 42 | 0 | 1962 | 1962 |
|  | 1964 | 1969 | 1 | South Korea | 6 | 6 | 0.321513 | SSC5 | DTC3 | 0 | 423 | 136 | 0 | 1964 | 1964 |
|  | 1965 | 1969 | 1 | South Korea | 6 | 6 | 0.419753 | SSC5 | DTC3 | 0 | 81 | 34 | 0 | 1965 | 1965 |
|  | 1966 | 1969 | 1 | South Korea | 6 | 6 | 0.299632 | SSC5 | DTC3 | 0 | 544 | 163 | 0 | 1966 | 1966 |
|  | 1961 | 1969 | 1 | South Korea | 7 | 7 | 0.37963 | SSC5 | DTC4 | 0 | 108 | 41 | 0 | 1961 | 1961 |
|  | 1962 | 1969 | 1 | South Korea | 7 | 7 | 0.315789 | SSC5 | DTC4 | 0 | 95 | 30 | 0 | 1962 | 1962 |
|  | 1964 | 1969 | 1 | South Korea | 7 | 7 | 0.327381 | SSC5 | DTC3 | 0 | 336 | 110 | 0 | 1964 | 1964 |
|  | 1965 | 1969 | 1 | South Korea | 7 | 7 | 0.313433 | SSC5 | DTC3 | 0 | 67 | 21 | 0 | 1965 | 1965 |
|  | 1966 | 1969 | 1 | South Korea | 7 | 7 | 0.278826 | SSC5 | DTC3 | 0 | 477 | 133 | 0 | 1966 | 1966 |
|  | 1961 | 1969 | 1 | South Korea | 8 | 8 | 0.28169 | SSC5 | DTC4 | 0 | 71 | 20 | 0 | 1961 | 1961 |
|  | 1962 | 1969 | 1 | South Korea | 8 | 8 | 0.361702 | SSC5 | DTC4 | 0 | 94 | 34 | 0 | 1962 | 1962 |
|  | 1964 | 1969 | 1 | South Korea | 8 | 8 | 0.354701 | SSC5 | DTC3 | 0 | 234 | 83 | 0 | 1964 | 1964 |
|  | 1965 | 1969 | 1 | South Korea | 8 | 8 | 0.364706 | SSC5 | DTC3 | 0 | 85 | 31 | 0 | 1965 | 1965 |
|  | 1966 | 1969 | 1 | South Korea | 8 | 8 | 0.248344 | SSC5 | DTC3 | 0 | 302 | 75 | 0 | 1966 | 1966 |
|  | 1961 | 1969 | 1 | South Korea | 9 | 9 | 0.355263 | SSC5 | DTC4 | 0 | 76 | 27 | 0 | 1961 | 1961 |
|  | 1962 | 1969 | 1 | South Korea | 9 | 9 | 0.290323 | SSC5 | DTC4 | 0 | 62 | 18 | 0 | 1962 | 1962 |
|  | 1964 | 1969 | 1 | South Korea | 9 | 9 | 0.322222 | SSC5 | DTC3 | 0 | 180 | 58 | 0 | 1964 | 1964 |
|  | 1965 | 1969 | 1 | South Korea | 9 | 9 | 0.37931 | SSC5 | DTC3 | 0 | 58 | 22 | 0 | 1965 | 1965 |
|  | 1966 | 1969 | 1 | South Korea | 9 | 9 | 0.211207 | SSC5 | DTC3 | 0 | 232 | 49 | 0 | 1966 | 1966 |
|  | 1961 | 1969 | 1 | South Korea | 10 | 10 | 0.338983 | SSC5 | DTC4 | 0 | 59 | 20 | 0 | 1961 | 1961 |
|  | 1962 | 1969 | 1 | South Korea | 10 | 10 | 0.412698 | SSC5 | DTC4 | 0 | 63 | 26 | 0 | 1962 | 1962 |
|  | 1964 | 1969 | 1 | South Korea | 10 | 10 | 0.428571 | SSC5 | DTC3 | 0 | 126 | 54 | 0 | 1964 | 1964 |
|  | 1965 | 1969 | 1 | South Korea | 10 | 10 | 0.232558 | SSC5 | DTC3 | 0 | 43 | 10 | 0 | 1965 | 1965 |
|  | 1966 | 1969 | 1 | South Korea | 10 | 10 | 0.333333 | SSC5 | DTC3 | 0 | 162 | 54 | 0 | 1966 | 1966 |
|  | 1961 | 1969 | 1 | South Korea | 11 | 11 | 0.354839 | SSC5 | DTC3 | 0 | 31 | 11 | 0 | 1961 | 1961 |
|  | 1962 | 1969 | 1 | South Korea | 11 | 11 | 0.372093 | SSC5 | DTC3 | 0 | 43 | 16 | 0 | 1962 | 1962 |
|  | 1964 | 1969 | 1 | South Korea | 11 | 11 | 0.294737 | SSC5 | DTC3 | 0 | 95 | 28 | 0 | 1964 | 1964 |
|  | 1965 | 1969 | 1 | South Korea | 11 | 11 | 0.304348 | SSC5 | DTC3 | 0 | 23 | 7 | 0 | 1965 | 1965 |
|  | 1966 | 1969 | 1 | South Korea | 11 | 11 | 0.30303 | SSC5 | DTC3 | 0 | 99 | 30 | 0 | 1966 | 1966 |
|  | 1961 | 1969 | 1 | South Korea | 12 | 12 | 0.24 | SSC5 | DTC4 | 0 | 25 | 6 | 0 | 1961 | 1961 |
|  | 1962 | 1969 | 1 | South Korea | 12 | 12 | 0.185185 | SSC5 | DTC4 | 0 | 27 | 5 | 0 | 1962 | 1962 |
|  | 1964 | 1969 | 1 | South Korea | 12 | 12 | 0.287356 | SSC5 | DTC3 | 0 | 87 | 25 | 0 | 1964 | 1964 |
|  | 1965 | 1969 | 1 | South Korea | 12 | 12 | 0.5 | SSC5 | DTC3 | 0 | 22 | 11 | 0 | 1965 | 1965 |
|  | 1966 | 1969 | 1 | South Korea | 12 | 12 | 0.289474 | SSC5 | DTC3 | 0 | 76 | 22 | 0 | 1966 | 1966 |
|  | 1961 | 1969 | 1 | South Korea | 13 | 13 | 0.130435 | SSC5 | DTC4 | 0 | 23 | 3 | 0 | 1961 | 1961 |
|  | 1962 | 1969 | 1 | South Korea | 13 | 13 | 0.25 | SSC5 | DTC4 | 0 | 28 | 7 | 0 | 1962 | 1962 |
|  | 1964 | 1969 | 1 | South Korea | 13 | 13 | 0.285714 | SSC5 | DTC3 | 0 | 42 | 12 | 0 | 1964 | 1964 |
|  | 1966 | 1969 | 1 | South Korea | 13 | 13 | 0.173077 | SSC5 | DTC3 | 0 | 52 | 9 | 0 | 1966 | 1966 |
|  | 1962 | 1969 | 1 | South Korea | 14 | 14 | 0.304348 | SSC5 | DTC4 | 0 | 23 | 7 | 0 | 1962 | 1962 |
|  | 1964 | 1969 | 1 | South Korea | 14 | 14 | 0.272727 | SSC5 | DTC3 | 0 | 33 | 9 | 0 | 1964 | 1964 |
|  | 1966 | 1969 | 1 | South Korea | 14 | 14 | 0.26087 | SSC5 | DTC3 | 0 | 46 | 12 | 0 | 1966 | 1966 |
|  | 1961 | 1969 | 1 | South Korea | 15 | 19 | 0.409091 | SSC5 | DTC4 | 0 | 44 | 18 | 0 | 1961 | 1961 |
|  | 1962 | 1969 | 1 | South Korea | 15 | 19 | 0.414634 | SSC5 | DTC4 | 0 | 41 | 17 | 0 | 1962 | 1962 |
|  | 1964 | 1969 | 1 | South Korea | 15 | 19 | 0.337079 | SSC5 | DTC3 | 0 | 89 | 30 | 0 | 1964 | 1964 |
|  | 1965 | 1969 | 1 | South Korea | 15 | 19 | 0.448276 | SSC5 | DTC3 | 0 | 29 | 13 | 0 | 1965 | 1965 |
|  | 1966 | 1969 | 1 | South Korea | 15 | 19 | 0.189873 | SSC5 | DTC3 | 0 | 79 | 15 | 0 | 1966 | 1966 |
|  | 1961 | 1969 | 1 | South Korea | 20 | 24 | 0.372549 | SSC5 | DTC3 | 0 | 51 | 19 | 0 | 1961 | 1961 |
|  | 1964 | 1969 | 1 | South Korea | 20 | 24 | 0.458333 | SSC5 | DTC3 | 0 | 24 | 11 | 0 | 1964 | 1964 |
|  | 1966 | 1969 | 1 | South Korea | 20 | 24 | 0.214286 | SSC5 | DTC3 | 0 | 28 | 6 | 0 | 1966 | 1966 |
|  | 1965 | 1969 | 1 | Japan | 0 | 4 | 0.192982 | SSC5 | DTC4 | 0 | 57 | 11 | 0 | 1965 | 1965 |
|  | 1965 | 1969 | 1 | Japan | 5 | 9 | 0.16 | SSC5 | DTC4 | 0 | 75 | 12 | 0 | 1965 | 1965 |
|  | 1965 | 1969 | 1 | Japan | 10 | 14 | 0.121951 | SSC5 | DTC4 | 0 | 41 | 5 | 0 | 1965 | 1965 |
|  | 1965 | 1969 | 1 | Japan | 15 | 19 | 0.22973 | SSC5 | DTC4 | 0 | 74 | 17 | 0 | 1965 | 1965 |
|  | 1965 | 1969 | 1 | Japan | 20 | 29 | 0.12 | SSC5 | DTC4 | 0 | 100 | 12 | 0 | 1965 | 1965 |
|  | 1965 | 1969 | 1 | Japan | 30 | 39 | 0.222222 | SSC5 | DTC4 | 0 | 63 | 14 | 0 | 1965 | 1965 |
|  | 1965 | 1969 | 1 | Japan | 40 | 49 | 0.340426 | SSC5 | DTC4 | 0 | 47 | 16 | 0 | 1965 | 1965 |
|  | 1965 | 1969 | 1 | Japan | 50 | 59 | 0.447368 | SSC5 | DTC4 | 0 | 76 | 34 | 0 | 1965 | 1965 |
|  | 1965 | 1969 | 1 | Japan | 60 | 69 | 0.473333 | SSC5 | DTC4 | 0 | 150 | 71 | 0 | 1965 | 1965 |
|  | 1965 | 1969 | 1 | Japan | 70 | 99 | 0.540323 | SSC5 | DTC4 | 0 | 124 | 67 | 0 | 1965 | 1965 |
| (5) | 2016 | 2018 | 1 | India | 0 | 13 | 0.211429 | SSC4 | DTC3 | 1 | 175 | 37 | 1 | 2016 | 2016 |
| (6) | 2007 | 2017 | 1 | Nepal | 0 | 99 | 0.138009 | SSC4 | DTC3 | 0 | 442 | 61 | 0 | 2007 | 2007 |
|  | 2008 | 2017 | 1 | Nepal | 0 | 99 | 0.115044 | SSC4 | DTC3 | 1 | 339 | 39 | 0 | 2008 | 2008 |
|  | 2009 | 2017 | 1 | Nepal | 0 | 99 | 0.061644 | SSC4 | DTC3 | 1 | 146 | 9 | 0 | 2009 | 2009 |
|  | 2010 | 2017 | 1 | Nepal | 0 | 99 | 0.005076 | SSC4 | DTC3 | 1 | 197 | 1 | 0 | 2010 | 2010 |
|  | 2014 | 2017 | 1 | Nepal | 0 | 99 | 0.044248 | SSC4 | DTC3 | 1 | 226 | 10 | 0 | 2014 | 2014 |
|  | 2015 | 2017 | 1 | Nepal | 0 | 99 | 0.007194 | SSC4 | DTC3 | 1 | 139 | 1 | 0 | 2015 | 2015 |
| (7) | 2009 | 2011 | 3 | India | 0 | 15 | 0.044444 | SSC2 | DTC2 | 1 | 45 | 2 | 0 | 2008 | 2010 |
|  | 2009 | 2011 | 3 | India | 16 | 99 | 0.081633 | SSC2 | DTC2 | 1 | 98 | 8 | 0 | 2008 | 2010 |
| (8) | 2013 | 2014 | 1 | China | 0 | 99 | 0.027027 | SSC5 | DTC4 | 1 | 407 | 11 | 1 | 2013 | 2013 |
| (9) | 1972 | 2012 | 1 | China | 0 | 99 | 0.187845 | SSC1 | DTC4 | 0 | 4344 | 816 | 0 | 1972 | 1972 |
|  | 1990 | 2012 | 39 | China | 0 | 99 | 0.109021 | SSC1 | DTC4 | 0 | 68427 | 7460 | 0 | 1971 | 2009 |
| (10) | 2007 | 2011 | 2 | Cambodia | 1 | 15 | 0.14 | SSC2 | DTC3 | 0 | 50 | 7 | 0 | 2006 | 2007 |
|  | 2007 | 2011 | 3 | Vietnam | 2 | 73 | 0.068966 | SSC3 | DTC3 | 0 | 29 | 2 | 0 | 2006 | 2008 |
| (11) | 2007 | 2011 | 3 | Nepal | 1 | 14 | 0.095238 | SSC3 | DTC3 | 0 | 42 | 4 | 0 | 2006 | 2008 |
| (12) | 2002 | 2006 | 3 | Indonesia | 0 | 11 | 0.104651 | SSC2 | DTC3 | 0 | 86 | 9 | 0 | 2001 | 2003 |
| (13) | 1982 | 2000 | 1 | Korea | 0 | 99 | 0.033417 | SSC5 | DTC4 | 0 | 1197 | 40 | 0 | 1982 | 1982 |
| (14) | 2012 | 2013 | 1 | India | 0 | 12 | 0.147541 | SSC3 | DTC3 | 1 | 61 | 9 | 0 | 2012 | 2012 |
| (15) | 2004 | 2010 | 1 | Vietnam | 0 | 99 | 0.16 | SSC3 | DTC1 | 0 | 50 | 8 | 0 | 2004 | 2004 |
|  | 2002 | 2014 | 13 | Vietnam | 15 | 99 | 0.055556 | SSC3 | DTC1 | 0 | 36 | 2 | 0 | 1996 | 2008 |
| (16) | 2010 | 2013 | 1 | Korea | 0 | 99 | 0.269231 | SSC5 | DTC4 | 1 | 26 | 7 | 1 | 2010 | 2010 |
| (17) | 2010 | 2013 | 2 | Cambodia | 0 | 15 | 0.014085 | SSC3 | DTC3 | 1 | 71 | 1 | 0 | 2009 | 2010 |
| (18) | 2005 | 2013 | 1 | Nepal | 0 | 99 | 0.073306 | SSC1 | DTC3 | 0 | 723 | 53 | 0 | 2005 | 2005 |
|  | 2006 | 2013 | 1 | Nepal | 0 | 99 | 0.142373 | SSC1 | DTC3 | 0 | 295 | 42 | 0 | 2006 | 2006 |
|  | 2007 | 2013 | 1 | Nepal | 0 | 99 | 0.167123 | SSC1 | DTC3 | 0 | 365 | 61 | 0 | 2007 | 2007 |
|  | 2008 | 2013 | 1 | Nepal | 0 | 99 | 0.126214 | SSC1 | DTC3 | 1 | 309 | 39 | 0 | 2008 | 2008 |
|  | 2009 | 2013 | 1 | Nepal | 0 | 99 | 0.059603 | SSC1 | DTC3 | 1 | 151 | 9 | 0 | 2009 | 2009 |
|  | 2010 | 2013 | 1 | Nepal | 0 | 99 | 0.005076 | SSC1 | DTC3 | 1 | 197 | 1 | 0 | 2010 | 2010 |
| (19) | 2006 | 2007 | 1 | Nepal | 0 | 99 | 0.2 | SSC1 | DTC3 | 0 | 40 | 8 | 0 | 2006 | 2006 |
| (20) | 2009 | 2015 | 1 | India | 0 | 99 | 0.25 | SSC5 | DTC4 | 1 | 76 | 19 | 0 | 2009 | 2009 |
| (21) | 1991 | 2012 | 26 | India | 15 | 20 | 0.338912 | SSC4 | DTC1 | 0 | 239 | 81 | 0 | 1978 | 2003 |
|  | 1991 | 2012 | 26 | India | 21 | 30 | 0.408867 | SSC4 | DTC1 | 0 | 203 | 83 | 0 | 1978 | 2003 |
|  | 1991 | 2012 | 26 | India | 31 | 40 | 0.373239 | SSC4 | DTC1 | 0 | 142 | 53 | 0 | 1978 | 2003 |
|  | 1991 | 2012 | 26 | India | 41 | 50 | 0.494505 | SSC4 | DTC1 | 0 | 91 | 45 | 0 | 1978 | 2003 |
|  | 1991 | 2012 | 26 | India | 51 | 99 | 0.513158 | SSC4 | DTC1 | 0 | 76 | 39 | 0 | 1978 | 2003 |
|  | 1991 | 2012 | 26 | India | 15 | 20 | 0.401961 | SSC4 | DTC1 | 0 | 102 | 41 | 0 | 1978 | 2003 |
|  | 1991 | 2012 | 26 | India | 21 | 30 | 0.448485 | SSC4 | DTC1 | 0 | 165 | 74 | 0 | 1978 | 2003 |
|  | 1991 | 2012 | 26 | India | 31 | 40 | 0.533981 | SSC4 | DTC1 | 0 | 103 | 55 | 0 | 1978 | 2003 |
|  | 1991 | 2012 | 26 | India | 41 | 50 | 0.609756 | SSC4 | DTC1 | 0 | 41 | 25 | 0 | 1978 | 2003 |
|  | 1991 | 2012 | 26 | India | 51 | 99 | 0.405405 | SSC4 | DTC1 | 0 | 37 | 15 | 0 | 1978 | 2003 |
| (22) | 1996 | 2004 | 1 | India | 0 | 99 | 0.264025 | SSC5 | DTC4 | 0 | 2246 | 593 | 0 | 1996 | 1996 |
|  | 1997 | 2004 | 1 | India | 0 | 99 | 0.251192 | SSC5 | DTC4 | 0 | 2516 | 632 | 0 | 1997 | 1997 |
|  | 1998 | 2004 | 1 | India | 0 | 99 | 0.242584 | SSC5 | DTC4 | 0 | 2090 | 507 | 0 | 1998 | 1998 |
|  | 1999 | 2004 | 1 | India | 0 | 99 | 0.198366 | SSC5 | DTC4 | 0 | 3428 | 680 | 0 | 1999 | 1999 |
|  | 2000 | 2004 | 1 | India | 0 | 99 | 0.214423 | SSC5 | DTC4 | 0 | 2593 | 556 | 0 | 2000 | 2000 |
|  | 2001 | 2004 | 1 | India | 0 | 99 | 0.258753 | SSC5 | DTC4 | 0 | 1171 | 303 | 0 | 2001 | 2001 |
|  | 2002 | 2004 | 1 | India | 0 | 99 | 0.19717 | SSC5 | DTC4 | 0 | 3251 | 641 | 0 | 2002 | 2002 |
| (23) | 1978 | 2012 | 1 | India | 0 | 99 | 0.296407 | SSC5 | DTC4 | 0 | 1002 | 297 | 0 | 1978 | 1978 |
|  | 2005 | 2012 | 1 | India | 0 | 99 | 0.247484 | SSC5 | DTC4 | 0 | 6061 | 1500 | 0 | 2005 | 2005 |
|  | 2007 | 2012 | 1 | India | 0 | 99 | 0.213294 | SSC5 | DTC4 | 0 | 3024 | 645 | 0 | 2007 | 2007 |
|  | 2004 | 2012 | 1 | India | 0 | 99 | 0.27234 | SSC5 | DTC4 | 0 | 235 | 64 | 0 | 2004 | 2004 |
|  | 2005 | 2012 | 1 | India | 0 | 99 | 0.358621 | SSC5 | DTC4 | 0 | 145 | 52 | 0 | 2005 | 2005 |
|  | 2006 | 2012 | 1 | India | 0 | 99 | 0.303571 | SSC5 | DTC4 | 0 | 392 | 119 | 0 | 2006 | 2006 |
|  | 2007 | 2012 | 1 | India | 0 | 99 | 0.313679 | SSC5 | DTC4 | 0 | 424 | 133 | 0 | 2007 | 2007 |
|  | 2008 | 2012 | 1 | India | 0 | 99 | 0.310345 | SSC5 | DTC4 | 1 | 319 | 99 | 0 | 2008 | 2008 |
|  | 2004 | 2012 | 1 | India | 0 | 99 | 0.329412 | SSC5 | DTC4 | 0 | 85 | 28 | 0 | 2004 | 2004 |
|  | 2005 | 2012 | 1 | India | 0 | 99 | 0.333333 | SSC5 | DTC4 | 0 | 192 | 64 | 0 | 2005 | 2005 |
|  | 2006 | 2012 | 1 | India | 0 | 99 | 0.142857 | SSC5 | DTC4 | 0 | 21 | 3 | 0 | 2006 | 2006 |
|  | 2007 | 2012 | 1 | India | 0 | 99 | 0.488095 | SSC5 | DTC4 | 0 | 336 | 164 | 0 | 2007 | 2007 |
|  | 2008 | 2012 | 1 | India | 0 | 99 | 0.221675 | SSC5 | DTC4 | 1 | 203 | 45 | 0 | 2008 | 2008 |
|  | 2004 | 2012 | 1 | India | 0 | 99 | 0.72973 | SSC5 | DTC4 | 0 | 37 | 27 | 0 | 2004 | 2004 |
|  | 2005 | 2012 | 1 | India | 0 | 99 | 0.847826 | SSC5 | DTC4 | 0 | 46 | 39 | 0 | 2005 | 2005 |
|  | 2007 | 2012 | 1 | India | 0 | 99 | 0.5625 | SSC5 | DTC4 | 0 | 32 | 18 | 0 | 2007 | 2007 |
|  | 2004 | 2012 | 1 | India | 0 | 99 | 0.033149 | SSC5 | DTC4 | 0 | 181 | 6 | 0 | 2004 | 2004 |
|  | 2005 | 2012 | 1 | India | 0 | 99 | 0.081967 | SSC5 | DTC4 | 0 | 122 | 10 | 0 | 2005 | 2005 |
|  | 2006 | 2012 | 1 | India | 0 | 99 | 0.041096 | SSC5 | DTC4 | 0 | 73 | 3 | 0 | 2006 | 2006 |
|  | 2007 | 2012 | 1 | India | 0 | 99 | 0.03125 | SSC5 | DTC4 | 0 | 32 | 1 | 0 | 2007 | 2007 |
|  | 2004 | 2012 | 1 | India | 0 | 99 | 0.102273 | SSC5 | DTC4 | 0 | 88 | 9 | 0 | 2004 | 2004 |
|  | 2005 | 2012 | 1 | India | 0 | 99 | 0.215686 | SSC5 | DTC4 | 0 | 51 | 11 | 0 | 2005 | 2005 |
|  | 2004 | 2012 | 1 | India | 0 | 99 | 0.221359 | SSC5 | DTC4 | 0 | 1030 | 228 | 0 | 2004 | 2004 |
|  | 2005 | 2012 | 1 | India | 0 | 99 | 0.247484 | SSC5 | DTC4 | 0 | 6061 | 1500 | 0 | 2005 | 2005 |
|  | 2006 | 2012 | 1 | India | 0 | 99 | 0.227586 | SSC5 | DTC4 | 0 | 2320 | 528 | 0 | 2006 | 2006 |
|  | 2007 | 2012 | 1 | India | 0 | 99 | 0.213294 | SSC5 | DTC4 | 0 | 3024 | 645 | 0 | 2007 | 2007 |
|  | 2008 | 2012 | 1 | India | 0 | 99 | 0.178287 | SSC5 | DTC4 | 1 | 3012 | 537 | 0 | 2008 | 2008 |
| (24) | 1973 | 2004 | 1 | India | 0 | 99 | 0.428571 | SSC1 | DTC4 | 0 | 700 | 300 | 0 | 1973 | 1973 |
|  | 1992 | 2004 | 1 | India | 0 | 99 | 0.461538 | SSC1 | DTC4 | 0 | 143 | 66 | 0 | 1992 | 1992 |
|  | 1993 | 2004 | 1 | India | 0 | 99 | 0.397447 | SSC1 | DTC4 | 0 | 1175 | 467 | 0 | 1993 | 1993 |
|  | 1994 | 2004 | 1 | India | 0 | 99 | 0.503846 | SSC1 | DTC4 | 0 | 260 | 131 | 0 | 1994 | 1994 |
|  | 1995 | 2004 | 1 | India | 0 | 99 | 0.273834 | SSC1 | DTC4 | 0 | 986 | 270 | 0 | 1995 | 1995 |
|  | 1996 | 2004 | 1 | India | 0 | 99 | 0.325301 | SSC1 | DTC4 | 0 | 332 | 108 | 0 | 1996 | 1996 |
|  | 1997 | 2004 | 1 | India | 0 | 99 | 0.251527 | SSC1 | DTC4 | 0 | 982 | 247 | 0 | 1997 | 1997 |
|  | 1998 | 2004 | 1 | India | 0 | 99 | 0.364326 | SSC1 | DTC4 | 0 | 527 | 192 | 0 | 1998 | 1998 |
|  | 1999 | 2004 | 1 | India | 0 | 99 | 0.207254 | SSC1 | DTC4 | 0 | 965 | 200 | 0 | 1999 | 1999 |
|  | 2000 | 2004 | 1 | India | 0 | 99 | 0.209913 | SSC1 | DTC4 | 0 | 343 | 72 | 0 | 2000 | 2000 |
|  | 1992 | 2004 | 1 | India | 0 | 99 | 0.359073 | SSC1 | DTC4 | 0 | 259 | 93 | 0 | 1992 | 1992 |
|  | 1993 | 2004 | 1 | India | 0 | 99 | 0.3125 | SSC1 | DTC4 | 0 | 96 | 30 | 0 | 1993 | 1993 |
|  | 1994 | 2004 | 1 | India | 0 | 99 | 0.630435 | SSC1 | DTC4 | 0 | 230 | 145 | 0 | 1994 | 1994 |
|  | 1996 | 2004 | 1 | India | 0 | 99 | 0.453125 | SSC1 | DTC4 | 0 | 64 | 29 | 0 | 1996 | 1996 |
|  | 1997 | 2004 | 1 | India | 0 | 99 | 0.295455 | SSC1 | DTC4 | 0 | 88 | 26 | 0 | 1997 | 1997 |
|  | 1998 | 2004 | 1 | India | 0 | 99 | 0.230769 | SSC1 | DTC4 | 0 | 26 | 6 | 0 | 1998 | 1998 |
|  | 2000 | 2004 | 1 | India | 0 | 99 | 0.436709 | SSC1 | DTC4 | 0 | 158 | 69 | 0 | 2000 | 2000 |
|  | 2001 | 2004 | 1 | India | 0 | 99 | 0.46888 | SSC1 | DTC4 | 0 | 241 | 113 | 0 | 2001 | 2001 |
|  | 2000 | 2004 | 1 | India | 0 | 99 | 0.246753 | SSC1 | DTC4 | 0 | 77 | 19 | 0 | 2000 | 2000 |
|  | 2001 | 2004 | 1 | India | 0 | 99 | 0.181818 | SSC1 | DTC4 | 0 | 88 | 16 | 0 | 2001 | 2001 |
|  | 1992 | 2004 | 1 | India | 0 | 99 | 0.292614 | SSC1 | DTC4 | 0 | 352 | 103 | 0 | 1992 | 1992 |
|  | 1993 | 2004 | 1 | India | 0 | 99 | 0.462264 | SSC1 | DTC4 | 0 | 106 | 49 | 0 | 1993 | 1993 |
|  | 1994 | 2004 | 1 | India | 0 | 99 | 0.574074 | SSC1 | DTC4 | 0 | 54 | 31 | 0 | 1994 | 1994 |
|  | 1992 | 2004 | 1 | India | 0 | 99 | 0.186047 | SSC1 | DTC4 | 0 | 43 | 8 | 0 | 1992 | 1992 |
|  | 1993 | 2004 | 1 | India | 0 | 99 | 0.27027 | SSC1 | DTC4 | 0 | 37 | 10 | 0 | 1993 | 1993 |
|  | 1997 | 2004 | 1 | India | 0 | 99 | 0.2 | SSC1 | DTC4 | 0 | 40 | 8 | 0 | 1997 | 1997 |
|  | 1992 | 2004 | 1 | India | 0 | 99 | 0.804878 | SSC1 | DTC4 | 0 | 41 | 33 | 0 | 1992 | 1992 |
|  | 1994 | 2004 | 1 | India | 0 | 99 | 0.848101 | SSC1 | DTC4 | 0 | 79 | 67 | 0 | 1994 | 1994 |
|  | 1995 | 2004 | 1 | India | 0 | 99 | 0.7 | SSC1 | DTC4 | 0 | 30 | 21 | 0 | 1995 | 1995 |
|  | 1996 | 2004 | 1 | India | 0 | 99 | 0.694915 | SSC1 | DTC4 | 0 | 59 | 41 | 0 | 1996 | 1996 |
|  | 1999 | 2004 | 1 | India | 0 | 99 | 0.46281 | SSC1 | DTC4 | 0 | 121 | 56 | 0 | 1999 | 1999 |
|  | 2000 | 2004 | 1 | India | 0 | 99 | 0.581081 | SSC1 | DTC4 | 0 | 74 | 43 | 0 | 2000 | 2000 |
|  | 2001 | 2004 | 1 | India | 0 | 99 | 0.488889 | SSC1 | DTC4 | 0 | 45 | 22 | 0 | 2001 | 2001 |
|  | 1992 | 2004 | 1 | India | 0 | 99 | 0.258621 | SSC1 | DTC4 | 0 | 58 | 15 | 0 | 1992 | 1992 |
|  | 1993 | 2004 | 1 | India | 0 | 99 | 0.222222 | SSC1 | DTC4 | 0 | 99 | 22 | 0 | 1993 | 1993 |
|  | 1994 | 2004 | 1 | India | 0 | 99 | 0.373016 | SSC1 | DTC4 | 0 | 126 | 47 | 0 | 1994 | 1994 |
|  | 1995 | 2004 | 1 | India | 0 | 99 | 0.312281 | SSC1 | DTC4 | 0 | 285 | 89 | 0 | 1995 | 1995 |
|  | 1996 | 2004 | 1 | India | 0 | 99 | 0.099415 | SSC1 | DTC4 | 0 | 171 | 17 | 0 | 1996 | 1996 |
|  | 1997 | 2004 | 1 | India | 0 | 99 | 0.199541 | SSC1 | DTC4 | 0 | 436 | 87 | 0 | 1997 | 1997 |
|  | 1998 | 2004 | 1 | India | 0 | 99 | 0.163399 | SSC1 | DTC4 | 0 | 306 | 50 | 0 | 1998 | 1998 |
|  | 1999 | 2004 | 1 | India | 0 | 99 | 0.147404 | SSC1 | DTC4 | 0 | 597 | 88 | 0 | 1999 | 1999 |
|  | 2000 | 2004 | 1 | India | 0 | 99 | 0.10274 | SSC1 | DTC4 | 0 | 438 | 45 | 0 | 2000 | 2000 |
|  | 2001 | 2004 | 1 | India | 0 | 99 | 0.082192 | SSC1 | DTC4 | 0 | 73 | 6 | 0 | 2001 | 2001 |
|  | 1996 | 2004 | 1 | India | 0 | 99 | 0.295238 | SSC1 | DTC4 | 0 | 105 | 31 | 0 | 1996 | 1996 |
|  | 1997 | 2004 | 1 | India | 0 | 99 | 0.157025 | SSC1 | DTC4 | 0 | 121 | 19 | 0 | 1997 | 1997 |
|  | 1998 | 2004 | 1 | India | 0 | 99 | 0.23301 | SSC1 | DTC4 | 0 | 103 | 24 | 0 | 1998 | 1998 |
|  | 1999 | 2004 | 1 | India | 0 | 99 | 0.018692 | SSC1 | DTC4 | 0 | 214 | 4 | 0 | 1999 | 1999 |
|  | 2000 | 2004 | 1 | India | 0 | 99 | 0.012195 | SSC1 | DTC4 | 0 | 164 | 2 | 0 | 2000 | 2000 |
|  | 1997 | 2004 | 1 | India | 0 | 99 | 0.069767 | SSC1 | DTC4 | 0 | 43 | 3 | 0 | 1997 | 1997 |
|  | 1995 | 2004 | 1 | India | 0 | 99 | 0.231343 | SSC1 | DTC4 | 0 | 268 | 62 | 0 | 1995 | 1995 |
|  | 1996 | 2004 | 1 | India | 0 | 99 | 0.1 | SSC1 | DTC4 | 0 | 20 | 2 | 0 | 1996 | 1996 |
|  | 1998 | 2004 | 1 | India | 0 | 99 | 0.035714 | SSC1 | DTC4 | 0 | 28 | 1 | 0 | 1998 | 1998 |
|  | 1999 | 2004 | 1 | India | 0 | 99 | 0.02381 | SSC1 | DTC4 | 0 | 42 | 1 | 0 | 1999 | 1999 |
|  | 1992 | 2004 | 1 | India | 0 | 99 | 0.60452 | SSC1 | DTC4 | 0 | 177 | 107 | 0 | 1992 | 1992 |
|  | 1993 | 2004 | 1 | India | 0 | 99 | 0.633094 | SSC1 | DTC4 | 0 | 278 | 176 | 0 | 1993 | 1993 |
|  | 1994 | 2004 | 1 | India | 0 | 99 | 0.523013 | SSC1 | DTC4 | 0 | 239 | 125 | 0 | 1994 | 1994 |
|  | 1995 | 2004 | 1 | India | 0 | 99 | 0.495652 | SSC1 | DTC4 | 0 | 115 | 57 | 0 | 1995 | 1995 |
|  | 1996 | 2004 | 1 | India | 0 | 99 | 0.477477 | SSC1 | DTC4 | 0 | 111 | 53 | 0 | 1996 | 1996 |
|  | 1997 | 2004 | 1 | India | 0 | 99 | 0.47191 | SSC1 | DTC4 | 0 | 89 | 42 | 0 | 1997 | 1997 |
|  | 1998 | 2004 | 1 | India | 0 | 99 | 0.56 | SSC1 | DTC4 | 0 | 25 | 14 | 0 | 1998 | 1998 |
|  | 1992 | 2004 | 1 | India | 0 | 99 | 0.288777 | SSC1 | DTC4 | 0 | 793 | 229 | 0 | 1992 | 1992 |
|  | 1993 | 2004 | 1 | India | 0 | 99 | 0.307692 | SSC1 | DTC4 | 0 | 104 | 32 | 0 | 1993 | 1993 |
|  | 1996 | 2004 | 1 | India | 0 | 99 | 0.239583 | SSC1 | DTC4 | 0 | 672 | 161 | 0 | 1996 | 1996 |
|  | 1997 | 2004 | 1 | India | 0 | 99 | 0.216524 | SSC1 | DTC4 | 0 | 351 | 76 | 0 | 1997 | 1997 |
|  | 1998 | 2004 | 1 | India | 0 | 99 | 0.185538 | SSC1 | DTC4 | 0 | 1051 | 195 | 0 | 1998 | 1998 |
|  | 1999 | 2004 | 1 | India | 0 | 99 | 0.20073 | SSC1 | DTC4 | 0 | 1370 | 275 | 0 | 1999 | 1999 |
|  | 2000 | 2004 | 1 | India | 0 | 99 | 0.216239 | SSC1 | DTC4 | 0 | 1170 | 253 | 0 | 2000 | 2000 |
|  | 2001 | 2004 | 1 | India | 0 | 99 | 0.2112 | SSC1 | DTC4 | 0 | 625 | 132 | 0 | 2001 | 2001 |
|  | 1992 | 2004 | 1 | India | 0 | 99 | 0.435754 | SSC1 | DTC4 | 0 | 537 | 234 | 0 | 1992 | 1992 |
|  | 1993 | 2004 | 1 | India | 0 | 99 | 0.336066 | SSC1 | DTC4 | 0 | 366 | 123 | 0 | 1993 | 1993 |
|  | 1994 | 2004 | 1 | India | 0 | 99 | 0.397436 | SSC1 | DTC4 | 0 | 234 | 93 | 0 | 1994 | 1994 |
|  | 1995 | 2004 | 1 | India | 0 | 99 | 0.33834 | SSC1 | DTC4 | 0 | 1265 | 428 | 0 | 1995 | 1995 |
|  | 1996 | 2004 | 1 | India | 0 | 99 | 0.213881 | SSC1 | DTC4 | 0 | 706 | 151 | 0 | 1996 | 1996 |
|  | 1997 | 2004 | 1 | India | 0 | 99 | 0.342541 | SSC1 | DTC4 | 0 | 362 | 124 | 0 | 1997 | 1997 |
|  | 1998 | 2004 | 1 | India | 0 | 99 | 0.25 | SSC1 | DTC4 | 0 | 36 | 9 | 0 | 1998 | 1998 |
|  | 1999 | 2004 | 1 | India | 0 | 99 | 0.229508 | SSC1 | DTC4 | 0 | 61 | 14 | 0 | 1999 | 1999 |
|  | 2000 | 2004 | 1 | India | 0 | 99 | 0.337838 | SSC1 | DTC4 | 0 | 148 | 50 | 0 | 2000 | 2000 |
|  | 2001 | 2004 | 1 | India | 0 | 99 | 0.14433 | SSC1 | DTC4 | 0 | 97 | 14 | 0 | 2001 | 2001 |
|  | 2002 | 2004 | 1 | India | 0 | 99 | 0.264023 | SSC1 | DTC4 | 0 | 1765 | 466 | 0 | 2002 | 2002 |
|  | 2003 | 2004 | 1 | India | 0 | 99 | 0.298223 | SSC1 | DTC4 | 0 | 2364 | 705 | 0 | 2003 | 2003 |
| (25) | 1988 | 1992 | 1 | India | 0 | 15 | 0.332571 | SSC3 | DTC1 | 0 | 875 | 291 | 0 | 1988 | 1988 |
| (26) | 1983 | 1985 | 1 | Thailand | 1 | 10 | 0.326531 | SSC3 | DTC3 | 0 | 49 | 16 | 0 | 1983 | 1983 |
| (27) | 2005 | 2008 | 3 | Nepal | 0 | 99 | 0.026866 | SSC2 | DTC3 | 0 | 335 | 9 | 0 | 2004 | 2006 |
| (28) | 1965 | 2008 | 1 | Japan | 0 | 99 | 0.265089 | SSC1 | DTC2 | 0 | 845 | 224 | 0 | 1965 | 1965 |
|  | 1966 | 2008 | 1 | Japan | 0 | 99 | 0.386872 | SSC1 | DTC2 | 0 | 2011 | 778 | 0 | 1966 | 1966 |
|  | 1967 | 2008 | 1 | Japan | 0 | 99 | 0.263907 | SSC1 | DTC2 | 0 | 773 | 204 | 0 | 1967 | 1967 |
|  | 1968 | 2008 | 1 | Japan | 0 | 99 | 0.582011 | SSC1 | DTC2 | 0 | 378 | 220 | 0 | 1968 | 1968 |
|  | 1969 | 2008 | 1 | Japan | 0 | 99 | 0.412162 | SSC1 | DTC2 | 0 | 148 | 61 | 0 | 1969 | 1969 |
|  | 1970 | 2008 | 1 | Japan | 0 | 99 | 0.386792 | SSC1 | DTC2 | 0 | 106 | 41 | 0 | 1970 | 1970 |
|  | 1971 | 2008 | 1 | Japan | 0 | 99 | 0.349057 | SSC1 | DTC2 | 0 | 106 | 37 | 0 | 1971 | 1971 |
|  | 1972 | 2008 | 1 | Japan | 0 | 99 | 0.375 | SSC1 | DTC2 | 0 | 24 | 9 | 0 | 1972 | 1972 |
|  | 1973 | 2008 | 1 | Japan | 0 | 99 | 0.371429 | SSC1 | DTC2 | 0 | 70 | 26 | 0 | 1973 | 1973 |
|  | 1975 | 2008 | 1 | Japan | 0 | 99 | 0.153846 | SSC1 | DTC2 | 0 | 26 | 4 | 0 | 1975 | 1975 |
|  | 1978 | 2008 | 1 | Japan | 0 | 99 | 0.252874 | SSC1 | DTC2 | 0 | 87 | 22 | 0 | 1978 | 1978 |
|  | 1979 | 2008 | 1 | Japan | 0 | 99 | 0.305882 | SSC1 | DTC2 | 0 | 85 | 26 | 0 | 1979 | 1979 |
|  | 1980 | 2008 | 1 | Japan | 0 | 99 | 0.375 | SSC1 | DTC2 | 0 | 40 | 15 | 0 | 1980 | 1980 |
|  | 1981 | 2008 | 1 | Japan | 0 | 99 | 0.217391 | SSC1 | DTC2 | 0 | 23 | 5 | 0 | 1981 | 1981 |
|  | 1982 | 2008 | 1 | Japan | 0 | 99 | 0.190476 | SSC1 | DTC2 | 0 | 21 | 4 | 0 | 1982 | 1982 |
|  | 1983 | 2008 | 1 | Japan | 0 | 99 | 0.25 | SSC1 | DTC2 | 0 | 32 | 8 | 0 | 1983 | 1983 |
|  | 1984 | 2008 | 1 | Japan | 0 | 99 | 0.185185 | SSC1 | DTC2 | 0 | 27 | 5 | 0 | 1984 | 1984 |
|  | 1985 | 2008 | 1 | Japan | 0 | 99 | 0.205128 | SSC1 | DTC2 | 0 | 39 | 8 | 0 | 1985 | 1985 |
|  | 1986 | 2008 | 1 | Japan | 0 | 99 | 0.115385 | SSC1 | DTC2 | 0 | 26 | 3 | 0 | 1986 | 1986 |
|  | 1987 | 2008 | 1 | Japan | 0 | 99 | 0.189189 | SSC1 | DTC2 | 0 | 37 | 7 | 0 | 1987 | 1987 |
|  | 1988 | 2008 | 1 | Japan | 0 | 99 | 0.125 | SSC1 | DTC2 | 0 | 32 | 4 | 0 | 1988 | 1988 |
|  | 1989 | 2008 | 1 | Japan | 0 | 99 | 0.148148 | SSC1 | DTC2 | 0 | 27 | 4 | 0 | 1989 | 1989 |
|  | 1990 | 2008 | 1 | Japan | 0 | 99 | 0.148148 | SSC1 | DTC2 | 0 | 54 | 8 | 0 | 1990 | 1990 |
|  | 1993 | 2008 | 23 | Japan | 0 | 99 | 0.160665 | SSC1 | DTC2 | 0 | 361 | 58 | 0 | 1982 | 2004 |
| (29) | 2010 | 2013 | 1 | China | 0 | 17 | 0.091954 | SSC3 | DTC3 | 1 | 87 | 8 | 0 | 2010 | 2010 |
| (30) | 1991 | 1995 | 2 | India | 0 | 99 | 0.090909 | SSC1 | DTC1 | 0 | 44 | 4 | 0 | 1990 | 1991 |
| (31) | 2006 | 2009 | 3 | India | 0 | 99 | 0.248366 | SSC3 | DTC3 | 0 | 153 | 38 | 0 | 2005 | 2007 |
| (32) | 2005 | 2016 | 1 | China | 0 | 99 | 0.030075 | SSC1 | DTC4 | 1 | 5320 | 160 | 0 | 2005 | 2005 |
|  | 2006 | 2016 | 1 | China | 0 | 99 | 0.072799 | SSC1 | DTC4 | 1 | 5316 | 387 | 0 | 2006 | 2006 |
|  | 2007 | 2016 | 1 | China | 0 | 99 | 0.035408 | SSC1 | DTC4 | 1 | 4660 | 165 | 0 | 2007 | 2007 |
|  | 2008 | 2016 | 1 | China | 0 | 99 | 0.041705 | SSC1 | DTC4 | 1 | 3237 | 135 | 0 | 2008 | 2008 |
|  | 2009 | 2016 | 1 | China | 0 | 99 | 0.040368 | SSC1 | DTC4 | 1 | 4236 | 171 | 0 | 2009 | 2009 |
|  | 2010 | 2016 | 1 | China | 0 | 99 | 0.034697 | SSC1 | DTC4 | 1 | 2738 | 95 | 0 | 2010 | 2010 |
|  | 2011 | 2016 | 1 | China | 0 | 99 | 0.038686 | SSC1 | DTC4 | 1 | 1887 | 73 | 0 | 2011 | 2011 |
|  | 2012 | 2016 | 1 | China | 0 | 99 | 0.034643 | SSC1 | DTC4 | 1 | 1934 | 67 | 0 | 2012 | 2012 |
|  | 2013 | 2016 | 1 | China | 0 | 99 | 0.03319 | SSC1 | DTC4 | 1 | 2320 | 77 | 0 | 2013 | 2013 |
|  | 2014 | 2016 | 1 | China | 0 | 99 | 0.034483 | SSC1 | DTC4 | 1 | 1044 | 36 | 0 | 2014 | 2014 |
| (33) | 1987 | 1990 | 4 | India | 0 | 12 | 0.369565 | SSC3 | DTC3 | 0 | 92 | 34 | 0 | 1985 | 1988 |
| (34) | 1995 | 2001 | 9 | India | 2 | 57 | 0.123077 | SSC5 | DTC3 | 0 | 65 | 8 | 0 | 1991 | 1999 |
| (35) | 2009 | 2017 | 1 | India | 0 | 99 | 0.122807 | SSC1 | DTC3 | 1 | 57 | 7 | 0 | 2009 | 2009 |
|  | 2010 | 2017 | 1 | India | 0 | 99 | 0.254237 | SSC1 | DTC3 | 1 | 59 | 15 | 0 | 2010 | 2010 |
|  | 2011 | 2017 | 1 | India | 0 | 99 | 0.136842 | SSC1 | DTC3 | 1 | 190 | 26 | 0 | 2011 | 2011 |
|  | 2012 | 2017 | 1 | India | 0 | 99 | 0.04878 | SSC1 | DTC3 | 1 | 41 | 2 | 0 | 2012 | 2012 |
|  | 2013 | 2017 | 1 | India | 0 | 99 | 0.103448 | SSC1 | DTC3 | 1 | 29 | 3 | 0 | 2013 | 2013 |
|  | 2014 | 2017 | 1 | India | 0 | 99 | 0.15 | SSC1 | DTC3 | 1 | 20 | 3 | 0 | 2014 | 2014 |
| (36) | 2008 | 2018 | 1 | India | 0 | 99 | 0.244444 | SSC3 | DTC3 | 1 | 45 | 11 | 1 | 2008 | 2008 |
| (37) | 1993 | 2002 | 13 | Thailand | 0 | 99 | 0.223529 | SSC3 | DTC3 | 0 | 85 | 19 | 0 | 1987 | 1999 |
| (38) | 2010 | 2012 | 3 | India | 0 | 99 | 0.166667 | SSC3 | DTC3 | 1 | 138 | 23 | 0 | 2009 | 2011 |
| (39) | 1998 | 2002 | 1 | India | 3 | 12 | 0.086957 | SSC3 | DTC3 | 0 | 23 | 2 | 0 | 1998 | 1998 |
| (40) | 2001 | 2006 | 2 | Nepal | 0 | 14 | 0.068966 | SSC3 | DTC3 | 0 | 58 | 4 | 0 | 2000 | 2001 |
| (41) | 2006 | 2008 | 2 | Indonesia | 0 | 15 | 0.158537 | SSC2 | DTC3 | 0 | 82 | 13 | 0 | 2005 | 2006 |
| (42) | 1991 | 1996 | 1 | India | 3 | 40 | 0.363636 | SSC1 | DTC1 | 0 | 33 | 12 | 0 | 1991 | 1991 |
| (43) | 2005 | 2006 | 1 | India | 0 | 12 | 0.337662 | SSC4 | DTC3 | 0 | 77 | 26 | 0 | 2005 | 2005 |
| (44) | 1991 | 1995 | 2 | India | 0 | 57 | 0.313559 | SSC1 | DTC1 | 0 | 118 | 37 | 0 | 1990 | 1991 |
| (45) | 1984 | 1992 | 1 | Thailand | 0 | 48 | 0.254545 | SSC3 | DTC3 | 0 | 55 | 14 | 0 | 1984 | 1984 |
| (46) | 2012 | 2014 | 2 | India | 0 | 99 | 0.298969 | SSC3 | DTC3 | 1 | 194 | 58 | 0 | 2011 | 2012 |
| (47) | 2012 | 2014 | 2 | India | 0 | 99 | 0.152344 | SSC5 | DTC3 | 1 | 256 | 39 | 0 | 2011 | 2012 |
| (48) | 2007 | 2009 | 3 | Cambodia | 0 | 15 | 0.096774 | SSC1 | DTC3 | 0 | 62 | 6 | 0 | 2006 | 2008 |
| (49) | 2004 | 2010 | 3 | Bangladesh | 0 | 55 | 0.1 | SSC2 | DTC2 | 0 | 20 | 2 | 0 | 2003 | 2005 |
| (50) | 1998 | 2003 | 4 | Vietnam | 1 | 15 | 0.205882 | SSC4 | DTC3 | 0 | 102 | 21 | 0 | 1996 | 1999 |
| (51) | 2009 | 2016 | 11 | India | 1 | 78 | 0.195876 | SSC3 | DTC3 | 1 | 97 | 19 | 0 | 2004 | 2014 |
|  | 1991 | 2015 | 1 | China | 0 | 99 | 0.088436 | SSC5 | DTC4 | 0 | 37903 | 3352 | 0 | 1991 | 1991 |
|  | 1992 | 2015 | 1 | China | 0 | 99 | 0.046472 | SSC5 | DTC4 | 0 | 23670 | 1100 | 0 | 1992 | 1992 |
|  | 1993 | 2015 | 1 | China | 0 | 99 | 0.042441 | SSC5 | DTC4 | 0 | 20075 | 852 | 0 | 1993 | 1993 |
|  | 1994 | 2015 | 1 | China | 0 | 99 | 0.040415 | SSC5 | DTC4 | 0 | 16479 | 666 | 0 | 1994 | 1994 |
|  | 1995 | 2015 | 1 | China | 0 | 99 | 0.040438 | SSC5 | DTC4 | 0 | 17978 | 727 | 0 | 1995 | 1995 |
|  | 1996 | 2015 | 1 | China | 0 | 99 | 0.034792 | SSC5 | DTC4 | 0 | 14831 | 516 | 0 | 1996 | 1996 |
|  | 1997 | 2015 | 1 | China | 0 | 99 | 0.03216 | SSC5 | DTC4 | 0 | 9888 | 318 | 0 | 1997 | 1997 |
|  | 1998 | 2015 | 1 | China | 0 | 99 | 0.037116 | SSC5 | DTC4 | 0 | 9888 | 367 | 0 | 1998 | 1998 |
|  | 1999 | 2015 | 1 | China | 0 | 99 | 0.035711 | SSC5 | DTC4 | 0 | 11985 | 428 | 0 | 1999 | 1999 |
|  | 2000 | 2015 | 1 | China | 0 | 99 | 0.04284 | SSC5 | DTC4 | 0 | 8240 | 353 | 0 | 2000 | 2000 |
|  | 2006 | 2015 | 1 | China | 0 | 99 | 0.045394 | SSC5 | DTC4 | 1 | 5243 | 238 | 0 | 2006 | 2006 |
|  | 2007 | 2015 | 1 | China | 0 | 99 | 0.069684 | SSC5 | DTC4 | 1 | 7491 | 522 | 0 | 2007 | 2007 |
|  | 2008 | 2015 | 1 | China | 0 | 99 | 0.055407 | SSC5 | DTC4 | 1 | 4494 | 249 | 0 | 2008 | 2008 |
|  | 2009 | 2015 | 1 | China | 0 | 99 | 0.043547 | SSC5 | DTC4 | 1 | 3146 | 137 | 0 | 2009 | 2009 |
|  | 2010 | 2015 | 1 | China | 0 | 99 | 0.045983 | SSC5 | DTC4 | 1 | 4045 | 186 | 0 | 2010 | 2010 |
|  | 2011 | 2015 | 1 | China | 0 | 99 | 0.034158 | SSC5 | DTC4 | 1 | 2547 | 87 | 0 | 2011 | 2011 |
|  | 1991 | 2015 | 1 | India | 0 | 99 | 0.379378 | SSC5 | DTC4 | 0 | 4054 | 1538 | 0 | 1991 | 1991 |
|  | 1992 | 2015 | 1 | India | 0 | 99 | 0.368794 | SSC5 | DTC4 | 0 | 2397 | 884 | 0 | 1992 | 1992 |
|  | 1993 | 2015 | 1 | India | 0 | 99 | 0.407965 | SSC5 | DTC4 | 0 | 2260 | 922 | 0 | 1993 | 1993 |
|  | 1994 | 2015 | 1 | India | 0 | 99 | 0.527663 | SSC5 | DTC4 | 0 | 1211 | 639 | 0 | 1994 | 1994 |
|  | 1995 | 2015 | 1 | India | 0 | 99 | 0.311525 | SSC5 | DTC4 | 0 | 1987 | 619 | 0 | 1995 | 1995 |
|  | 1997 | 2015 | 1 | India | 0 | 99 | 0.254072 | SSC5 | DTC4 | 0 | 2456 | 624 | 0 | 1997 | 1997 |
|  | 1998 | 2015 | 1 | India | 0 | 99 | 0.237128 | SSC5 | DTC4 | 0 | 2117 | 502 | 0 | 1998 | 1998 |
|  | 1999 | 2015 | 1 | India | 0 | 99 | 0.198294 | SSC5 | DTC4 | 0 | 3399 | 674 | 0 | 1999 | 1999 |
|  | 2000 | 2015 | 1 | India | 0 | 99 | 0.216301 | SSC5 | DTC4 | 0 | 2552 | 552 | 0 | 2000 | 2000 |
|  | 2001 | 2015 | 1 | India | 0 | 99 | 0.235821 | SSC5 | DTC4 | 0 | 2010 | 474 | 0 | 2001 | 2001 |
|  | 2002 | 2015 | 1 | India | 0 | 99 | 0.266432 | SSC5 | DTC4 | 0 | 1704 | 454 | 0 | 2002 | 2002 |
|  | 2003 | 2015 | 1 | India | 0 | 99 | 0.273655 | SSC5 | DTC4 | 0 | 2547 | 697 | 0 | 2003 | 2003 |
|  | 2004 | 2015 | 1 | India | 0 | 99 | 0.218616 | SSC5 | DTC4 | 0 | 1633 | 357 | 0 | 2004 | 2004 |
|  | 2005 | 2015 | 1 | India | 0 | 99 | 0.250675 | SSC5 | DTC4 | 0 | 6666 | 1671 | 0 | 2005 | 2005 |
|  | 2006 | 2015 | 1 | India | 0 | 99 | 0.234608 | SSC5 | DTC4 | 0 | 2745 | 644 | 0 | 2006 | 2006 |
|  | 2007 | 2015 | 1 | India | 0 | 99 | 0.242231 | SSC5 | DTC4 | 0 | 3926 | 951 | 0 | 2007 | 2007 |
|  | 2008 | 2015 | 1 | India | 0 | 99 | 0.178162 | SSC5 | DTC4 | 1 | 3755 | 669 | 0 | 2008 | 2008 |
|  | 2009 | 2015 | 1 | India | 0 | 99 | 0.172469 | SSC5 | DTC4 | 1 | 4395 | 758 | 0 | 2009 | 2009 |
|  | 2010 | 2015 | 1 | India | 0 | 99 | 0.132079 | SSC5 | DTC4 | 1 | 5103 | 674 | 0 | 2010 | 2010 |
|  | 2011 | 2015 | 1 | India | 0 | 99 | 0.009418 | SSC5 | DTC4 | 1 | 8176 | 77 | 0 | 2011 | 2011 |
|  | 1984 | 2015 | 1 | Japan | 0 | 99 | 0.259259 | SSC5 | DTC4 | 0 | 27 | 7 | 0 | 1984 | 1984 |
|  | 1985 | 2015 | 1 | Japan | 0 | 99 | 0.205128 | SSC5 | DTC4 | 0 | 39 | 8 | 0 | 1985 | 1985 |
|  | 1986 | 2015 | 1 | Japan | 0 | 99 | 0.115385 | SSC5 | DTC4 | 0 | 26 | 3 | 0 | 1986 | 1986 |
|  | 1987 | 2015 | 1 | Japan | 0 | 99 | 0.189189 | SSC5 | DTC4 | 0 | 37 | 7 | 0 | 1987 | 1987 |
|  | 1988 | 2015 | 1 | Japan | 0 | 99 | 0.125 | SSC5 | DTC4 | 0 | 32 | 4 | 0 | 1988 | 1988 |
|  | 1989 | 2015 | 1 | Japan | 0 | 99 | 0.148148 | SSC5 | DTC4 | 0 | 27 | 4 | 0 | 1989 | 1989 |
|  | 1990 | 2015 | 1 | Japan | 0 | 99 | 0.148148 | SSC5 | DTC4 | 0 | 54 | 8 | 0 | 1990 | 1990 |
|  | 1983 | 2015 | 1 | Korea | 0 | 99 | 0.108696 | SSC5 | DTC4 | 0 | 138 | 15 | 0 | 1983 | 1983 |
|  | 2010 | 2015 | 1 | Korea | 0 | 99 | 0.269231 | SSC5 | DTC4 | 1 | 26 | 7 | 0 | 2010 | 2010 |
|  | 1995 | 2015 | 1 | Thailand | 0 | 99 | 0.121818 | SSC5 | DTC4 | 0 | 550 | 67 | 0 | 1995 | 1995 |
|  | 1996 | 2015 | 1 | Thailand | 0 | 99 | 0.099792 | SSC5 | DTC4 | 0 | 481 | 48 | 0 | 1996 | 1996 |
|  | 1997 | 2015 | 1 | Thailand | 0 | 99 | 0.114504 | SSC5 | DTC4 | 0 | 524 | 60 | 0 | 1997 | 1997 |
|  | 1998 | 2015 | 1 | Thailand | 0 | 99 | 0.075404 | SSC5 | DTC4 | 0 | 557 | 42 | 0 | 1998 | 1998 |
|  | 1999 | 2015 | 1 | Thailand | 0 | 99 | 0.123426 | SSC5 | DTC4 | 0 | 397 | 49 | 0 | 1999 | 1999 |
|  | 2000 | 2015 | 1 | Thailand | 0 | 99 | 0.10049 | SSC5 | DTC4 | 0 | 408 | 41 | 0 | 2000 | 2000 |
|  | 2001 | 2015 | 1 | Thailand | 0 | 99 | 0.070529 | SSC5 | DTC4 | 0 | 397 | 28 | 0 | 2001 | 2001 |
|  | 2002 | 2015 | 1 | Thailand | 0 | 99 | 0.104396 | SSC5 | DTC4 | 0 | 364 | 38 | 0 | 2002 | 2002 |
|  | 2003 | 2015 | 1 | Thailand | 0 | 99 | 0.137184 | SSC5 | DTC4 | 1 | 277 | 38 | 0 | 2003 | 2003 |
|  | 2004 | 2015 | 1 | Thailand | 0 | 99 | 0.030361 | SSC5 | DTC4 | 1 | 527 | 16 | 0 | 2004 | 2004 |
|  | 2005 | 2015 | 1 | Thailand | 0 | 99 | 0.039755 | SSC5 | DTC4 | 1 | 327 | 13 | 0 | 2005 | 2005 |
|  | 2007 | 2015 | 1 | Thailand | 0 | 99 | 0.005348 | SSC5 | DTC4 | 1 | 374 | 2 | 0 | 2007 | 2007 |
|  | 2008 | 2015 | 1 | Thailand | 0 | 99 | 0.004831 | SSC5 | DTC4 | 1 | 414 | 2 | 0 | 2008 | 2008 |
|  | 2009 | 2015 | 1 | Thailand | 0 | 99 | 0.005017 | SSC5 | DTC4 | 1 | 598 | 3 | 0 | 2009 | 2009 |
|  | 1991 | 2015 | 1 | Vietnam | 0 | 99 | 0.046193 | SSC5 | DTC4 | 0 | 1970 | 91 | 0 | 1991 | 1991 |
|  | 1992 | 2015 | 1 | Vietnam | 0 | 99 | 0.047157 | SSC5 | DTC4 | 0 | 2269 | 107 | 0 | 1992 | 1992 |
|  | 1993 | 2015 | 1 | Vietnam | 0 | 99 | 0.049129 | SSC5 | DTC4 | 0 | 2239 | 110 | 0 | 1993 | 1993 |
|  | 1994 | 2015 | 1 | Vietnam | 0 | 99 | 0.040374 | SSC5 | DTC4 | 0 | 3418 | 138 | 0 | 1994 | 1994 |
|  | 1995 | 2015 | 1 | Vietnam | 0 | 99 | 0.047724 | SSC5 | DTC4 | 0 | 3164 | 151 | 0 | 1995 | 1995 |
|  | 1996 | 2015 | 1 | Vietnam | 0 | 99 | 0.040398 | SSC5 | DTC4 | 0 | 3119 | 126 | 0 | 1996 | 1996 |
|  | 1997 | 2015 | 1 | Vietnam | 0 | 99 | 0.025173 | SSC5 | DTC4 | 0 | 2463 | 62 | 0 | 1997 | 1997 |
|  | 1998 | 2015 | 1 | Vietnam | 0 | 99 | 0.034817 | SSC5 | DTC4 | 0 | 2269 | 79 | 0 | 1998 | 1998 |
|  | 1999 | 2015 | 1 | Vietnam | 0 | 99 | 0.05433 | SSC5 | DTC4 | 0 | 2448 | 133 | 0 | 1999 | 1999 |
|  | 2000 | 2015 | 1 | Vietnam | 0 | 99 | 0.007246 | SSC5 | DTC4 | 0 | 552 | 4 | 0 | 2000 | 2000 |
|  | 2004 | 2015 | 1 | Vietnam | 0 | 99 | 0.056701 | SSC5 | DTC4 | 0 | 194 | 11 | 0 | 2004 | 2004 |
|  | 2010 | 2015 | 1 | Vietnam | 0 | 99 | 0.092437 | SSC5 | DTC4 | 0 | 119 | 11 | 0 | 2010 | 2010 |
| (52) | 2006 | 2012 | 1 | China | 0 | 99 | 0.287879 | SSC5 | DTC3 | 1 | 66 | 19 | 0 | 2006 | 2006 |
|  | 2001 | 2012 | 1 | China | 0 | 99 | 0.025115 | SSC5 | DTC4 | 0 | 9795 | 246 | 0 | 2001 | 2001 |
|  | 2002 | 2012 | 1 | China | 0 | 99 | 0.026115 | SSC5 | DTC4 | 0 | 8769 | 229 | 0 | 2002 | 2002 |
|  | 2003 | 2012 | 1 | China | 0 | 99 | 0.046565 | SSC5 | DTC4 | 0 | 7860 | 366 | 0 | 2003 | 2003 |
|  | 2004 | 2012 | 1 | China | 0 | 99 | 0.036887 | SSC5 | DTC4 | 1 | 5422 | 200 | 0 | 2004 | 2004 |
|  | 2005 | 2012 | 1 | China | 0 | 99 | 0.041985 | SSC5 | DTC4 | 1 | 5097 | 214 | 0 | 2005 | 2005 |
|  | 2006 | 2012 | 1 | China | 0 | 99 | 0.060578 | SSC5 | DTC4 | 1 | 7643 | 463 | 0 | 2006 | 2006 |
|  | 2007 | 2012 | 1 | China | 0 | 99 | 0.052425 | SSC5 | DTC4 | 1 | 4330 | 227 | 0 | 2007 | 2007 |
|  | 2008 | 2012 | 1 | China | 0 | 99 | 0.047731 | SSC5 | DTC4 | 1 | 2975 | 142 | 0 | 2008 | 2008 |
|  | 2009 | 2012 | 1 | China | 0 | 99 | 0.043956 | SSC5 | DTC4 | 1 | 3913 | 172 | 0 | 2009 | 2009 |
|  | 2010 | 2012 | 1 | China | 0 | 99 | 0.036206 | SSC5 | DTC4 | 1 | 2541 | 92 | 0 | 2010 | 2010 |
|  | 2006 | 2012 | 10 | China | 0 | 99 | 0.040295 | SSC5 | DTC4 | 1 | 58345 | 2351 | 0 | 2001 | 2010 |
| (53) | 1985 | 2005 | 37 | Hong Kong | 0 | 99 | 0.111111 | SSC5 | DTC4 | 0 | 45 | 5 | 0 | 1967 | 2003 |
| (54) | 2004 | 2010 | 3 | Thailand | 0 | 57 | 0.045455 | SSC1 | DTC1 | 1 | 22 | 1 | 0 | 2003 | 2005 |
| (55) | 1987 | 1989 | 2 | India | 0 | 15 | 0.5 | SSC3 | DTC1 | 0 | 22 | 11 | 0 | 1986 | 1987 |
| (56) | 2008 | 2015 | 1 | India | 0 | 99 | 0.210191 | SSC1 | DTC3 | 1 | 157 | 33 | 0 | 2008 | 2008 |
|  | 2009 | 2015 | 1 | India | 0 | 99 | 0.211009 | SSC1 | DTC3 | 1 | 218 | 46 | 0 | 2009 | 2009 |
|  | 2010 | 2015 | 1 | India | 0 | 99 | 0.266234 | SSC1 | DTC3 | 1 | 154 | 41 | 0 | 2010 | 2010 |
|  | 2011 | 2015 | 1 | India | 0 | 99 | 0.213884 | SSC1 | DTC3 | 1 | 533 | 114 | 0 | 2011 | 2011 |
|  | 2012 | 2015 | 1 | India | 0 | 99 | 0.220165 | SSC1 | DTC3 | 1 | 486 | 107 | 0 | 2012 | 2012 |
|  | 2013 | 2015 | 1 | India | 0 | 99 | 0.270161 | SSC1 | DTC3 | 1 | 496 | 134 | 0 | 2013 | 2013 |
| (57) | 2006 | 2009 | 2 | Indonesia | 0 | 15 | 0.152778 | SSC2 | DTC3 | 0 | 72 | 11 | 0 | 2005 | 2006 |
| (58) | 1973 | 2008 | 1 | India | 0 | 99 | 0.428571 | SSC5 | DTC4 | 0 | 700 | 300 | 1 | 1973 | 1973 |
|  | 1976 | 2008 | 1 | India | 0 | 99 | 0.410423 | SSC5 | DTC4 | 0 | 307 | 126 | 1 | 1976 | 1976 |
|  | 1999 | 2008 | 1 | India | 0 | 99 | 0.203895 | SSC5 | DTC4 | 0 | 873 | 178 | 1 | 1999 | 1999 |
| (59) | 1997 | 2002 | 4 | Vietnam | 0 | 14 | 0.119403 | SSC3 | DTC3 | 0 | 134 | 16 | 0 | 1995 | 1998 |
| (60) | 1996 | 2012 | 3 | India | 0 | 11 | 0.265 | SSC4 | DTC3 | 0 | 200 | 53 | 0 | 1995 | 1997 |
| (61) | 1978 | 2012 | 1 | India | 0 | 99 | 0.313416 | SSC5 | DTC3 | 0 | 3548 | 1112 | 0 | 1978 | 1978 |
|  | 1979 | 2012 | 1 | India | 0 | 99 | 0.5 | SSC5 | DTC3 | 0 | 134 | 67 | 0 | 1979 | 1979 |
|  | 1980 | 2012 | 1 | India | 0 | 99 | 0.327273 | SSC5 | DTC3 | 0 | 1595 | 522 | 0 | 1980 | 1980 |
|  | 1981 | 2012 | 1 | India | 0 | 99 | 0.491525 | SSC5 | DTC3 | 0 | 59 | 29 | 0 | 1981 | 1981 |
|  | 1982 | 2012 | 1 | India | 0 | 99 | 0.312298 | SSC5 | DTC3 | 0 | 618 | 193 | 0 | 1982 | 1982 |
|  | 1983 | 2012 | 1 | India | 0 | 99 | 0.364286 | SSC5 | DTC3 | 0 | 140 | 51 | 0 | 1983 | 1983 |
|  | 1985 | 2012 | 1 | India | 0 | 99 | 0.340986 | SSC5 | DTC3 | 0 | 1176 | 401 | 0 | 1985 | 1985 |
|  | 1986 | 2012 | 1 | India | 0 | 99 | 0.345609 | SSC5 | DTC3 | 0 | 1765 | 610 | 0 | 1986 | 1986 |
|  | 1987 | 2012 | 1 | India | 0 | 99 | 0.426036 | SSC5 | DTC3 | 0 | 169 | 72 | 0 | 1987 | 1987 |
|  | 1988 | 2012 | 1 | India | 0 | 99 | 0.313199 | SSC5 | DTC3 | 0 | 4470 | 1400 | 0 | 1988 | 1988 |
|  | 1989 | 2012 | 1 | India | 0 | 99 | 0.34229 | SSC5 | DTC3 | 0 | 1563 | 535 | 0 | 1989 | 1989 |
|  | 1990 | 2012 | 1 | India | 0 | 99 | 0.323864 | SSC5 | DTC3 | 0 | 176 | 57 | 0 | 1990 | 1990 |
|  | 1991 | 2012 | 1 | India | 0 | 99 | 0.341799 | SSC5 | DTC3 | 0 | 1890 | 646 | 0 | 1991 | 1991 |
|  | 1992 | 2012 | 1 | India | 0 | 99 | 0.270648 | SSC5 | DTC3 | 0 | 787 | 213 | 0 | 1992 | 1992 |
|  | 1993 | 2012 | 1 | India | 0 | 99 | 0.188119 | SSC5 | DTC3 | 0 | 101 | 19 | 0 | 1993 | 1993 |
|  | 1994 | 2012 | 1 | India | 0 | 99 | 0.124138 | SSC5 | DTC3 | 0 | 145 | 18 | 0 | 1994 | 1994 |
|  | 1995 | 2012 | 1 | India | 0 | 99 | 0.20625 | SSC5 | DTC3 | 0 | 160 | 33 | 0 | 1995 | 1995 |
|  | 1996 | 2012 | 1 | India | 0 | 99 | 0.230653 | SSC5 | DTC3 | 0 | 659 | 152 | 0 | 1996 | 1996 |
|  | 1997 | 2012 | 1 | India | 0 | 99 | 0.207101 | SSC5 | DTC3 | 0 | 338 | 70 | 0 | 1997 | 1997 |
|  | 1998 | 2012 | 1 | India | 0 | 99 | 0.175168 | SSC5 | DTC3 | 0 | 1039 | 182 | 0 | 1998 | 1998 |
|  | 1999 | 2012 | 1 | India | 0 | 99 | 0.19426 | SSC5 | DTC3 | 0 | 1359 | 264 | 0 | 1999 | 1999 |
|  | 2000 | 2012 | 1 | India | 0 | 99 | 0.2 | SSC5 | DTC3 | 0 | 1165 | 233 | 0 | 2000 | 2000 |
|  | 2001 | 2012 | 1 | India | 0 | 99 | 0.182276 | SSC5 | DTC3 | 0 | 993 | 181 | 0 | 2001 | 2001 |
|  | 2002 | 2012 | 1 | India | 0 | 99 | 0.192893 | SSC5 | DTC3 | 0 | 591 | 114 | 0 | 2002 | 2002 |
|  | 2003 | 2012 | 1 | India | 0 | 99 | 0.202338 | SSC5 | DTC3 | 0 | 1112 | 225 | 0 | 2003 | 2003 |
|  | 2004 | 2012 | 1 | India | 0 | 99 | 0.206897 | SSC5 | DTC3 | 0 | 1015 | 210 | 0 | 2004 | 2004 |
|  | 2005 | 2012 | 1 | India | 0 | 99 | 0.245742 | SSC5 | DTC3 | 0 | 6047 | 1486 | 0 | 2005 | 2005 |
|  | 2006 | 2012 | 1 | India | 0 | 99 | 0.223958 | SSC5 | DTC3 | 0 | 2304 | 516 | 0 | 2006 | 2006 |
|  | 2007 | 2012 | 1 | India | 0 | 99 | 0.208652 | SSC5 | DTC3 | 0 | 3005 | 627 | 0 | 2007 | 2007 |
|  | 2008 | 2012 | 1 | India | 0 | 99 | 0.176844 | SSC5 | DTC3 | 1 | 2997 | 530 | 0 | 2008 | 2008 |
|  | 2009 | 2012 | 1 | India | 0 | 99 | 0.173345 | SSC5 | DTC3 | 1 | 3444 | 597 | 0 | 2009 | 2009 |
| (62) | 1991 | 1996 | 1 | China | 0 | 99 | 0.042857 | SSC5 | DTC4 | 0 | 70 | 3 | 0 | 1991 | 1991 |
| (63) | 2015 | 2016 | 1 | India | 0 | 14 | 0.1 | SSC4 | DTC3 | 1 | 20 | 2 | 1 | 2015 | 2015 |
| (64) | 1989 | 1991 | 1 | India | 6 | 99 | 0.365854 | SSC5 | DTC4 | 0 | 41 | 15 | 1 | 1989 | 1989 |
| (65) | 1997 | 1997 | 1 | Nepal | 0 | 99 | 0.126844 | SSC3 | DTC4 | 0 | 339 | 43 | 0 | 1997 | 1997 |
| (66) | 2010 | 2012 | 1 | Korea | 0 | 99 | 0.230769 | SSC1 | DTC2 | 1 | 26 | 6 | 0 | 2010 | 2010 |
| (67) | 1982 | 1986 | 1 | Korea | 15 | 24 | 0.277778 | SSC2 | DTC3 | 0 | 36 | 10 | 0 | 1982 | 1982 |
|  | 1982 | 1986 | 1 | Korea | 15 | 24 | 0.333333 | SSC2 | DTC3 | 0 | 60 | 20 | 0 | 1982 | 1982 |
| (68) | 1996 | 2009 | 1 | China | 0 | 99 | 0.036768 | SSC1 | DTC4 | 0 | 10308 | 379 | 0 | 1996 | 1996 |
|  | 1997 | 2009 | 1 | China | 0 | 99 | 0.036779 | SSC1 | DTC4 | 0 | 10060 | 370 | 0 | 1997 | 1997 |
|  | 1998 | 2009 | 1 | China | 0 | 99 | 0.040833 | SSC1 | DTC4 | 0 | 12490 | 510 | 0 | 1998 | 1998 |
|  | 1999 | 2009 | 1 | China | 0 | 99 | 0.040673 | SSC1 | DTC4 | 0 | 8556 | 348 | 0 | 1999 | 1999 |
|  | 2000 | 2009 | 1 | China | 0 | 99 | 0.031836 | SSC1 | DTC4 | 0 | 11779 | 375 | 0 | 2000 | 2000 |
|  | 2001 | 2009 | 1 | China | 0 | 99 | 0.025115 | SSC1 | DTC4 | 0 | 9795 | 246 | 0 | 2001 | 2001 |
|  | 2002 | 2009 | 1 | China | 0 | 99 | 0.026115 | SSC1 | DTC4 | 0 | 8769 | 229 | 0 | 2002 | 2002 |
|  | 2003 | 2009 | 1 | China | 0 | 99 | 0.046565 | SSC1 | DTC4 | 0 | 7860 | 366 | 0 | 2003 | 2003 |
|  | 2004 | 2009 | 1 | China | 0 | 99 | 0.036887 | SSC1 | DTC4 | 1 | 5422 | 200 | 0 | 2004 | 2004 |
|  | 2005 | 2009 | 1 | China | 0 | 99 | 0.041985 | SSC1 | DTC4 | 1 | 5097 | 214 | 0 | 2005 | 2005 |
| (69) | 2007 | 2010 | 3 | China | 0 | 99 | 0.032864 | SSC4 | DTC3 | 1 | 213 | 7 | 0 | 2006 | 2008 |
| (70) | 2001 | 2007 | 2 | Nepal | 0 | 99 | 0.083333 | SSC3 | DTC3 | 0 | 48 | 4 | 0 | 2000 | 2001 |
| (71) | 1984 | 2007 | 22 | China | 0 | 14 | 0.082353 | SSC2 | DTC3 | 0 | 85 | 7 | 0 | 1973 | 1994 |
| (72) | 2003 | 2010 | 10 | Vietnam | 0 | 99 | 0.121212 | SSC1 | DTC3 | 0 | 33 | 4 | 0 | 1998 | 2007 |
| (73) | 2005 | 2006 | 1 | India | 0 | 99 | 0.234269 | SSC5 | DTC4 | 0 | 5737 | 1344 | 1 | 2005 | 2005 |
| (74) | 1974 | 1977 | 1 | Myanmar | 0 | 99 | 0.761905 | SSC5 | DTC4 | 0 | 42 | 32 | 1 | 1974 | 1974 |
| (75) | 1999 | 2001 | 1 | Nepal | 0 | 99 | 0.148427 | SSC1 | DTC4 | 0 | 2924 | 434 | 0 | 1999 | 1999 |
| (76) | 1998 | 2004 | 1 | Nepal | 0 | 99 | 0.128338 | SSC2 | DTC4 | 0 | 1161 | 149 | 0 | 1998 | 1998 |
|  | 1999 | 2004 | 1 | Nepal | 0 | 99 | 0.148427 | SSC2 | DTC4 | 0 | 2924 | 434 | 0 | 1999 | 1999 |
|  | 2000 | 2004 | 1 | Nepal | 0 | 99 | 0.097744 | SSC2 | DTC4 | 0 | 1729 | 169 | 0 | 2000 | 2000 |
|  | 2001 | 2004 | 1 | Nepal | 0 | 99 | 0.145657 | SSC2 | DTC4 | 0 | 1888 | 275 | 0 | 2001 | 2001 |
|  | 2002 | 2004 | 1 | Nepal | 0 | 99 | 0.199525 | SSC2 | DTC4 | 0 | 842 | 168 | 0 | 2002 | 2002 |
|  | 2003 | 2004 | 1 | Nepal | 0 | 99 | 0.209091 | SSC2 | DTC4 | 0 | 330 | 69 | 0 | 2003 | 2003 |
| (77) | 2004 | 2005 | 1 | India | 0 | 99 | 0.226087 | SSC4 | DTC4 | 0 | 115 | 26 | 1 | 2004 | 2004 |
| (78) | 1986 | 1991 | 1 | India | 0 | 99 | 0.25 | SSC3 | DTC4 | 0 | 52 | 13 | 0 | 1986 | 1986 |
|  | 1987 | 1991 | 1 | India | 0 | 99 | 0.52381 | SSC3 | DTC4 | 0 | 21 | 11 | 0 | 1987 | 1987 |
|  | 1988 | 1991 | 1 | India | 0 | 99 | 0.384615 | SSC3 | DTC4 | 0 | 26 | 10 | 0 | 1988 | 1988 |
| (79) | 2010 | 2013 | 5 | India | 0 | 99 | 0.181818 | SSC3 | DTC3 | 1 | 66 | 12 | 0 | 2008 | 2012 |
| (80) | 2009 | 2020 | 16 | India | 0 | 99 | 0.370839 | SSC1 | DTC3 | 0 | 2764 | 1025 | 0 | 2001 | 2016 |

References

1. Akiba T, Osaka K, Tang S, Nakayama M, Yamamoto A, Kurane I, et al. Analysis of Japanese encephalitis epidemic in western Nepal in 1997. Epidemiol Infect. 2001;126(1):81–8.

2. Res M, Ruts C, Hospital CR, Sciences M, Committee IE, Crh-smims S. Incidence of Japanese Encephalitis amongst acute encephalitis syndrome cases in upper Assam districts from 2012 to 2014: A report from a tertiary care hospital. 2018;(May):517–20.

3. Mong HO, Lewthwaite P, Boon FL, Mohan A, Clear D, Lim L, et al. The epidemiology, clinical features, and long-term prognosis of Japanese encephalitis in central Sarawak, Malaysia, 1997-2005. Clin Infect Dis. 2008 Aug;47(4):458–68.

4. Kono R, Kim KH. Comparative epidemiological features of Japanese encephalitis in the Republic of Korea, China (Taiwan) and Japan. Bull World Health Organ. 1969;40(2):263–77.

5. Sahu SS, Dash S, Sonia T, Muthukumaravel S, Sankari T, Gunasekaran K, et al. Entomological investigation of Japanese encephalitis outbreak in Malkangiri district of Odisha state, India. Mem Inst Oswaldo Cruz. 2018;

6. Kumar Pant D, Tenzin T, Chand R, Kumar Sharma B, Raj Bist P. Spatio-temporal epidemiology of Japanese encephalitis in Nepal, 2007-2015. PLoS One. 2017;

7. Borah J, Dutta P, Khan SA, Mahanta J. A comparison of clinical features of Japanese encephalitis virus infection in the adult and pediatric age group with Acute Encephalitis Syndrome. J Clin Virol. 2011;52(1):45–9.

8. Tao Z, Liu G, Wang M, Wang H, Lin X, Song L, et al. Molecular epidemiology of japanese encephalitis virus in mosquitoes during an outbreak in China, 2013. Sci Rep. 2014;

9. Zhang L, Luan RS, Jiang F, Rui LP, Liu M, Li YX, et al. Epidemiological characteristics of Japanese encephalitis in Guizhou Province, China, 1971-2009. Biomed Environ Sci. 2012;

10. Hills SL, Van cuong N, Touch S, Mai HH, Soeung SC, Lien TTH, et al. Disability from Japanese encephalitis in Cambodia and Viet Nam. J Trop Pediatr. 2011 Aug;57(4):241–4.

11. Rayamajhi A, Ansari I, Ledger E, Bista KP, Impoinvil DE, Nightingale S, et al. Clinical and prognostic features among children with acute encephalitis syndrome in Nepal; a retrospective study. BMC Infect Dis. 2011;11(1):294.

12. Kari K, Liu W, Gautama K, Mammen MP, Clemens JD, Nisalak A, et al. A hospital-based surveillance for Japanese encephalitis in Bali, Indonesia. BMC Med. 2006;4:2–8.

13. Sohn YM. Japanese encephalitis immunization in South Korea: Past, present, and future. Emerging Infectious Diseases. 2000.

14. Bhatt GC, Sharma T. Comment on “clinical Profile and Outcome of Japanese Encephalitis in Children Admitted with Acute Encephalitis Syndrome.” Biomed Res Int. 2014;2014(December 2012).

15. van Tan L, Qui PT, Ha DQ, Hue NB, Bao LQ, van Cam B, et al. Viral etiology of encephalitis in children in Southern Vietnam: Results of a one-year prospective descriptive study. PLoS Negl Trop Dis. 2010;4(10).

16. Seo H-J, Kim HC, Klein TA, Ramey AM, Lee J-H, Kyung S-G, et al. Molecular Detection and Genotyping of Japanese Encephalitis Virus in Mosquitoes during a 2010 Outbreak in the Republic of Korea. Ooi EE, editor. PLoS One. 2013 Feb;8(2):e55165.

17. Chheng K, Carter MJ, Emary K, Chanpheaktra N, Moore CE, Stoesser N, et al. A Prospective Study of the Causes of Febrile Illness Requiring Hospitalization in Children in Cambodia. PLoS One. 2013;

18. Dumre SP, Shakya G, Na-Bangchang K, Eursitthichai V, Grams HR, Upreti SR, et al. Short report: Dengue virus and Japanese encephalitis virus epidemiological shifts in Nepal: A case of opposing trends. Am J Trop Med Hyg. 2013;

19. Partridge J, Ghimire P, Sedai T, Bista MB, Banerjee M. Endemic Japanese encephalitis in the Kathmandu valley, Nepal. Am J Trop Med Hyg. 2007;

20. Nyari N, Singh D, Kakkar K, Sharma S, Pandey SN, Dhole TN. Entomological and serological investigation of Japanese encephalitis in endemic area of eastern Uttar Pradesh, India. J Vector Borne Dis. 2015;

21. Sarkari NBS, Thacker AK, Barthwal SP, Mishra VK, Prapann S, Srivastava D, et al. Japanese encephalitis (JE). Part I: Clinical profile of 1,282 adult acute cases of four epidemics. J Neurol. 2012;259(1):47–57.

22. Singh Z, Agarwal VK. Japanese encephalitis: Is routine immunization required? Medical Journal Armed Forces India. 2005.

23. Tiwari S, Singh RK, Tiwari R, Dhole TN. Japanese encephalitis: A review of the Indian perspective. Brazilian J Infect Dis. 2012 Nov;16(6):564–73.

24. Kabilan L, Rajendran R, Arunachalam N, Ramesh S, Srinivasan S, Samuel PP, et al. Japanese encephalitis in India: An overview. Indian J Pediatr. 2004;71(7):609–15.

25. Rathi AK, Kushwaha KP, Singh YD, Singh J, Sirohi R, Singh RK, et al. JE virus encephalitis: 1988 epidemic at Gorakhpur. Indian Pediatr. 1993;

26. Johnson RT, Burke DS, Elwell M, Leake CJ, Nisalak A, Hoke CH, et al. Japanese encephalitis: Immunocytochemical studies of viral antigen and Inflammatory cells in fatal cases. Ann Neurol. 1985;

27. Wierzba TF, Ghimire P, Malla S, Banerjee MK, Shrestha S, Khanal B, et al. Laboratory-based Japanese encephalitis surveillance in Nepal and the implications for a national immunization strategy. Am J Trop Med Hyg. 2008;

28. Arai S, Matsunaga Y, Takasaki T, Tanaka-Taya K, Taniguchi K, Okabe N, et al. Japanese encephalitis: Surveillance and elimination effort in Japan from 1982 to 2004. Japanese Journal of Infectious Diseases. 2008.

29. Ma J, Jiang L. Outcome of children with japanese encephalitis and predictors of outcome in southwestern China. Trans R Soc Trop Med Hyg. 2013 Oct;107(10):660–5.

30. Desai A, Ravi V, Chandramuki A, Gourie-Devi M. Proliferative Response of Human Peripheral Blood Mononuclear Cells to Japanese Encephalitis Virus. Microbiol Immunol. 1995;

31. Kumar R, Tripathi P, Baranwal M, Singh S, Tripathi S, Banerjee G. Randomized, controlled trial of oral ribavirin for Japanese encephalitis in children in Uttar Pradesh, India. Clin Infect Dis. 2009;

32. Zhang X, Hou F, Li X, Zhou L, Liu Y, Zhang T. Study of surveillance data for class B notifiable disease in China from 2005 to 2014. Int J Infect Dis. 2016;

33. Kumar R, Mathur A, Kumar A, Sethi GD, Sharma S, Chaturvedi UC. Virological investigations of acute encephalopathy in India. Arch Dis Child. 1990;

34. Misra UK, Kalita J. Seizures in Japanese encephalitis. J Neurol Sci. 2001;

35. Kumar P, Pisudde PM, Sarthi PP, Sharma MP, Keshri VR. Status and trend of acute encephalitis syndrome and Japanese encephalitis in Bihar, India. Natl Med J India. 2017;

36. McNaughton H, Singh A, Khan SA. An outbreak of Japanese encephalitis in a non-endemic region of north-east India. J R Coll Physicians Edinb. 2018;48(1):25–9.

37. Libraty DH, Nisalak A, Endy TP, Suntayakorn S, Vaughn DW, Innis BL. Clinical and immunological risk factors for severe disease in Japanese encephalitis. Trans R Soc Trop Med Hyg. 2002;96(2):173–8.

38. Basumatary LJ, Raja D, Bhuyan D, Das M, Goswami M, Kayal AK. Clinical and radiological spectrum of Japanese encephalitis. J Neurol Sci. 2013;325(1–2):15–21.

39. Baruah HC, Biswas D, Patgiri D, Mahanta J. Clinical outcome and neurological sequelae in serologically confirmed cases of Japanese encephalitis patients in Assam, India. Indian Pediatr. 2002;39(12):1143–8.

40. Rayamajhi A, Singh R, Prasad R, Khanal B, Singhi S. Clinico-laboratory profile and outcome of Japanese encephalitis in Nepali children. Ann Trop Paediatr. 2006;26(4):293–301.

41. Ompusunggu S, Hills SL, Maha MS, Moniaga VA, Susilarini NK, Widjaya A, et al. Confirmation of Japanese encephalitis as an endemic human disease through sentinel surveillance in Indonesia. Am J Trop Med Hyg. 2008;79(6):963–70.

42. Ravi V, Parida S, Desai A, Chandramuki A, Gourie-Devi M, Grau GE. Correlation of tumor necrosis factor levels in the serum of cerebrospinal fluid with clinical outcome in Japanese encephalitis patients. J Med Virol. 1997;

43. Kumar R, Tripathi P, Singh S, Bannerji G. Clinical features in children hospitalized during the 2005 epidemic of Japanese encephalitis in Uttar Pradesh, India. Clin Infect Dis. 2006 Jul;43(2):123–31.

44. Desai A, Ravi V, Chandramuki A, Gourte-Devi M. Detection of immune complexes in the CSF of Japanese encephalitis patients: Correlation of findings with outcome. Intervirology. 1994;

45. Hoke CH, Vaughn DW, Nisalak A, Intralawan P, Poolsuppasit S, Jongsawas V, et al. Effect of High-Dose Dexamethasone on the Outcome of Acute Encephalitis Due to Japanese Encephalitis Virus. J Infect Dis. 1992 Apr;165(4):631–7.

46. Patgiri SJ, Borthakur AK, Borkakoty B, Saikia L, Dutta R, Phukan SK. An appraisal of clinicopathological parameters in Japanese encephalitis and changing epidemiological trends in upper Assam, India. Indian J Pathol Microbiol. 2014;

47. Jain P, Jain A, Kumar A, Prakash S, Khan DN, Singh KP, et al. Epidemiology and etiology of acute encephalitis syndrome in North India. Jpn J Infect Dis. 2014;67(3):197–203.

48. Touch S, Hills S, Sokhal B, Samnang C, Sovann L, Khieu V, et al. Epidemiology and burden of disease from Japanese encephalitis in Cambodia: Results from two years of sentinel surveillance. Trop Med Int Heal. 2009;

49. Solomon T, Dung NM, Wills B, Kneen R, Gainsborough M, Diet TV, et al. Interferon alfa-2a in Japanese encephalitis: A randomised double-blind placebo-controlled trial. Lancet. 2003;361(9360):821–6.

50. Kalita J, Misra UK, Mani VE, Bhoi SK. Can we differentiate between herpes simplex encephalitis and Japanese encephalitis? J Neurol Sci. 2016;366:110–5.

51. Han N, Adams J, Fang W, Liu SQ, Rayner S. Investigation of the genotype III to genotype I shift in Japanese encephalitis virus and the impact on human cases. Virol Sin. 2015;30(4):277–89.

52. Zheng Y, Li M, Wang H, Liang G. Japanese encephalitis and Japanese encephalitis virus in mainland China. Reviews in Medical Virology. 2012.

53. Lam K, Tsang OTY, Yung RWH, Lau KK. Japanese encephalitis in Hong Kong. Hong Kong Medical Journal. 2005.

54. Olsen SJ, Supawat K, Campbell AP, Anantapreecha S, Liamsuwan S, Tunlayadechanont S, et al. Japanese encephalitis virus remains an important cause of encephalitis in Thailand. Int J Infect Dis. 2010;

55. Ravi V, Vanajakshi S, Gowda A, Chandramuki A. Laboratory diagnosis of japanese encephalitis using monoclonal antibodies and correlation of findings with the outcome. J Med Virol. 1989;

56. Dev V, Sharma V, Barman K. Mosquito-borne diseases in Assam, north-east India: current status and key challenges. WHO South-East Asia J Public Heal. 2015;

57. Maha MS, Moniaga VA, Hills SL, Widjaya A, Sasmito A, Hariati R, et al. Outcome and extent of disability following Japanese encephalitis in Indonesian children. Int J Infect Dis. 2009;13(6):389–93.

58. Saxena V, Dhole TN. Preventive strategies for frequent outbreaks of Japanese encephalitis in Northern India. Journal of Biosciences. 2008.

59. Solomon T, Dung NM, Kneen R, Thao LTT, Gainsborough M, Nisalak A, et al. Seizures and raised intracranial pressure in Vietnamese patients with Japanese encephalitis. Brain. 2002;125(5):1084–93.

60. Avabratha KS, P S, G N, B V, M. V, K B. JAPANESE ENCEPHALITIS IN CHILDREN IN BELLARY KARNATAKA: CLINICAL PROFILE AND SEQUELAE. Int J Biomed Res. 2012;

61. Kumari R, Joshi P. A review of Japanese encephalitis in Uttar Pradesh, India. WHO South-East Asia J Public Heal. 2012;1(4):374.

62. Luo D, Song J, Ying H, Yao R, Wang Z. Prognostic factors of early sequelae and fatal outcome of Japanese encephalitis. Southeast Asian J Trop Med Public Health. 1995;

63. Nayak P, Pradhan A, Mallick R, Sethi S, Patnaik B, Pradhan MM, et al. Japanese Encephalitis Outbreak Among Children in Mayurbhanj, Odisha-India, 2015. Open Forum Infect Dis. 2016;

64. Vajpayee A, Mukherjee MK, Chakraborty AK, Chakraborty MS. Investigation of an outbreak of Japanese encephalitis in Rourkela City (Orissa) during 1989. J Commun Dis. 1991;

65. Wakai S. Scourge of Japanese encephalitis in southwestern Nepal [12]. Lancet. 1998.

66. Lee DW, Choe YJ, Kim JH, Song KM, Cho H, Bae GR, et al. Epidemiology of Japanese encephalitis in South Korea, 2007-2010. Int J Infect Dis. 2012;

67. Bom HS, Kang HK, Joh NJ, Kim SJ, Yoon CM, Cho KK, et al. A clinical study of adult Japanese encephalitis in the Chonnam District, Korea, during summer of 1982--a difference between improved and expired cases. Korean J Intern Med. 1986;1(1):21–5.

68. Li X, Cui S, Gao X, Wang H, Song M, Li M, et al. The Spatio-temporal Distribution of Japanese Encephalitis Cases in Different Age Groups in Mainland China, 2004 – 2014. PLoS Negl Trop Dis. 2016 Apr;10(4):e0004611.

69. Yin Z, Wang H, Yang J, Luo H, Li Y, Hadler SC, et al. Japanese encephalitis disease burden and clinical features of Japanese encephalitis in four cities in the People’s Republic of Chin. Am J Trop Med Hyg. 2010;

70. Rayamajhi A, Singh R, Prasad R, Khanal B, Singhi S. Study of Japanese encephalitis and other viral encephalitis in Nepali children. Pediatr Int. 2007;49(6):978–84.

71. Ding D, Hong Z, Zhao SJ, Clemens JD, Zhou B, Wang B, et al. Long-term disability from acute childhood Japanese encephalitis in Shanghai, China. Am J Trop Med Hyg. 2007;77(3):528–33.

72. Yen NT, Duffy MR, Hong NM, Hien NT, Fischer M, Hills SL. Surveillance for Japanese encephalitis in Vietnam, 1998-2007. Am J Trop Med Hyg. 2010;

73. Parida M, Dash PK, Tripathi NK, Ambuj, Sannarangaiah S, Saxena P, et al. Japanese encephalitis Outbreak, India, 2005. Emerg Infect Dis. 2006;

74. Ming CK, Swe T, Thaung U, Lwin TT. Recent outbreaks of Japanese encephalitis in Burma. Southeast Asian J Trop Med Public Health. 1977;

75. Bista MB, Banerjee MK, Shin SH, Tandan JB, Kim MH, Sohn YM, et al. Efficacy of single-dose SA 14-14-2 vaccine against Japanese encephalitis: A case control study. Lancet. 2001;

76. Joshi, AB; Banjara MR; Bhatta LR; Wierzba T. Status and Trend of Japanese Encephalitis Epidemics in Nepal : A Five-Year Retrospective Review. J Nepal Health Res Counc. 2004;

77. Gupta N, Hossain S, Lal R, Das BP, Venkatesh S, Chatterjee K. Epidemiological profile of Japanese encephalitis outbreak in Gorakhpur, UP in 2004. J Commun Dis. 2005;

78. Hazarika NC. Project study on Japanese encephalitis vaccination at Gogamukh, Assam. Vol. 28, Indian pediatrics. 1991. p. 1029–34.

79. Sharma J (Dibrugarh U, Das J, Pathak A. Surveillance of Acute encephalitis syndrome in Lakhimpur District of Assam: January 2008-October 2012.

80. Singh H, Singh N, Mall RK. Japanese Encephalitis and Associated Environmental Risk Factors in Eastern Uttar Pradesh: A time series analysis from 2001 to 2016. Acta Trop. 2020;
